# Synthesis and Pharmacological Characterization of 2-Aminoethyl Diphenylborinate (2-APB) Derivatives for Inhibition of Store-Operated Calcium Entry (SOCE) in MDA-MB-231 Breast Cancer Cells

**DOI:** 10.3390/ijms21165604

**Published:** 2020-08-05

**Authors:** Achille Schild, Rajesh Bhardwaj, Nicolas Wenger, Dominic Tscherrig, Palanivel Kandasamy, Jan Dernič, Roland Baur, Christine Peinelt, Matthias A. Hediger, Martin Lochner

**Affiliations:** 1 Institute of Biochemistry and Molecular Medicine, University of Bern, Bühlstrasse 28, 3012 Bern, Switzerland; achille.schild@dcb.unibe.ch (A.S.); dominic.tscherrig@gmail.com (D.T.); roland.baur@ibmm.unibe.ch (R.B.); christine.peinelt@ibmm.unibe.ch (C.P.); 2 Department of BioMedical Research, University of Bern, Murtenstrasse 35, 3008 Bern, Switzerland; rajesh.bhardwaj@dbmr.unibe.ch (R.B.); nicolas.wenger@students.unibe.ch (N.W.); palanivel.kandasamy@dbmr.unibe.ch (P.K.); jan.dernic@students.unibe.ch (J.D.)

**Keywords:** store-operated calcium entry, inhibitors, synthetic chemistry, 2-APB, structure–activity relationship, breast cancer cells, calcium imaging assay, patch-clamp electrophysiology

## Abstract

Calcium ions regulate a wide array of physiological functions including cell differentiation, proliferation, muscle contraction, neurotransmission, and fertilization. The endoplasmic reticulum (ER) is the major intracellular Ca^2+^ store and cellular events that induce ER store depletion (e.g., activation of inositol 1,4,5-triphosphate (IP_3_) receptors) trigger a refilling process known as store-operated calcium entry (SOCE). It requires the intricate interaction between the Ca^2+^ sensing stromal interaction molecules (STIM) located in the ER membrane and the channel forming Orai proteins in the plasma membrane (PM). The resulting active STIM/Orai complexes form highly selective Ca^2+^ channels that facilitate a measurable Ca^2+^ influx into the cytosol followed by successive refilling of the ER by the sarcoplasmic/endoplasmic reticulum calcium ATPase (SERCA). STIM and Orai have attracted significant therapeutic interest, as enhanced SOCE has been associated with several cancers, and mutations in STIM and Orai have been linked to immunodeficiency, autoimmune, and muscular diseases. 2-Aminoethyl diphenylborinate (2-APB) is a known modulator and depending on its concentration can inhibit or enhance SOCE. We have synthesized several novel derivatives of 2-APB, introducing halogen and other small substituents systematically on each position of one of the phenyl rings. Using a fluorometric imaging plate reader (FLIPR) Tetra-based calcium imaging assay we have studied how these structural changes of 2-APB affect the SOCE modulation activity at different compound concentrations in MDA-MB-231 breast cancer cells. We have discovered 2-APB derivatives that block SOCE at low concentrations, at which 2-APB usually enhances SOCE.

## 1. Introduction

Store-operated calcium entry (SOCE) is a ubiquitous pathway by which cells regulate basal calcium (Ca^2+^), refill intracellular Ca^2+^ stores, and maintain Ca^2+^ signaling to control diverse functions such as gene expression, cell proliferation, migration, and differentiation [1]. The stimulation of cell surface receptors coupled to phosphatidylinositol 4,5-bisphosphate (PI(4,5)P_2_) hydrolysis produces inositol 1,4,5-trisphosphate (IP_3_), a second messenger which upon binding to IP_3_ receptors (IP_3_Rs) on the endoplasmic reticulum (ER) membrane, releases Ca^2+^ from the ER. The depletion of the ER Ca^2+^ stores, triggers the activation of ER Ca^2+^-sensing stromal interaction molecules (STIM1 and STIM2) that are embedded in the ER membrane which in turn activate Orai channels (Orai1, Orai2, and Orai3) at the plasma membrane (PM). STIM1 and Orai1 were identified as the predominant isoforms of STIMs and Orais that mediate SOCE in the majority of cell types [2,3,4,5]. Upon activation, structural rearrangements in STIM expose the cytosolic STIM Orai-activating region (SOAR). SOAR physically opens the PM Orai channels, resulting in Ca^2+^ entry into the cytosol [1]. The STIM/Orai-mediated Ca^2+^ entry, electrophysiologically characterized as Ca^2+^ release-activated Ca^2+^ current (*I*_CRAC_) [6], thus allows the acute refilling of the ER Ca^2+^ stores [7]. Experimentally, sarcoplasmic/endoplasmic reticulum Ca^2+^ ATPase (SERCA) blocker thapsigargin (Tg) [8] is widely used for passive depletion of ER Ca^2+^ stores and subsequent activation of SOCE.

Gain-of-function or loss-of-function mutations in STIM1 or Orai1, or alterations in expression ratios of STIM and Orai isoforms as well as aberrations in other regulatory mechanisms of SOCE pathway lead to its dysfunction [9,10,11]. The disruption of SOCE has various pathophysiological implications such as muscle, immune, and neurodegenerative disorders as well as cancer [12,13,14,15,16]. Breast cancer metastasis has been linked to STIM1/Orai1-mediated Ca^2+^ influx [17] and several studies also highlighted the upregulation of STIM1 and Orai1 in basal breast cancers [18,19]. Our understanding on the remodeling of SOCE in breast cancer cells has increased over the years and has recently been reviewed [20]. In addition, SOCE has been shown to control the proliferation, migration, invasion, and metastasis in different cancer cell lines [21,22]. Given the role of SOCE in tumor progression [16], developing potent and selective SOCE modulators is of high therapeutic interest.

**Figure 1:**
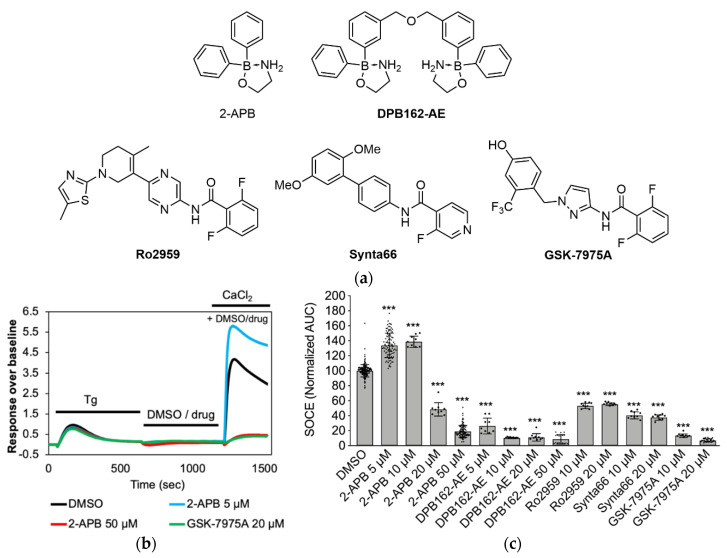
Pharmacological characterization of store-operated calcium entry (SOCE) in MDA-MB-231 cells by fluorometric imaging plate reader (FLIPR) assay. (**a**) Structures of known SOCE inhibitors used in the validation of the assay. (**b**) Recorded intracellular calcium signal over the course of the assay. Black bars above traces indicate duration of applications. MDA-MB-231 cells were incubated with thapsigargin (Tg, 1 μM, 10 min) in nominal calcium-free (NCF) buffer, followed by DMSO (negative control) or drug application (in NCF) for 10 min and finally, addition of 2 mM extracellular CaCl_2_. Note how low concentration (5 μM) of 2-APB enhanced SOCE (blue trace) whereas at high concentration (50 μM) SOCE is blocked (red trace). (**c**) Quantification of calcium influx after CaCl_2_ application (area under the curve (AUC)), normalized to DMSO control (*n* ≥ 9 for all data points). Data shown are mean ± SD. Statistical significance compared to DMSO control is indicated as “***” for *p* ≤ 0.001.

Several chemical inhibitors [23,24,25,26,27] as well as potentiators of SOCE [28,29,30] have been reported. However, 2-aminoethyl diphenylborinate (2-APB, Figure 1) remains the best characterized compound used to modulate store-operated CRAC channels, despite of its effects on other known targets [31,32,33,34,35,36,37,38,39,40,41,42,43,44]. Although originally described as an inhibitor of IP_3_ receptors [45,46], it was later shown that inhibition of SOCE and *I*_CRAC_ by 2-APB is independent of its action on IP_3_ receptors [36,47,48,49,50,51]. 2-APB is known to exert a very interesting dose-dependent bimodal effect on SOCE. 2-APB when applied in ≤10 µM concentration potentiates whereas ≥30 µM concentration blocks SOCE and STIM1/Orai1-mediated *I*_CRAC_ in various cell lines such as HEK293, human Jurkat T cells, chicken DT40 B cells, and rat basophilic leukemia cells [42,47,49,50,52,53,54,55,56]. Recent studies have shed some light on the underlying mechanisms of action of potentiation and inhibition of SOCE/*I*_CRAC_ by 5 µM and 50 µM 2-APB, respectively [57,58,59]. 2-APB not only acts directly on the Orai1 channel for the inhibition of SOCE, but also enhances the intramolecular interactions within STIM1, which leads to the disruption of the functional coupling between STIM1 and Orai1 [59]. On the other hand, 5 µM 2-APB potentiates CRAC channels by a direct dilation of the open pore of Orai1 [57]. The other STIM/Orai isoforms as well as heteromeric channels formed by Orai1/Orai3 have been shown to respond differently to 2-APB [53,55,60,61,62,63,64,65].

Several derivatives of 2-APB that modulate SOCE have been reported previously [28,52,54,58,66,67,68]. Among these derivatives, **DPB162-AE** (Figure 1), a dimeric, more specific as well as more potent variant of 2-APB, did not exert the typical dose-dependent potentiating effect on SOCE [54,58]. Interestingly, in addition to SOCE inhibition, **DPB162-AE** (1–10 µM) also released Ca^2+^ from the ER stores of pancreatic acinar cells as well as HEK293, HeLa and B-cell lymphoma SU-DHL-4 cells [69,70]. In HEK293 and HeLa cells, IP_3_Rs were partly responsible for this **DPB162-AE** elicited ER Ca^2+^ release, with a likely additional role of other ER Ca^2+^ release channels [69]. A similar IP_3_R-dependent release of ER Ca^2+^ stores was reported in DT40 cells by 2-APB at concentrations ranging from 10 to 75 µM [56]. The release of ER Ca^2+^ by 2-APB is most likely cell type-dependent, as pretreatment of Chinese hamster ovary-K1 cells with 100 µM 2-APB did not affect the thapsigargin-mediated store-depletion [71].

In this study, we used the MDA-MB-231 breast cancer cell line to screen our newly developed 2-APB analogues for their action on SOCE, using a fluorometric imaging plate reader (FLIPR)-based Ca^2+^ imaging assay. FLIPR assays with MDA-MB-231 cells have been used in the past for discovery of SOCE modulators [26,30,72]. Earlier reports using RNA silencing have shown that SOCE in MDA-MB-231 cells is conducted by STIM1 and Orai1 [18,26,73]. In addition, it was shown that 2-APB at concentrations ≥30 µM blocks SOCE in MDA-MB-231 cell line [73,74,75]. Herein, we report new derivatives of 2-APB, that are more potent than 2-APB in blocking SOCE in MDA-MB-231 cells. We also report the effect of our new 2-APB derivatives on store-depletion as well as proliferation of MDA-MB-231 cells.

## 2. Results and Discussion

### 2.1. Synthesis of 2-APB Analogues

**Scheme 1:**
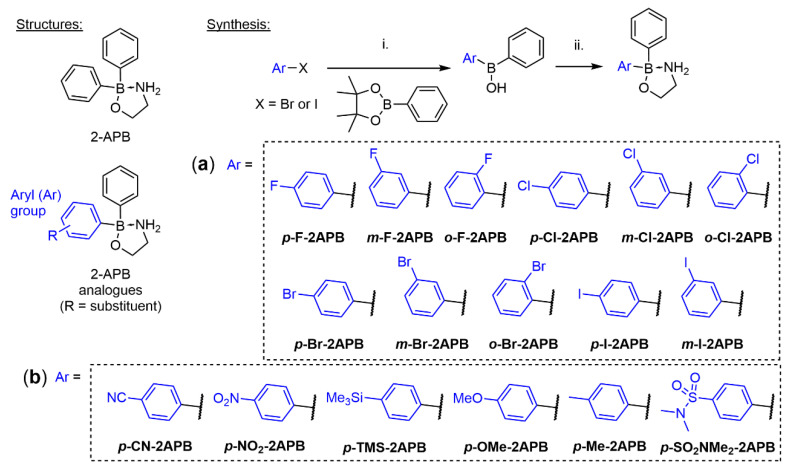
Synthesis of (**a**) mono-halogenated and (**b**) *para*-substituted 2-APB analogues. Reagents and conditions: (i) *n*-BuLi or *t*-BuLi, Et_2_O, or THF, −78 °C; then phenylboronic acid pinacol ester, Et_2_O or THF, −78 °C to rt; then 1 M HCl, rt. For the synthesis of ***o*-Br-2APB** toluene/THF 4:1 was used as solvent. (ii) 2-Aminoethanol, EtOH, MeCN, or THF, rt or reflux. Isolated yields over two steps: 6–66%. ***p*-NO_2_-2APB** was synthesized via different route (see text and Materials and Methods).

The mono-halogenated 2-APB analogues were synthesized by following our previously optimized route [67]. More specifically, mono-halogenated aryl bromides or iodides were transformed into their corresponding aryl lithium species by halogen-lithium exchange at low temperature and then reacted with phenylboronic acid pinacol ester (Scheme 1). This gave the crude aryl phenyl borinic acids after work up and quick flash chromatography purification, which were subsequently esterified with 2-aminoethanol to yield the stable, mono-halogenated 2-APB analogues. This protocol allowed the synthesis of almost all possible mono-halogenated 2-APB analogues (Scheme 1a) in isolated yields ranging from 6 to 65%. For the synthesis of ***o*-Br-2APB** it was important to use a mixture of toluene/THF 4:1 [76] in the lithiation and subsequent borination step, in order to significantly reduce the formation of a biphenyl side product that is very difficult to separate from the desired product (Appendix A). We were unable to isolate the *ortho*-iodo 2-APB derivative ***o*-I-2APB**. All our conditions gave inseparable mixtures of *ortho*-iodophenyl borinic acid and significant amounts of the side product (Appendix A).

We were also interested in accessing 2-APB congeners with small substituents other than halogens in order to probe the influence of size and electron-withdrawing or electron-donating properties at the *para*-position on SOCE activity. These compounds were generated using the same route as above, except for ***p*-NO_2_-2APB**, and yielded 2-APB analogues shown in Scheme 1b in 8–66%. The *para*-nitro 2-APB analogue was obtained by reacting 4-nitrophenylborinic acid ester with phenyllithium, followed by borinate formation of the isolated borinic acid addition product.

For comparison, we also synthesized the dimeric 2-APB compound **DPB162-AE**, reported by the Mikoshiba group [54,77], but slightly adapted their procedure (Scheme S1). Recently reported heterocyclic SOCE blockers **GSK-7975A** and **Synta66** were also synthesized (Schemes S2 and S3) applying procedures in the corresponding patents [78,79]. Notably, we have obtained a crystal structure for **GSK-7975A** which was not described before (Figure S1).

**Scheme 2:**
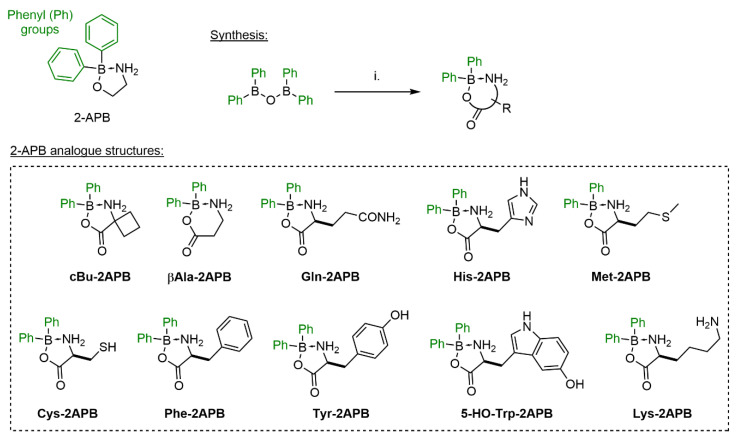
Synthesis of 2-APB amino acid analogues. Reagents and conditions: (i) amino acid, EtOH, reflux. Isolated yields: 16%-quantitative.

Others and we have shown previously that amino acid adducts of 2-APB, forming acyloxyboranes, are less dynamic in solution than the corresponding amino alcohol esters [67,80]. Furthermore, our studies indicated that amino acid 2-APB adducts with large side chain might block SOCE in HEK293 cells efficiently. In order to study this more systematically, we expanded our 2-APB library in this direction and synthesized several amino acid complexes shown in Scheme 2. Conveniently, such derivatives can be synthesized in one step from diphenylborinic anhydride.

The cyclic nature of the synthesized 2-APB derivatives in solution is apparent from their ^1^H and ^11^B NMR spectra (in DMSO-*d*_6_). Donation of electron density of the amino alcohol or amino acid nitrogen to the electron deficient boron atom leads to deshielding of the N-H protons and thus they are low field shifted to 6.1–7.2 ppm. Likewise, the ^11^B chemical shift of the central boron atom is recorded at 3.7–4.7 ppm for all isolated 2-APB derivatives, except for **βAla-2APB** which is at 2.05 ppm. For comparison, the “uncoordinated” boron atom in diphenyl borinic acid (Ph_2_B(OH)) shows a ^11^B chemical shift of 45 ppm [67]. The cyclic form of the 2-APB derivatives is also visible in the crystal structures of **cBu-2APB**, ***p*-SO_2_NMe_2_-2APB**, ***o*-F-2APB**, and ***o*-Cl-2APB** (Figures S2–S5). For the latter two derivatives the phenyl and *ortho*-substituted phenyl groups are mutually disordered in the structure (Figures S4 and S5).

### 2.2. Influence of 2-APB Analogues on Store-Operated Ca^2+^ Entry in MDA-MB-231 Breast Cancer Cells

For evaluating the activity of the synthetic 2-APB analogues on SOCE, we used a fluorescence-based calcium influx assay into the triple negative breast cancer cells MDA-MB-231. In our assay protocol, 1 μM thapsigargin in nominal calcium-free (NCF) buffer was first applied to the cells for several minutes, which irreversibly blocks SERCA, passively depletes the intracellular ER-stores and initiates the SOCE cascade. This was followed by compound application in NCF buffer for several minutes and a subsequent application of 2 mM extracellular CaCl_2_ to measure external calcium entry through CRAC channels (Figure 1b). We validated our assay using known SOCE borinic acid inhibitors 2-APB, **DPB162-AE** and heterocyclic compounds **GSK-7975A**, **Synta66,** and **Ro2959 [81]** (Figure 1a) at concentrations between 5 and 20 μM (Figure 1c). As reported previously in other cell lines, 2-APB showed a similar bimodal effect on SOCE in MDA-MB-231 cells wherein it enhanced Ca^2+^ entry at low concentrations (5 and 10 μM) and blocked at higher concentrations (partial block at 20 and maximum block at 50 μM) [49,56]. The dimeric 2-APB derivative **DPB162-AE** exerted a comparable inhibition of SOCE at lower concentration, as 2-APB at 50 µM, confirming its higher activity than 2-APB [77]. The inhibition of SOCE at 10 μM by **DPB162-AE** was even stronger than by heterocyclic **Ro2959** and **Synta66**. Even doubling the concentration of the latter two compounds did not increase the SOCE block. In our assay, **GSK-7975A** was the most potent heterocyclic SOCE blocker. We were surprised at the somewhat low activity of **Ro2959** and **Synta66**. The inhibition activity of these heterocyclic compounds has been previously characterized by measuring the influence on the calcium release-activated calcium current (*I*_CRAC_) using electrophysiology.

The reported values for **Synta66** (IC_50_ 1.4 μM, *I*_CRAC_ in RBL cells) [82,83], **GSK-7975A** (IC_50_ 4 μM, *I*_CRAC_ in HEK293 cells) [84], and **Ro2959** (IC_50_ 0.4 μM, *I*_CRAC_ in RBL-2H3 cells) [85] suggest that they should fully block calcium entry at the concentrations applied in our SOCE assay in MDA-MB-231 cells. The electrophysiology studies have also revealed that all three heterocyclic compounds have a slow onset of inhibition, requiring cells to be preincubated for long periods (e.g., >1 h for **Synta66**). Indeed, when we preincubated 0.3–0.5 μM **GSK-7975A** or 1.25–10 µM **Synta66** for 20 min before the thapsigargin application (30 min total incubation time), SOCE in MDA-MB-231 cells was inhibited by 50% or 80%, respectively, which is in the range of the reported IC_50_ values (Figure S6).

Next, we investigated whether the SOCE observed in MDA-MB-231 cells that we could block by inhibitors shown in Figure 1 is conducted by STIM1 and Orai1. Indeed, knock-out of either STIM1 or Orai1 in MDA-MB-231 cells using CRISPR/Cas9 gene editing technique, led to significantly diminished SOCE (Figure S7). This suggests that SOCE in these cells is mainly facilitated by Orai1/STIM1, confirming the RNA interference studies by Motiani et al. [73].

**Figure 2:**
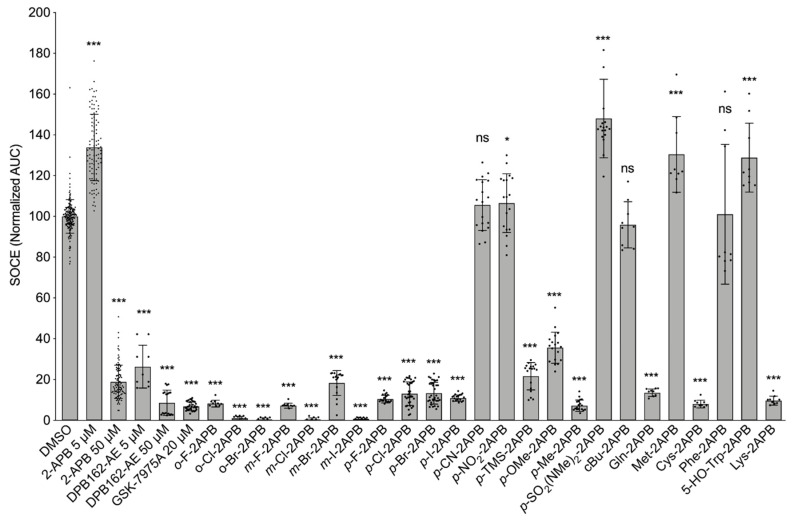
Screening of synthetic 2-APB analogues on SOCE in MDA-MB-231 cells using the FLIPR-based SOCE assay. Quantification of calcium influx after CaCl_2_ application (AUC), normalized to DMSO control (*n* ≥ 9 for all data points). Data shown are mean ± SD. All compounds were tested at 50 µM concentration, unless otherwise noted. Statistical significance compared to DMSO control is indicated as “***” for *p* ≤ 0.001, “*” for 0.01 < *p* ≤ 0.05 and “ns” not significant for *p* > 0.05.

After having validated the SOCE assay, we first tested all our synthetic 2-APB analogues at a single concentration (50 µM, Figure 2). Our data show that all halogenated compounds efficiently blocked SOCE, some even exerted a more pronounced reduction of the Ca^2+^ entry than the parent compound (e.g., ***m*-Cl-2APB**, ***o*-Cl-2APB**, ***o*-Br-2APB**, and ***m*-I-2APB**). Compared to the *para*-halogenated analogues, most of the other *para*-substituted congeners did not block SOCE significantly. In fact, 2-APB derivatives with large, polarized substituents in the *para*-position rather enhanced SOCE (e.g., ***p*-CN-2APB**, ***p*-NO_2_-2APB**, and ***p*-SO_2_NMe_2_-2APB**). The amino acid 2-APB derivatives **βAla-2APB**, **His-2APB,** and **Tyr-2APB** could not be tested because of very poor solubility in the assay buffer. Only few derivatives with polar side chains (e.g., **Gln-2APB**, **Lys-2APB**, and **Cys-2APB**) were able to inhibit SOCE in MDA-MB-231 cells (Figure 2).

**Figure 3:**
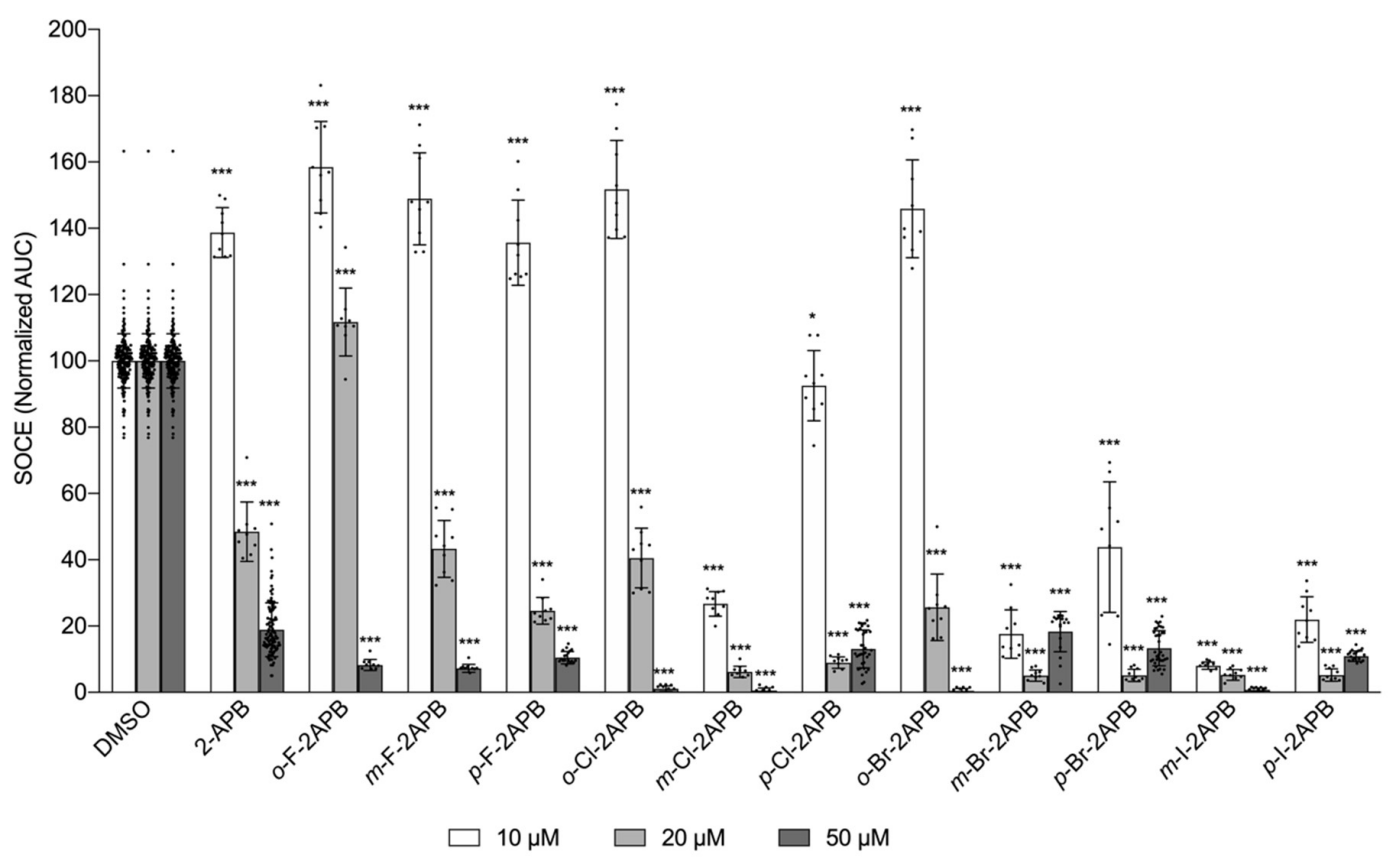
Select halogenated 2-APB analogues block SOCE more potently at lower concentration than 2-APB. Influence on SOCE in MDA-MB-231 cells after prior depletion of the intracellular Ca^2+^ store, using the FLIPR-based SOCE assay. Data show quantification of Ca^2+^ entry (AUC, normalized to DMSO control) after extracellular application of 2 mM CaCl_2_. Compound concentrations: 10 µM (white bars), 20 µM (grey bars), 50 µM (dark grey bars). All data points from n ≥ 9 independent experiments. Data shown are mean ± SD. Statistical significance is compared to the respective DMSO control and indicated as “***” for *p* ≤ 0.001 and “*” for 0.01 < *p* ≤ 0.05.

Given the encouraging results, we turned our attention to the mono-halogenated 2-APB analogues. In order to test if any of them have higher SOCE blocking potency than 2-APB, we applied them at lower concentrations (10 and 20 µM, Figure 3). At 10 µM compound concentration all fluorinated 2-APB derivatives enhanced SOCE, similar to 2-APB at the same concentration. This effect was also observed for some of the chloro-compounds, depending on the substitution position, whereas the bromo- and iodo-analogues were overall rather inhibiting SOCE at this low concentration. In terms of the halogen substitution position on one of the phenyl rings of 2-APB, the chloro, bromo, and iodo 2-APB derivatives affect SOCE with the following trend: *ortho* enhances SOCE, *meta* blocks efficiently, and *para* shows only partial or no block of SOCE. This efficient block of SOCE by the *meta*-substituted derivatives is also apparent at 20 µM compound concentration, at which 2-APB only partially blocks (Figure 3).

When comparing the SOCE blocking effect at 10 µM compound concentration with the different halogen substituents at the same position, all *ortho*-halogenated derivatives did not block, but rather potentiated SOCE. For the *meta*- and *para*-positions, inhibiting potency increased with increasing size and decreasing electronegativity (***m*-F-2APB** << ***m*-Cl-2APB** < ***m*-Br-2APB** < ***m*-I-2APB** and ***p*-F-2APB** << ***p*-Cl-2APB** < ***p*-Br-2APB** < ***p*-I-2APB**).

### 2.3. Store Depletion in MDA-MB-231 Breast Cancer Cells by 2-APB Analogues

**Figure 4:**
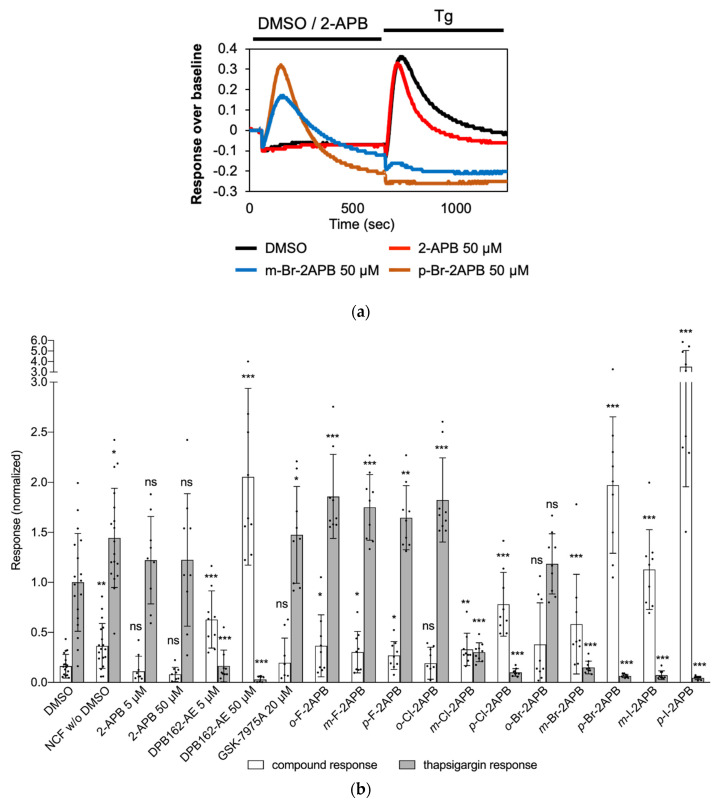
Store-depletion by selected 2-APB analogues in MDA-MB-231 cells. (**a**) Examples of recorded intracellular calcium responses for ***m*-Br-2APB** and ***p*-Br-2APB** in FLIPR assay. Black bars above traces indicate duration of compound applications. MDA-MB-231 cells were incubated with compounds (50 µM, unless otherwise noted) in NCF for 10 min, followed by thapsigargin (Tg, 2.5 µM) application in NCF buffer. (**b**) Quantifications of Ca^2+^ mobilization effect from intracellular stores, using the slopes of the curves, by compounds (white bars) and by subsequent thapsigargin (dark bars) application. Normalized to post-thapsigargin response by DMSO, *n* ≥ 9 for all data points. Data shown are mean ± SD. Statistical significance is compared to the respective DMSO control and indicated as “***” for *p* ≤ 0.001, “**” for 0.001 < *p* ≤ 0.01 and “*” for 0.01 < *p* ≤ 0.05. ns, not significant for *p* > 0.05.

We also investigated if the synthesized 2-APB derivatives have an effect on intracellular Ca^2+^ stores, as it has been observed in some cell lines with **DPB-162AE** [69] and non-boron based SOCE inhibitors [72]. To this end, we treated MDA-MB-231 cells with compounds at 50 µM in NCF buffer first and then added thapsigargin to deplete the stores (Figure 4). The FLIPR recordings of intracellular [Ca^2+^] show that 2-APB, for instance, does not lead to any detectable mobilization of Ca^2+^ from intracellular stores of MDA-MB-231 cells. Conversely, ***p*-Br-2APB** produced a substantial Ca^2+^ release signal during application and subsequent store depletion by thapsigargin was unable to mobilize more calcium. Quantification of this effect revealed that dimeric 2-APB compound **DPB162-AE** mobilizes stored calcium significantly at high concentration compared to 2-APB. From the halogenated compounds, ***p*-Br-2APB** and ***p*-I-2APB** were as strong or even stronger Ca^2+^ mobilizers than **DPB162-AE**.

The compound-induced (pre-thapsigargin) store depletion increases with increasing size and decreasing electronegativity of the halogen substituent (F < Cl < Br < I), in particular at the *para*-position. Concerning the halogen substitution position, the Ca^2+^ mobilization effect was strongest for the *para*-compounds, less for the *meta-*, and not substantial for the *ortho*-analogues.

**Figure 5:**
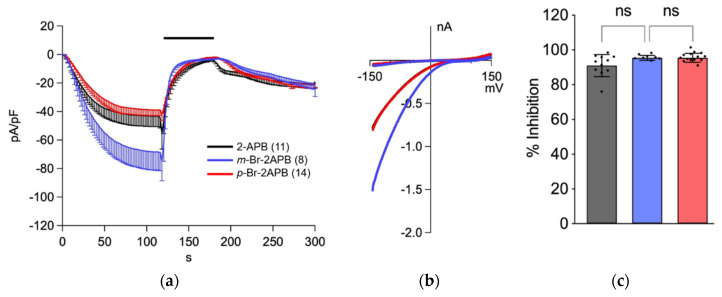
Inhibition of *I*_CRAC_ in HEK293 cells overexpressing human STIM1/Orai1 by 2-APB (black traces) and analogues ***m*-Br-2APB** (blue) and ***p*-Br-2APB** (red). (**a**) *I*_CRAC_ was evoked with 20 mM BAPTA and 50 µM IP_3_ in the patch pipette, currents at −130 mV were normalized to cell capacity and plotted as current density (CD) versus time. Upon *I*_CRAC_ development, 2-APB (black), ***m*-Br-2APB** (blue) or ***p*-Br-2APB** (red) were applied from 120–180 s (black bar above traces). (**b**) I-V curves extracted from recordings in (**a**) when *I*_CRAC_ had developed at *t* = 118 s. (**c**) Inhibition of *I*_CRAC_ was calculated from CD at 180 s normalized to the point in time before compounds were applied (*t* = 118 s) and plotted as inhibition in %. All compounds applied at 50 µM, number of experiments (*n*) in brackets in panel (**a**). Data shown are mean ± SD. Statistical significance is indicated as ns for not significant (*p* > 0.05).

In addition, we checked the ability of ***m*-Br-2APB** and ***p*-Br-2APB** to block *I*_CRAC_ in HEK293 cells overexpressing STIM1/Orai1 (Figure 5). Both compounds rapidly inhibited *I*_CRAC_ in a similar fashion as the parent compound 2-APB, which suggests that in addition to store-depleting activity (Figure 4) SOCE was blocked as well.

### 2.4. Synthetic 2-APB Analogues Effect MDA-MB-231 Cell Viability

**Figure 6:**
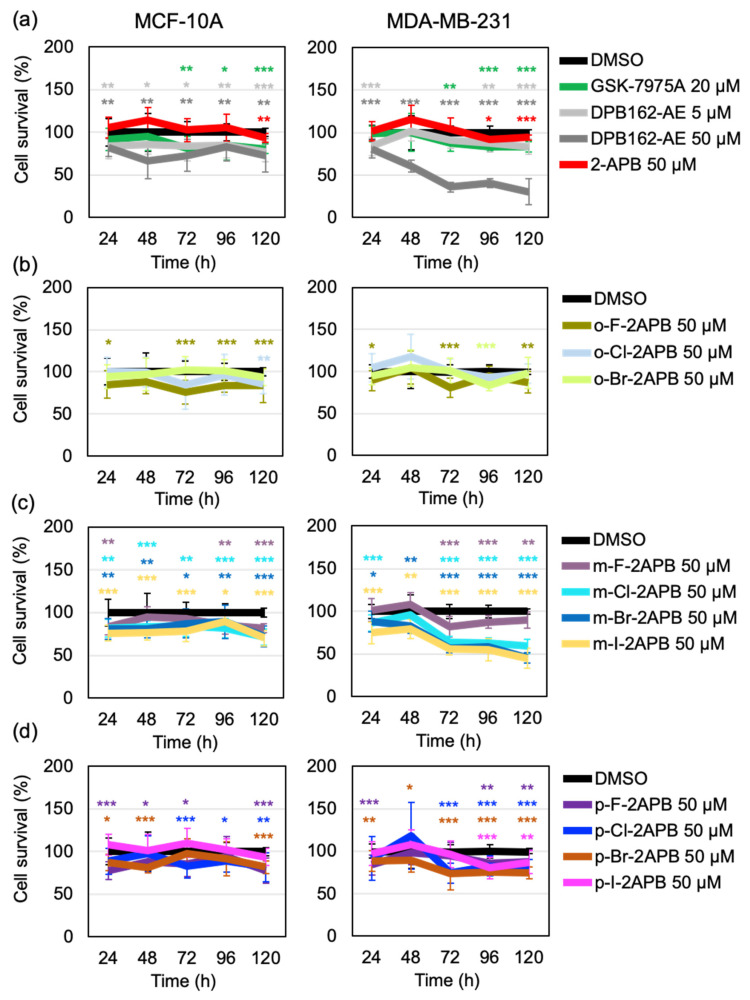
Dose-response curves of 20 µM **GSK-7975A**, 5 µM and 50 µM **DPB162-AE**, and 50 µM 2-APB (**a**), 50 µM ***o*-F-2APB**, ***o*-Cl-2APB**, and ***o*-Br-2APB** (**b**) 50 µM ***m*-F-2APB**, ***m*-Cl-2APB**, ***m*-Br-2APB**, and ***m*-I-2APB** (**c**), and 50 µM ***p*-F-2APB**, ***p*-Cl-2APB**, ***p*-Br-2APB**, and ***p*-I-2APB** (**d**) in non-tumorigenic breast epithelial cell line MCF-10A and triple negative breast cancer cell line MDA-MB-231. Relative cell survival in case of each compound treatment was calculated as a percentage of DMSO control. Data shown are mean ± SD (*n* = 12/compound for each time point) of two independent experiments. Significance levels compared to the DMSO control are indicated as “*” for 0.01 < *p* ≤ 0.05, “**” for 0.001 < *p* ≤ 0.01, or “***” for *p* ≤ 0.001 and not indicated if non-significant (*p* > 0.05).

**Figure 7:**
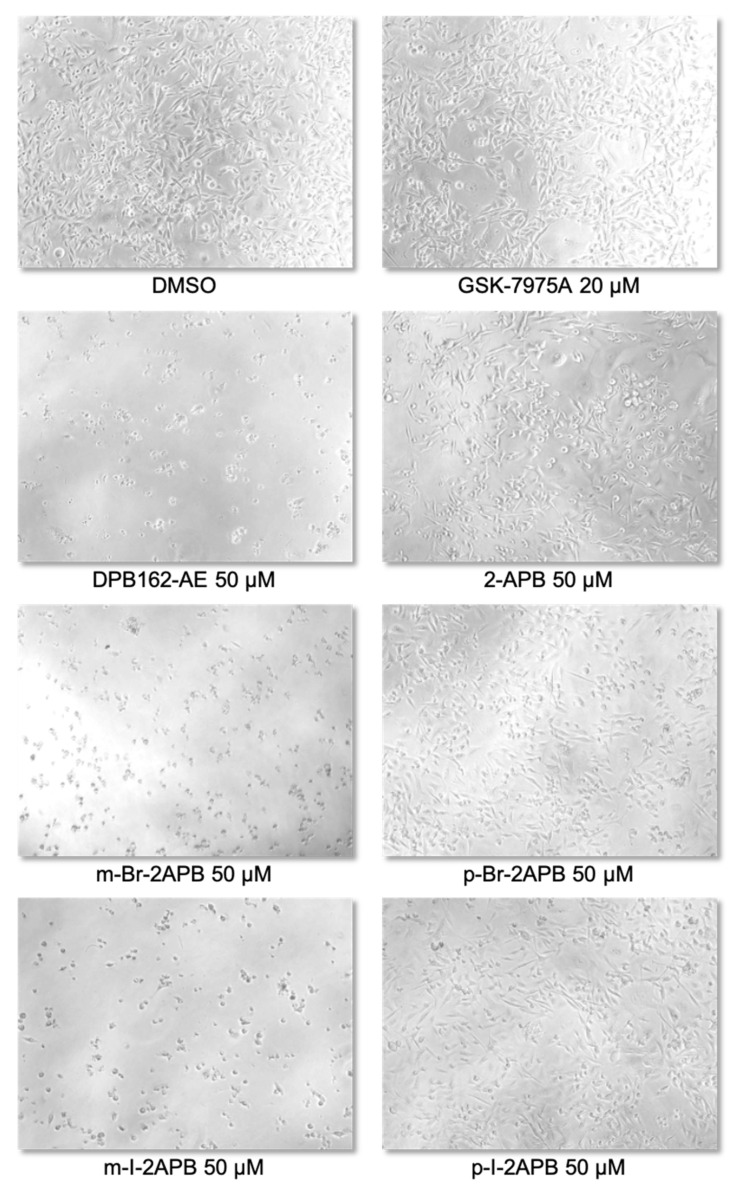
Photomicrographs show the morphological aspects of the cultures of MDA-MB-231 cells after 72 h treatment with control (DMSO) or the indicated compounds. The shown bright-field microscopy images were captured on Nikon Eclipse TiU inverted microscope.

We wondered if the propensity of the 2-APB analogues to mobilize calcium from internal stores is affecting cell viability. MDA-MB-231 cells as well as MCF-10A, a non-tumorigenic mammary epithelial cell line (control), were incubated with 2-APB or halogenated derivatives thereof at 50 µM concentration for different time points ranging from 24 to 120 h. The XTT cell viability assay showed that 2-APB did not have any substantial effect on proliferation of any of the cell lines (Figure 6a). While the more specific SOCE blocker **GSK-7975A** had only minor cytotoxic effect in both cell lines, 50 µM **DPB162-AE** strongly affected the proliferation of MDA-MB-231 cells in comparison to MCF-10A (Figure 6a). On the other hand, the *ortho*- and *para*-halogenated analogues did not show contrasting effects on proliferation of MDA-MB-231 cells compared to MCF-10A cells (Figure 6b,d). Conversely, ***m*-Cl-2APB, *m*-Br-2APB, and *m*-I-2APB** exhibited a stronger cytotoxic effect similar to that of 50 µM **DPB162-AE** on MDA-MB-231 cells compared to MCF-10A cells (Figure 6c). These compounds also induced changes in morphology of MDA-MB-231 cells (Figure 7). The difference between the *ortho*- and *para*-halogenated regioisomers for example, ***p*-Br-2APB** and ***m*-Br-2APB** is particularly striking considering that the bromine atom is moved by only one carbon position (Figure 6 and Figure 7). In any case, the data suggest that compounds which empty internal calcium stores (e.g., ***p*-Br-2APB**) do not influence MDA-MB-231 cell viability much stronger than compounds which empty internal stores very little (e.g., ***p*-F-2APB**). Furthermore, we do not see a direct relation between SOCE inhibitory activity of 2-APB analogues or control compounds (e.g., **GSK-7975A**) and MDA-MB-231 cell proliferation. We cannot rule out the possible off-target effects of our 2-APB analogues in the observed cytotoxicity.

## 3. Materials and Methods

### 3.1. Chemical Synthesis

Commercial solvents and reagents were used without further purification. Dry solvents were obtained by filtration over columns of dried alumina under a positive pressure of argon. The glassware was dried by heating while flushing with argon. In general, reactions were run under argon atmosphere. Flash chromatography was performed on a TeledyneISCO CombiFlash Rf+ (Lincoln, NE, USA) using RediSepRf (Lincoln, NE, USA) pre-packed silica gel columns and cyclohexane/EtOAc gradients to elute compounds, unless otherwise noted. ^1^H, ^11^B, ^13^C, and ^19^F NMR spectra were measured on a Bruker Avance 300 III HD or Avance II 400 spectrometer. Chemical shifts given in ppm, are referenced to residual solvent peaks (^1^H and ^13^C) or internal standard (^11^B and ^19^F), and coupling constants *J* in Hz. (high resolution) mass spectra were recorded on a Thermo Fisher Scientific (Waltham, MA, USA) LTQ Orbitrap XL spectrometer consisting of a linear ion trap (LTQ) featuring a HCD collision cell, coupled to the Orbitrap mass analyzer, equipped with a nanospray ion source (NSI). All X-ray structure determination measurements were made on an Oxford Diffraction SuperNova area-detector diffractometer using mirror optics monochromated Mo *K*α radiation (λ = 0.71073 Å) and Al filtered.

Reference compound **Ro2925** was purchased from AK Scientific and **DPB162-AE**, **Synta66** and **GSK-7975A** were resynthesized (Supplementary Materials) by adapting literature [54] and patent protocols [78,79]. The synthesis and spectroscopic characterization of 2-APB derivatives ***p*-Me-2APB** and ***p*-I-2APB** was reported previously [67].

#### 3.1.1. General Procedure A for the Synthesis of Mono-substituted Phenyl 2-APB Analogues

Dry Et_2_O or THF (60 mL) was cooled to −78 °C and, unless otherwise noted, *t*-BuLi solution (10.2 mmol, 1.7 M in pentane) was added dropwise. The resulting mixture was stirred at −78 °C for 5 min. Then, a solution of iodide or bromide (4.96 mmol) in dry Et_2_O or THF (10 mL) was added to the reaction mixture at a drop rate so that the internal temperature remained below −75 °C. The mixture was stirred for another hour at −78 °C. A solution of phenylboronic acid pinacol ester (4.96 mmol) in dry Et_2_O or THF (10 mL) was then added, again taking care that the internal temperature did not raise above −75 °C. The reaction mixture was allowed to warm up to r.t. overnight and was then quenched by the addition of aq. 1 M HCl (100 mL). The two resulting phases were separated, and the aqueous phase extracted with Et_2_O or EtOAc (2 × 20 mL). The combined organic layers were dried over Na_2_SO_4_, filtered and evaporated. The crude, oily borinic acid was purified by flash chromatography (silica gel, cyclohexane/EtOAc gradient). The resulting purified borinic acid (oil or solid) was dissolved in MeCN or abs. EtOH (5 mL) and 2-aminoethanol (3.48 mmol, 1 equiv. with respect to purified borinic acid) was added to the solution. The resulting solution was stirred at r.t. for 30 min–1 h to produce the corresponding borinic acid 2-aminoethyl ester, which either precipitated or was isolated after removal of the solvent (for details see individual compounds below).

#### 3.1.2. General Procedure B for the Synthesis of 2-APB Amino Acid Analogues

A mixture of amino acid (0.70 mmol) and diphenylborinic anhydride (0.58 mmol) in abs. EtOH (10 mL) was heated at reflux for 30 min–3 h. The clear solution was allowed to cool to r.t. during which the product often precipitated. For some derivatives, further cooling to 0 °C or −20 °C and concentration of the solution was necessary to initiate precipitation or crystallization. The solid products were washed (e.g., with cold EtOH, *n*-hexane or Et_2_O) or recrystallized if necessary (for details see individual compounds below).

#### 3.1.3. 2-(4-Fluorophenyl)-2-phenyl-1,3,2λ^4^-oxazaborolidine (***p*-F-2APB**)

Following general procedure A, 4-fluoroiodobenzene (572 µL, 4.96 mmol) in dry Et_2_O was lithiated with *t*-BuLi (6 mL, 1.7 M in pentane, 10.2 mmol) and reacted with phenylboronic acid pinacol ester (1.01 g, 4.96 mmol). Purification of the crude borinic acid by flash chromatography afforded a brownish oil (663 mg), which was dissolved in MeCN and treated with 2-aminoethanol (210 µL, 3.48 mmol) at r.t. for 30 min. Removing the solvents under reduced pressure and drying the residue under high vacuum afforded the title compound as a colorless powder (742 mg, 62%). ^1^H NMR (300 MHz, DMSO-*d*_6_) δ 7.43–7.34 (m, 4H), 7.20–7.09 (m, 2H), 7.08–6.99 (m, 1H), 6.97–6.88 (m, 2H), 6.07 (s, 2H), 3.76 (t, *J* = 6.5, 2H), 2.82 (p, *J* = 6.3, 2H). ^11^B NMR (96 MHz, DMSO-*d*_6_) δ 4.27. ^13^C NMR (75 MHz, DMSO-*d*_6_) δ 133.20, 133.11, 131.46, 126.66, 124.98, 113.23, 112.98, 62.43, 41.35. ^19^F NMR (282 MHz, DMSO-*d*_6_) δ -118.69 (p, *J* = 9.1). MS (ESI+) *m/z* 305.18 [M+C_2_H_8_NO]^+^, 266.11 [M+Na]^+^, 244.13 [M+H]^+^, 166.08 [M-C_6_H_5_]^+^, 148.09 [M-C_6_H_4_F]^+^. HRMS (ESI+) *m/z* calc. for C_14_H_16_ONBF [M+H]^+^: 244.1303; found 244.1303.

#### 3.1.4. 2-(3-Fluorophenyl)-2-phenyl-1,3,2λ^4^-oxazaborolidine (***m*-F-2APB**)

According to general procedure A, 1-bromo-3-fluorobenzene (392 µL, 5 mmol) in dry THF was lithiated with t-BuLi (6 mL, 1.7 M in pentane, 10.2 mmol) and condensed with phenylboronic acid pinacol ester (1.02 g, 5 mmol). After aqueous work up, the crude borinic acid was purified by flash chromatography and gave a pale yellow oil (188 mg). This was dissolved in abs. EtOH and treated with 2-aminoethanol (70 µL, 1.16 mmol). After solvent removal and drying the residual oil in a Kugelrohr apparatus under high vacuum at 80 °C, the title compound was obtained as off-white powder (223 mg, 17%). ^1^H NMR (300 MHz, DMSO-*d*_6_) δ 7.43–7.35 (m, 2H), 7.23–6.99 (m, 6H), 6.86–6.75 (m, 1H), 6.13 (s, 2H), 3.76 (t, *J* = 6.5, 2H), 2.83 (p, *J* = 6.2, 2H). ^11^B NMR (96 MHz, DMSO-*d*_6_) δ 4.01. ^13^C NMR (75 MHz, DMSO-*d*_6_) δ 163.59, 160.38, 131.41, 128.45, 128.36, 127.32, 127.30, 126.70, 125.08, 117.32, 117.10, 111.52, 111.25, 62.44, 41.35. ^19^F NMR (282 MHz, DMSO-*d*_6_) δ -115.64 (m). MS (ESI+) *m/z* 244.13 [M+H]^+^, 266.11 [M+Na]^+^, 166.08 [M-C_6_H_5_]^+^. HRMS (ESI+) *m/z* calc. for C_14_H_16_ONBF [M+H]^+^: 244.1303; found 244.1300.

#### 3.1.5. 2-(2-Fluorophenyl)-2-phenyl-1,3,2λ^4^-oxazaborolidine (***o*-F-2APB**)

1-Bromo-2-fluorobenzene (547 µL, 5 mmol) was lithiated in dry THF using *t*-BuLi, following general procedure A, and reacted with phenylboronic acid pinacol ester (1.02 g, 5 mmol). After work up and purification of the crude borinic acid by flash chromatography an almost colorless oil was isolated (841 mg) that was mixed with abs. EtOH and 2-aminoethanol (275 µL, 4.56 mmol) to generate the borinate. Drying the crude product in the Kugelrohr under high vacuum at 80 °C gave a slightly brown solid (997 mg), which was recrystallized from MeCN (7 mL), yielding off-white crystals (837 mg, 65%) suitable for X-ray structure determination (Figure S4). ^1^H NMR (300 MHz, DMSO-*d*_6_) δ 7.47–7.33 (m, 3H), 7.17–6.84 (m, 6H), 6.33–6.06 (m, 2H), 3.87–3.75 (m, 1H), 3.73–3.61 (m, 1H), 2.95–2.73 (m, 2H). ^11^B NMR (96 MHz, DMSO-*d*_6_) δ 3.70. ^13^C NMR (75 MHz, DMSO-*d*_6_) δ 133.60, 133.43, 131.58, 131.55, 127.26, 127.15, 126.57, 125.13, 123.15, 123.12, 114.00, 113.66, 62.36, 41.69. ^19^F NMR (376 MHz, DMSO-*d*_6_) δ -105.62 (m). MS (ESI+) *m/z* 244.13 [M+H]^+^, 266.11 [M+Na]^+^, 305.18 [M+C_2_H_8_NO]^+^. HRMS (ESI+) *m/z* calc. for C_14_H_16_ONBF [M+H]^+^: 244.1303; found 244.1305.

#### 3.1.6. 2-(4-Chlorophenyl)-2-phenyl-1,3,2λ^4^-oxazaborolidine (***p*-Cl-2APB**)

Following general procedure A, the title compound was synthesized from 1-chloro-4-iodobenzene (1.1 g, 4.61 mmol) using dry Et_2_O as reaction solvent. Work up and purification by flash chromatography yielded a colorless solid (620 mg) which was treated with 2-aminoethanol (200 µL, 3.32 mmol) in MeCN. The mixture was heated to reflux for 1 h in order to dissolve all the solid. Removing the solvent under reduced pressure afforded a foam, which was dried in vacuo. The solidified foam was crushed and washed with *n*-hexane. Further drying under high vacuum gave the title compound as a colorless powder (743 mg, 62%). ^1^H NMR (300 MHz, DMSO-*d*_6_) δ 7.43–7.33 (m, 4H), 7.20–7.10 (m, 4H), 7.08–7.00 (m, 1H), 6.11 (s, 2H), 3.75 (t, *J* = 6.5, 2H), 2.82 (p, *J* = 6.2, 2H). ^11^B NMR (96 MHz, DMSO-*d*_6_) δ 4.14. ^13^C NMR (75 MHz, DMSO-*d*_6_) δ 133.38, 131.43, 129.85, 126.69, 126.44, 125.05, 62.45, 41.36. MS (ESI+) *m/z* 260.10 [M+H]^+^, 282.08 [M+Na]^+^, 300.13 [M+C_2_H_3_N]^+^, 321.15 [M+C_2_H_8_NO]^+^, 182.05 [M-C_6_H_5_]^+^, 148.09 [M-C_6_H_4_Cl]^+^. HRMS (ESI+) *m/z* calc. for C_14_H_16_ONBCl [M+H]^+^: 260.1008; found 260.1011.

#### 3.1.7. 2-(3-Chlorophenyl)-2-phenyl-1,3,2λ^4^-oxazaborolidine (***m*-Cl-2APB**)

1-Bromo-3-chlorobenzene (587 µL, 5 mmol) was lithiated in dry THF and reacted with phenylboronic acid pinacol ester according to general procedure A. Work up and purification by flash chromatography gave a pale yellow oil (741 mg) that was treated with 2-aminoethanol in abs. EtOH for 1 h at r.t. After solvent removal and drying of the residual oil in a Kugelrohr under high vacuum at 80 °C the title compounds was isolated as a colorless powder (746 mg, 58%). ^1^H NMR (300 MHz, DMSO-*d*_6_) δ 7.42–7.29 (m, 4H), 7.20–7.10 (m, 3H), 7.10–7.01 (m, 2H), 6.16 (s, 2H), 3.76 (t, *J* = 6.5, 2H), 2.83 (p, *J* = 6.2, 2H). ^11^B NMR (96 MHz, DMSO-*d*_6_) δ 4.17. ^13^C NMR (75 MHz, DMSO-*d*_6_) δ 132.07, 131.37, 130.99, 129.93, 128.62, 126.74, 125.12, 124.72, 62.45, 41.37. MS (ESI+) *m/z* 260.10 [M+H]^+^, 282.08 [M+Na]^+^, 321.15 [M+C_2_H_8_NO]^+^, 182.05 [M-C_6_H_5_]^+^, 148.09 [M-C_6_H_4_Cl]^+^. HRMS (ESI+) *m/z* calc. for C_14_H_16_ONBCl [M+H]^+^: 260.1008; found 260.1006.

#### 3.1.8. 2-(2-Chlorophenyl)-2-phenyl-1,3,2λ^4^-oxazaborolidine (***o*-Cl-2APB**)

The title compound was synthesized from 1-bromo-2-chlorobenzene (584 µL, 5 mmol) following general procedure A using THF as the reaction solvent. Work up and flash chromatography purification of the borinic acid afforded a colorless oil (450 mg) that was subjected to 2-aminoethanol treatment in abs. EtOH. After removing the solvent and drying of the oily product (Kugelrohr, 80 °C, high vacuum) a colorless solid was isolated, which was suspended in Et_2_O (10 mL) using an ultrasonic bath. After the precipitate settled, the supernatant was removed. Washing with Et_2_O was repeated two more times and the remaining solid (490 mg) was recrystallized from MeCN (3 mL), yielding colorless crystals (235 mg, 18%) suitable for X-ray diffraction analysis (Figure S5). ^1^H NMR (300 MHz, DMSO-*d*_6_) δ 7.70–7.59 (m, 1H), 7.37–7.27 (m, 2H), 7.24–6.93 (m, 6H), 6.39 (s, 1H), 6.14 (s, 1H), 3.84–3.68 (m, 1H), 3.68–3.57 (m, 1H), 2.88 (dd, *J* = 15.4, 8.2, 2H). ^11^B NMR (96 MHz, DMSO-*d*_6_) δ 4.38. ^13^C NMR (75 MHz, DMSO-*d*_6_) δ 137.62, 133.77, 131.94, 131.52, 128.20, 127.27, 126.34, 125.55, 125.00, 62.09, 42.25. MS (ESI+) *m/z* 260.10 [M+H]^+^, 282.08 [M+Na]^+^, 336.13 [M+C_6_H_5_]^+^, 182.05 [M-C_6_H_5_]^+^. HRMS (ESI+) *m/z* calc. for C_14_H_16_ONBCl [M+H]^+^: 260.1008; found 260.1005.

#### 3.1.9. 2-(4-Bromophenyl)-2-phenyl-1,3,2λ^4^-oxazaborolidine (***p*-Br-2APB**)

The starting material 1,4-dibromobenzene (1 g, 4.24 mmol) was purified by flash chromatography (silica gel, cyclohexane/EtOAc gradient) prior to use in the reaction. General procedure A was followed using dry Et_2_O as the reaction solvent and *n*-BuLi solution (2.5 M in hexanes) for the lithiation. The thus obtained pale yellow, oily borinic acid (502 mg) after work up and flash chromatography was transformed into its borinate by 2-aminoethanol treatment in abs. EtOH. The oily product was dried (Kugelrohr, 80 °C, high vacuum), which yielded the title compound as colorless powder (472 mg, 37%). ^1^H NMR (300 MHz, DMSO-*d*_6_) δ 7.39–7.27 (m, 6H), 7.17–7.09 (m, 2H), 7.08–7.00 (m, 1H), 6.11 (s, 2H), 3.75 (t, *J* = 6.5, 2H), 2.82 (t, *J* = 6.3, 2H). ^11^B NMR (96 MHz, DMSO-*d*_6_) δ 4.11. ^13^C NMR (75 MHz, DMSO-*d*_6_) δ 133.83, 131.41, 129.33, 126.69, 125.05, 118.56, 62.44, 41.36. MS (ESI+) *m/z* 304.05 [M+H]^+^, 326.03 [M+Na]^+^, 226.00 [M-C_6_H_5_]^+^, 148.09 [M-C_6_H_4_Br]^+^. HRMS (ESI+) *m/z* calc. for C_14_H_16_ONBBr [M+H]^+^: 304.0503; found 304.0500.

#### 3.1.10. 2-(3-Bromophenyl)-2-phenyl-1,3,2λ^4^-oxazaborolidine (***m*-Br-2APB**)

Following general procedure A, the title compound was synthesized from 1,3-dibromobenzene (550 µL, 4.55 mmol) using dry Et_2_O as reaction solvent. Work up and purification by flash chromatography gave the borinic acid as a yellow oil (1.06 g), which was dissolved in abs. EtOH and esterified with 2-aminoethanol. Solvent removal and drying the oily product in the Kugelrohr at 80 °C in high vacuum yielded the final borinate as a colorless powder (887 mg, 64%). ^1^H NMR (400 MHz, DMSO-*d*_6_) δ 7.54–7.49 (m, 1H), 7.39–7.35 (m, 3H), 7.24–7.19 (m, 1H), 7.17–7.02 (m, 4H), 6.17 (s, 2H), 3.76 (t, *J* = 6.5, 2H), 2.88–2.78 (m, 2H). ^11^B NMR (96 MHz, DMSO-*d*_6_) δ 3.84. ^13^C NMR (75 MHz, DMSO-*d*_6_) δ 133.91, 131.37, 130.30, 129.07, 127.61, 126.75, 125.14, 121.46, 62.45, 41.38. MS (ESI+) m/z 304.05 [M+H]^+^, 326.03 [M+Na]^+^, 365.10 [M+C_2_H_8_NO]^+^. HRMS (ESI+) *m/z* calc. for C_14_H_16_ONBBr [M+H]^+^: 304.0503; found 304.0504.

#### 3.1.11. 2-(2-Bromophenyl)-2-phenyl-1,3,2λ^4^-oxazaborolidine (***o*-Br-2APB**)

A mixture of THF (10 mL), toluene (40 mL), 1,2-dibromobenzene (602 μL, 5 mmol), and phenylboronic acid pinacol ester (2.04 g, 10 mmol) was cooled down to −78 °C. *n*-BuLi solution (2 mL, 2.5 M in hexanes, 5 mmol) was added dropwise over a period of 3 h. The cloudy white mixture was allowed to warm up to r.t. overnight. The mixture was quenched by the addition of aq. 1 M HCl (50 mL) and EtOAc (100 mL) was added. The resulting two phases were separated and the organic phase was washed with aq. 1 M HCl (3 × 50 mL). The organic phase was dried over Na_2_SO_4_ and the solvents were removed under reduced pressure. The residue was dried in vacuo, yielding a viscous sticky solid. The solid was purified by flash chromatography (silica gel, with cyclohexane/EtOAc gradient) affording an oil (446 mg). The oil was dissolved in abs. EtOH (5 mL) and 2-aminoethanol (120 μL, 2.00 mmol) was added to the solution. The resulting mixture was stirred at r.t. for 1 h. The solvent was removed under reduced pressure and the residue was dried in the Kugelrohr under high vacuum at 80 °C. The resulting solid was recrystallized from MeCN yielding the title compound as colorless crystals (189 mg, 12%). ^1^H NMR (300 MHz, DMSO-*d*_6_) δ 7.68 (dd, *J* = 7.4, 1.9, 1H), 7.38–7.27 (m, 3H), 7.22 (td, *J* = 7.3, 1.2, 1H), 7.12–6.97 (m, 4H), 6.40 (s, 1H), 6.12 (s, 1H), 3.80–3.69 (m, 1H), 3.66–3.56 (m, 1H), 3.02–2.80 (m, 2H). ^11^B NMR (96 MHz, DMSO-*d*_6_) δ 4.53. ^13^C NMR (75 MHz, DMSO-*d*_6_) δ 134.25, 132.14, 131.61, 128.57, 127.64, 126.28, 126.00, 124.96, 62.10, 42.51. MS (ESI+) *m/z* 304.05 [M+H]^+^. HRMS (ESI+) *m/z* calc. for C_14_H_16_ONBBr [M+H]^+^: 304.0503; found 304.0496.

#### 3.1.12. 2-(3-Iodophenyl)-2-phenyl-1,3,2λ^4^-oxazaborolidine (***m*-I-2APB**)

1,3-Diiodobenzene (1.65 g, 5 mmol) was lithiated with *n*-BuLi (2 mL, 2.5 M in hexanes, 5 mmol) in dry THF, following general procedure A, and reacted with phenylboronic acid pinacol ester to yield the corresponding borinic acid (604 mg) after work up and flash chromatography purification as a pale yellow oil. Treatment with 2-aminoethanol in abs. EtOH yielded, after solvent removal and drying (Kugelrohr, 80 °C, high vacuum), a sticky solid. This was reprecipitated with MeCN, filtered and dried again in the Kugelrohr (80 °C, high vacuum). The title compound was thus obtained as a colorless powder (434 mg, 25%). ^1^H NMR (300 MHz, DMSO-*d*_6_) δ 7.76–7.69 (m, 1H), 7.44–7.31 (m, 4H), 7.20–6.92 (m, 4H), 6.15 (s, 2H), 3.75 (t, *J* = 6.5, 2H), 2.82 (p, *J* = 6.2, 4H). ^11^B NMR (96 MHz, DMSO-*d*_6_) δ 3.74. ^13^C NMR (75 MHz, DMSO-*d*_6_) δ 139.99, 133.49, 131.36, 130.71, 129.30, 126.74, 125.12, 95.30, 62.44, 41.38. MS (ESI+) *m/z* 352.04 [M+H]^+^, 374.02 [M+Na]^+^, 413.09 [M+C_2_H_8_NO]^+^, 273.99 [M-C_6_H_5_]^+^, 148.09 [M-C_6_H_4_I]^+^. HRMS (ESI+) *m/z* calc. for C_14_H_16_ONBI [M+H]^+^: 352.0364; found 352.0355.

#### 3.1.13. 4-(2-Phenyl-1,3,2λ^4^-oxazaborolidin-2-yl)benzonitrile (***p*-CN-2APB**)

Following general procedure A, the title compound was synthesized from 4-bromobenzonitrile (900 mg, 4.95 mmol) using dry THF as reaction solvent. Work up and purification by flash chromatography yielded the borinic acid (712 mg) as a beige powder. It was dissolved in MeCN and treated with 2-aminoethanol, which gave the borinate as a sticky solid after solvent removal. This was redissolved in CH_2_Cl_2_, the solvent evaporated and the residual solid was dried under high vacuum. This afforded the title compound as a colorless powder (816 mg, 66%). ^1^H NMR (300 MHz, DMSO-*d*_6_) δ 7.66–7.51 (m, 4H), 7.41–7.33 (m, 2H), 7.19–7.08 (m, 2H), 7.09–7.00 (m, 1H), 6.26 (s, 2H), 3.88–3.68 (m, 2H), 2.94–2.76 (m, 2H). ^11^B NMR (96 MHz, DMSO-*d*_6_) δ 3.94. ^13^C NMR (75 MHz, DMSO-*d*_6_) δ 132.19, 131.34, 130.14, 126.81, 125.25, 119.89, 107.53, 62.48, 41.40, 39.52. MS (ESI+) *m/z* 251.13 [M+H]^+^, 273.12 [M+Na]^+^, 312.19 [M+C_2_H_8_NO]^+^, 173.09 [M-C_6_H_5_]^+^. HRMS (ESI+) *m/z* calc. for C_15_H_16_ON_2_B [M+H]^+^: 251.1350; found 251.1349.

#### 3.1.14. 2-(4-Nitrophenyl)-2-phenyl-1,3,2λ^4^-oxazaborolidine (***p*-NO_2_-2APB**)

A solution of 4-nitrophenylboronic acid pinacol ester (500 mg, 2.01 mmol) in dry THF (20 mL) was cooled to −78 °C. Phenyllithium solution (1.1 mL, 1.8 M in Bu_2_O, 1.98 mmol) was added dropwise to the mixture. During the addition the drop rate was adjusted so that the temperature of the mixture remained below −75 °C. The mixture was stirred at −78 °C for 4 h. While still stirring at −78 °C, the mixture was quenched by the addition of aq. 1 M HCl (10 mL) over a period of 2 min. More aq. 1 M HCl (40 mL) and CH_2_Cl_2_ (100 mL) were added to the mixture. The resulting two phases were separated. The clear yellow organic phase was dried over Na_2_SO_4_ and the solvent was removed under reduced pressure. The remaining brown oil was purified by flash chromatography (silica gel, gradient with CH_2_Cl_2_ and MeOH/CH_2_Cl_2_ 1:19, (*v*/*v*)) affording a black oil (146 mg). The oil was dissolved in abs. EtOH (4 mL) and 2-aminoethanol (40 μL, 0.66 mmol) were added to the solution. The resulting mixture was stirred at r.t. for 30 min. The solvent was removed under reduced pressure and the residue was dried in vacuo affording a sticky yellow solid with a slight greenish tint. The solid was suspended in Et_2_O (20 mL) using an ultrasonic bath. After the solid settled, the supernatant was removed. This washing step was repeated once. The residue was dried in the Kugelrohr at 80 °C under high vacuum affording the title compound as a beige powder (47 mg, 9%). ^1^H NMR (300 MHz, DMSO-*d*_6_) δ 8.06–7.96 (m, 2H), 7.71–7.62 (m, 2H), 7.45–7.34 (m, 2H), 7.20–7.11 (m, 2H), 7.11–7.01 (m, 1H), 6.30 (s, 2H), 3.87–3.69 (m, 2H), 2.92–2.79 (m, 1H). ^11^B NMR (96 MHz, DMSO-*d*_6_) δ 4.25. ^13^C NMR (75 MHz, DMSO-*d*_6_) δ 145.69, 132.35, 131.38, 126.86, 125.34, 121.39, 117.03, 62.53, 41.47. MS (ESI+) *m/z* 271.12 [M+H]^+^, 293.11 [M+Na]^+^, 311.16 [M+C_2_H_3_N]^+^, 332.18 [M+C_2_H_8_NO]^+^, 193.08 [M-C_6_H_5_]^+^. HRMS (ESI+) *m/z* calc. for C_14_H_16_O_3_N_2_B [M+H]^+^: 271.1248; found 271.1249.

#### 3.1.15. 2-Phenyl-2-(4-(trimethylsilyl)phenyl)-1,3,2λ^4^-oxazaborolidine (***p*-TMS-2APB**)

1-Bromo-4-(trimethylsilyl)benzene (400 µL, 2.05 mmol) was reacted according to general procedure A in dry Et_2_O as reaction solvent. After work up and flash chromatography purification the borinic acid was obtained as pale yellow oil (170 mg), which was subjected to 2-aminoethanol treatment in abs. EtOH. The solid, isolated after solvent removal, was suspended in Et_2_O (10 mL) using an ultrasonic bath. After the solid settled, the supernatant was removed. This washing procedure was repeated two times with Et_2_O (10 mL) and once with *n*-hexane (5 mL). After drying the solid residue in high vacuum the title compound was isolated as colorless powder (49 mg, 8%). ^1^H NMR (300 MHz, DMSO-*d*_6_) δ 7.44–7.35 (m, 4H), 7.33–7.24 (m, 2H), 7.17–7.07 (m, 2H), 7.06–6.98 (m, 1H), 6.06 (s, 2H), 3.75 (t, *J* = 6.5, 2H), 2.81 (p, *J* = 6.4, 2H), 0.18 (s, 9H). ^11^B NMR (96 MHz, DMSO-*d*_6_) δ 4.42. ^13^C NMR (75 MHz, DMSO-*d*_6_) δ 134.96, 131.55, 131.44, 131.01, 126.58, 124.87, 62.41, 41.37, -0.92. MS (ESI+) *m/z* 298.18 [M+H]^+^, 320.16 [M+Na]^+^, 359.23 [M+C_2_H_8_NO]^+^, 220.13 [M-C_6_H_5_]^+^. HRMS (ESI+) *m/z* calc. for C_17_H_25_ONBSi [M+H]^+^: 298.1793; found 298.1793.

#### 3.1.16. 2-(4-Methoxyphenyl)-2-phenyl-1,3,2λ^4^-oxazaborolidine (***p*-OMe-2APB**)

Starting from 4-bromoanisole (669 µL, 5.35 mmol) the title compound was synthesized according to general procedure A. The borinic acid (763 mg), obtained as pale yellow oil after work up and flash chromatography purification, was treated with 2-aminoethanol in MeCN at r.t. Removal of solvent gave a residual sticky solid that was redissolved in CH_2_Cl_2_. The solvent was evaporated under reduced pressure yielding the borinate as a colorless powder (825 mg, 60%). ^1^H NMR (300 MHz, DMSO-*d*_6_) δ 7.41–7.33 (m, 2H), 7.31–7.24 (m, 2H), 7.17–7.08 (m, 2H), 7.06–6.98 (m, 1H), 6.74–6.68 (m, 2H), 5.96 (s, 2H), 3.75 (t, *J* = 6.3, 2H), 2.81 (p, *J* = 6.3, 2H). ^11^B NMR (96 MHz, DMSO-*d*_6_) δ 4.40. ^13^C NMR (75 MHz, DMSO-*d*_6_) δ 157.34, 132.63, 131.56, 126.58, 124.83, 112.34, 62.42, 54.65, 41.33, 39.52. MS (ESI+) *m/z* 256.15 [M+H]^+^, 278.13 [M+Na]^+^, 178.10 [M-C_6_H_5_]^+^, 148.09 [M-C_7_H_5_O]^+^. HRMS (ESI+) *m/z* calc. for C_15_H_19_O_2_NB [M+H]^+^: 256.1503; found 256.1500.

#### 3.1.17. *N*,*N*-Dimethyl-4-(2-phenyl-1,3,2λ^4^-oxazaborolidin-2-yl)benzenesulfonamide (***p*-SO_2_NMe_2_-2APB**)

4-Bromo-*N*,*N*-diemthylbenzenesulfonamide (1 g, 3.79 mmol), which was freshly purified by flash chromatography (silica gel, cyclohexane/EtOAc gradient), was used as starting material. Dry THF was used as the reaction solvent and *n*-BuLi solution (2.5 M in hexanes) for the lithiation to synthesize the corresponding borinic acid according to general procedure A. The borinic acid was obtained as brown oil (930 mg) after work up and flash chromatography purification (silica gel, gradient with CH_2_Cl_2_ and MeOH/CH_2_Cl_2_ 1:19 (*v*/*v*)). The oil was dissolved in abs. EtOH and 2-aminoethanol added to the solution. Removal of solvent yielded a sticky solid that was dried (Kugelrohr, 80 °C, high vacuum), affording an off-white solid. Recrystallization from MeCN gave the title compound (630 mg, 52%) as off-white crystals, suitable for X-ray diffraction analysis (Figure S3). ^1^H NMR (300 MHz, DMSO-*d*_6_) δ 7.68–7.63 (m, 2H), 7.53–7.49 (m, 2H), 7.43–7.37 (m, 2H), 7.20–7.12 (m, 2H), 7.09–7.02 (m, 1H), 6.24 (s, 2H), 3.78 (t, *J* = 6.5, 2H), 2.84 (p, *J* = 6.1, 2H), 2.55 (s, 6H). ^11^B NMR (96 MHz, DMSO-*d*_6_) δ 4.17. ^13^C NMR (75 MHz, DMSO-*d*_6_) δ 131.96, 131.36, 126.82, 125.76, 125.21, 117.00, 62.51, 41.44, 37.59. MS (ESI+) *m/z* 333.14 [M+H]^+^, 355.13 [M+Na]^+^, 394.20 [M+C_2_H_8_NO]^+^, 255.10 [M-C_6_H_5_]^+^; HRMS (ESI+) *m/z* calc. for C_16_H_22_O_3_N_2_BS [M+H]^+^: 333.1439; found 333.1433.

#### 3.1.18. 6,6-Diphenyl-7-oxa-5-aza-6λ^4^-boraspiro[3.4]octan-8-one (**cBu-2APB**)

Following general procedure B (reflux for 55 min), 1-amino-1-cyclobutanecarboxylic acid (80 mg, 0.70 mmol) was used as starting material. The crude precipitate was washed with Et_2_O and *n*-hexane, reprecipitated in a mixture of EtOH and *n*-hexane and washed several times with EtOH and *n*-hexane. Drying the solid product in high vacuum afforded the title compound (32 mg, 16%), containing crystals suitable for X-ray structure determination (Figure S2). ^1^H NMR (300 MHz, DMSO-*d*_6_) δ 7.43–7.29 (m, 6H), 7.24–7.14 (m, 4H), 7.14–7.06 (m, 2H), 2.20–2.05 (m, 4H), 1.99–1.75 (m, 2H). ^11^B NMR (96 MHz, DMSO-*d*_6_) δ 4.27. ^13^C NMR (75 MHz, DMSO-*d*_6_) δ 176.39, 130.69, 126.97, 125.79, 59.16, 30.71, 14.72. MS (ESI+) *m/z* 280.15 [M+H]^+^, 302.13 [M+Na]^+^. HRMS (ESI+) *m/z* calc. for C_17_H_19_O_2_NB [M+H]^+^: 280.1503; found 280.1502.

#### 3.1.19. 2,2-Diphenyl-1,3,2λ^4^-oxazaborinan-6-one (**βAla-2APB**)

β-Alanine (60 mg, 0.67 mmol) was reacted as described in general procedure B (reflux for 1.5 h). The product crystallized from the reaction solution upon cooling. The colorless crystals were washed with *n*-hexane and dried in high vacuum. This gave the title compound as colorless crystals (88 mg, 52%). ^1^H NMR (300 MHz, DMSO-*d*_6_) δ 7.41 (dd, *J* = 8.0, 1.3, 4H), 7.25–7.16 (m, 4H), 7.14–7.05 (m, 2H), 6.76 (s, 2H), 2.90–2.80 (m, 2H), 2.54–2.41 (m, 2H). ^11^B NMR (96 MHz, DMSO-*d*_6_) δ 2.05. ^13^C NMR (75 MHz, DMSO-*d*_6_) δ 169.23, 130.78, 127.02, 125.61, 35.48, 30.01. MS (ESI+) *m/z* 254.13 [M+H]^+^, 276.12 [M+Na]^+^, 176.09 [M-C_6_H_5_]^+^. HRMS (ESI+) *m/z* calc. for C_15_H_17_O_2_NB [M+H]^+^: 254.1347; found 254.1345.

#### 3.1.20. (*S*)-3-(5-Oxo-2,2-diphenyl-1,3,2λ^4^-oxazaborolidin-4-yl)propanamide (**Gln-2APB**)

L-Glutamine (95 mg, 0.65 mmol) was treated as described in general procedure B (reflux for 3.75 h) and the product precipitated from the reaction mixture upon cooling. The solid was washed with EtOH, Et_2_O, and *n*-hexane and dried in high vacuum. The residue was recrystallized from EtOH affording the title compound as colorless crystals (96 mg, 48%). ^1^H NMR (300 MHz, DMSO-*d*_6_) δ 7.43–7.28 (m, 6H), 7.21 (td, J = 7.1, 1.6, 4H), 7.18–7.07 (m, 2H), 7.03–6.90 (m, 1H), 6.86 (s, 1H), 3.56 (p, J = 7.5, 1H), 2.30 (td, J = 7.2, 2.7, 2H), 1.89 (ddt, J = 39.3, 14.7, 7.4, 2H). ^11^B NMR (96 MHz, DMSO-*d*_6_) δ 4.39. ^13^C NMR (75 MHz, DMSO-*d*_6_) δ 174.07, 173.86, 131.00, 130.93, 127.08, 127.03, 126.01, 125.91, 54.44, 30.88, 25.31. MS (ESI+) *m/z* 311.16 [M+H]^+^, 333.14 [M+Na]^+^. HRMS (ESI+) *m/z* calc. for C_17_H_20_O_3_N_2_B [M+H]^+^: 311.1567; found 311.1561.

#### 3.1.21. (*S*)-4-((1*H*-Imidazol-5-yl)methyl)-2,2-diphenyl-1,3,2λ^4^-oxazaborolidin-5-one (**His-2APB**)

L-Histidine (100 mg, 0.65 mmol) was used as starting material and general procedure B followed to synthesize the title compound (reflux for 1 h). The crude product precipitated from the reaction mixture upon cooling. It was washed with Et_2_O and *n*-hexane and dried in high vacuum. This gave the title compound as colorless solid (92 mg, 45%). ^1^H NMR (300 MHz, DMSO-*d*_6_) δ 12.04 (s, 1H), 7.62–7.54 (m, 1H), 7.48–7.07 (m, 12H), 6.91 (s, 1H), 3.84 (t, *J* = 7.2, 1H), 2.95 (d, *J* = 6.0, 2H). ^11^B NMR (96 MHz, DMSO-*d*_6_) δ 4.40. ^13^C NMR (75 MHz, DMSO-*d*_6_) δ 173.32, 135.19, 131.18, 131.07, 127.04, 127.01, 126.01, 55.18. MS (ESI+) *m/z* 320.16 [M+H]^+^, 342.14 [M+Na]^+^, 242.11 [M-C_6_H_5_]^+^. HRMS (ESI+) *m/z* calc. for C_18_H_19_O_2_N_3_B [M+H]^+^: 320.1565; found 320.1562.

#### 3.1.22. (*S*)-4-(2-(Methylthio)ethyl)-2,2-diphenyl-1,3,2λ^4^-oxazaborolidin-5-one (**Met-2APB**)

Following general procedure B, L-methionine (100 mg, 0.67 mmol) was used as the starting material. After refluxing for 45 min the clear solution was cooled to r.t. and then stored at −20 °C to initiate precipitation of the crude product. The obtained crystals were washed with Et_2_O and *n*-hexane, and dried in high vacuum. This afforded the desired product as colorless crystals (212 mg, quantitative). ^1^H NMR (300 MHz, DMSO-*d*_6_) δ 7.45–7.09 (m, 12H), 6.78 (t, *J* = 10.4, 1H), 3.69–3.53 (m, 1H), 2.72–2.52 (m, 2H), 2.14–1.78 (m, 4H). ^11^B NMR (96 MHz, DMSO-*d*_6_) δ 4.92. ^13^C NMR (75 MHz, DMSO-*d*_6_) δ 174.04, 130.99, 130.92, 127.14, 127.08, 126.08, 125.98, 53.60, 29.51, 28.95, 14.15. MS (ESI+) *m/z* 314.14 [M+H]^+^, 336.12 [M+Na]^+^, 236.09 [M-C_6_H_5_]^+^. HRMS (ESI+) *m/z* calc. for C_17_H_21_O_2_NBS [M+H]^+^: 314.1381; found 314.1380.

#### 3.1.23. (*R*)-4-(Mercaptomethyl)-2,2-diphenyl-1,3,2λ^4^-oxazaborolidin-5-one (**Cys-2APB**)

L-Cysteine (75 mg, 0.62 mmol) was reacted according to general procedure B (reflux for 1 h). The crude product, which precipitated from the reaction mixture upon cooling, was washed with *n*-hexane. This gave the title compound as colorless solid (125 mg, 71%) after drying in high vacuum. ^1^H NMR (300 MHz, DMSO-*d*_6_) δ 7.58–7.36 (m, 5H), 7.29–7.09 (m, 6H), 6.63 (t, *J* = 10.8, 1H), 3.79–3.63 (m, 1H), 3.04–2.65 (m, 3H). ^11^B NMR (96 MHz, DMSO-*d*_6_) δ 4.58. ^13^C NMR (75 MHz, DMSO-*d*_6_) δ 172.29, 131.14, 131.08, 127.08, 127.06, 126.12, 126.07, 57.81, 23.83. MS (ESI+) *m/z* 286.11 [M+H]^+^, 308.09 [M+Na]^+^. HRMS (ESI+) *m/z* calc. for C_15_H_17_O_2_NBS [M+H]^+^: 286.1068; found 286.1066.

#### 3.1.24. (*S*)-4-Benzyl-2,2-diphenyl-1,3,2λ^4^-oxazaborolidin-5-one (**Phe-2APB**)

The title compound was synthesized from L-phenylalanine (100 mg, 0.60 mmol) according to general procedure B (reflux for 30 min). The precipitated crude product was washed with *n*-hexane and dried in high vacuum. This yielded the final compound as colorless solid (128 mg, 64%). ^1^H NMR (300 MHz, DMSO-*d*_6_) δ 7.47–7.01 (m, 16H), 6.76 (t, *J* = 10.5, 1H), 3.77–3.61 (m, 1H), 3.17 (dd, *J* = 14.5, 4.0, 1H), 2.92 (dd, *J* = 14.5, 10.3, 1H). ^11^B NMR (96 MHz, DMSO-*d*_6_) δ 4.61. ^13^C NMR (75 MHz, DMSO-*d*_6_) δ 173.48, 137.26, 131.15, 130.81, 129.27, 128.47, 127.02, 126.95, 126.65, 125.95, 125.86, 56.68, 35.04. MS (ESI+) *m/z* 330.17 [M+H]^+^, 352.15 [M+Na]^+^. HRMS (ESI+) *m/z* calc. for C_21_H_21_O_2_NB [M+H]^+^: 330.1660; found 330.1658.

#### 3.1.25. (*S*)-4-(4-Hydroxybenzyl)-2,2-diphenyl-1,3,2λ^4^-oxazaborolidin-5-one (**Tyr-2APB**)

Following general procedure B, L-tyrosine (120 mg, 0.68 mmol) was reacted (reflux for 40 min) and the reaction mixture cooled to r.t. and then −20 °C. The formed precipitate was washed with Et_2_O and *n*-hexane. The isolated solid was suspended in EtOH, layered with *n*-hexane, and the mixture stored at 5 °C. The obtained precipitate was filtered, washed with *n*-hexane, and dried in high vacuum. This gave the title compound as shimmery beige solid (39 mg, 17%). ^1^H NMR (300 MHz, DMSO-*d*_6_) δ 9.26 (s, 1H), 7.39 (td, *J* = 7.9, 1.6, 4H), 7.30–6.99 (m, 9H), 6.74–6.55 (m, 3H), 3.64–3.53 (m, 1H), 3.04 (dd, *J* = 14.5, 4.0, 1H), 2.79 (dd, *J* = 14.5, 10.3, 1H). ^11^B NMR (96 MHz, DMSO-*d*_6_) δ 4.73. ^13^C NMR (75 MHz, DMSO-*d*_6_) δ 173.56, 156.18, 131.15, 130.81, 130.22, 127.10, 127.00, 126.94, 125.93, 125.85, 115.26, 57.04, 34.18. MS (ESI+) *m/z* 346.16 [M+H]^+^, 368.14 [M+Na]^+^. HRMS (ESI+) *m/z* calc. for C_21_H_21_O_3_NB [M+H]^+^: 346.1609; found 346.1605.

#### 3.1.26. (*S*)-4-((5-Hydroxy-1*H*-indol-3-yl)methyl)-2,2-diphenyl-1,3,2λ^4^-oxazaborolidin-5-one (**5-HO-Trp-2APB**)

5-Hydroxytryptophan (191 mg, 0.87 mmol) was used as the starting material to synthesize the title compound according to general procedure B (reflux until all the solids were dissolved). Precipitation of the crude product was initiated by first cooling to r.t. and then to −20 °C. The precipitate was washed with Et_2_O and *n*-hexane and the obtained solid recrystallized from EtOH. This gave the desired compound as a colorless solid (220 mg, 66%) after drying in high vacuum. ^1^H NMR (300 MHz, DMSO-*d*_6_) δ 10.67–10.58 (m, 1H), 8.60 (s, 1H), 7.42–7.34 (m, 4H), 7.29 (dd, *J* = 12.0, 6.2, 1H), 7.23–7.04 (m, 7H), 7.03 (d, *J* = 2.3, 1H), 6.75 (d, *J* = 2.2, 1H), 6.59 (dd, *J* = 8.6, 2.2, 1H), 6.52–6.35 (m, 1H), 3.73–3.58 (m, 1H), 3.14 (dd, *J* = 15.1, 3.5, 1H), 2.93 (dd, *J* = 15.2, 10.1, 1H). ^11^B NMR (96 MHz, DMSO-*d*_6_) δ 8.58. ^13^C NMR (75 MHz, DMSO-*d*_6_) δ 174.01, 150.52, 131.19, 130.93, 127.47, 127.12, 127.05, 126.05, 125.99, 124.97, 111.99, 111.58, 107.80, 101.86, 55.71, 25.58. MS (ESI+) *m/z* 385.1726 [M+H]^+^, 407.1543 [M+Na]^+^, 307.1254 [M-C_6_H_5_]^+^. HRMS (ESI+) *m/z* calc. for C_23_H_22_O_3_N_2_B [M+H]^+^: 385.1718; found 385.1726.

#### 3.1.27. (*S*)-4-(4-Aminobutyl)-2,2-diphenyl-1,3,2λ^4^-oxazaborolidin-5-one (**Lys-2APB**)

Following general procedure B, L-lysine (90 mg, 0.62 mmol) was reacted (reflux for 50 min) and the solvents evaporated under reduced pressure. The residue was layered with a mixture of Et_2_O and *n*-hexane, and the precipitation allowed to settle. The supernatant was removed and the solid washed with Et_2_O and *n*-hexane. After drying the solid in high vacuum, it was recrystallized from a mixture of *i*-PrOH and EtOH, affording the title compound as colorless crystals (96 mg, 50%). ^1^H NMR (300 MHz, DMSO-*d*_6_) δ 7.43–7.36 (m, 5H), 7.25–7.17 (m, 5H), 7.16–7.09 (m, 2H), 3.41 (dd, *J* = 9.4, 4.6, 1H), 2.48–2.44 (m, 2H), 1.80–1.21 (m, 8H). ^11^B NMR (96 MHz, DMSO-*d*_6_) δ 4.42. ^13^C NMR (75 MHz, DMSO-*d*_6_) δ 174.27, 131.00, 130.92, 127.05, 126.99, 125.95, 125.86, 55.17, 41.21, 32.74, 29.37, 22.97. MS (ESI+) *m/z* 311.19 [M+H]^+^, 333.17 [M+Na]^+^, 233.15 [M-C_6_H_5_]^+^. HRMS (ESI+) *m/z* calc. for C_18_H_24_O_2_N_2_B [M+H]^+^: 311.1925; found 311.1925.

### 3.2. Cell Culture

Cell culture reagents were purchased from Thermo Fisher Scientific (Waltham, MA, USA). MDA-MB-231 cells (ATCC) were cultured at 37 °C in RPMI cell culture medium supplemented with 10% FBS, 10 mM HEPES, and 1% penicillin/streptomycin. HEK 293 cells stably expressing STIM1 were grown at 37 °C and 5% CO_2_ in MEM medium (Gibco, Thermo Fisher Scientific, Waltham, MA, USA) supplemented with 10% FCS and 500 µg/mL G418 (Gibco). Cells were transfected according to manufacturer’s guidelines in a Nucleofector 4D device with Nucleofector Kit SF (Lonza, Basel, Switzerland) with 1 µg DNA per 10^6^ cells.

### 3.3. Fluorescent Ca^2+^ Influx Assay Using FLIPR^®^ Tetra

#### 3.3.1. General

The calcium indicator, Calcium-5 was purchased from Molecular Devices LLC and all other chemicals were obtained from Sigma-Aldrich (St. Louis, MO, USA). For loading the cells and subsequent administrations during the FLIPR experiments a nominal calcium free (NCF) modified Krebs buffer was used, containing 117 mM NaCl, 4.8 mM KCl, 1 mM MgCl_2_, 5 mM D-glucose, and 10 mM HEPES. The pH of the NCF buffer was adjusted to 7.4 using HCl and NaOH. Thapsigargin (Tg) solutions were always prepared freshly a few minutes before starting the FLIPR experiment. Cells were excited using a 470–495 nm LED module, and the emitted fluorescence signal was filtered with a 515–575 nm emission filter (manufacturer’s guidelines). Fluorescence signals were analyzed using the ScreenWorks 3.1.1.8 software (Molecular Devices).

#### 3.3.2. SOCE Modulation Assay

The attached MDA-MB-231 cells from T75 flasks were trypsinized and plated onto Corning^®^ 96-well black polystyrene clear bottom microplates (CLS3603 Sigma–Aldrich, St. Louis, MO, USA) at a density 60,000 cells/well in a final volume of 100 µL per well. Phenol-red free RPMI medium supplemented with 10% FBS and 10 mM HEPES was used for seeding the cells. After culturing the cells for 24 h, the medium of the confluent cells was removed and the cells were loaded with 50 μL NCF Krebs buffer containing calcium-5 dye (50 μL per mL). The cells then were incubated with the dye in the dark for 1 h at 37 °C. Right after the dye-incubation period, the FLIPR experiment was started. After establishment of stable baselines for 50 s, first ER Ca^2+^ stores were depleted by adding and incubating 50 μL of 2 μM thapsigargin (Tg) in NCF Krebs buffer (1 μM final concentration) for 10 min. Next, 100 μL of 2× of the intended final concentration of the drug of interest (synthetic 2-APB analogue or other SOCE inhibitor) in NCF Krebs buffer was applied and incubated for 10 min. Finally, 50 µL of NCF Krebs buffer containing 10 mM CaCl_2_ (2 mM final concentration) was administered while maintaining the 1× concentration of the drug of interest. The subsequent response was monitored for 5 min after the addition of CaCl_2_. The modulation of the SOCE signal was quantified by calculating the area under the curve (AUC) of the relative change of the fluorescence intensity over time (trace) from the last 5 min of the experiment. The values were then normalized to the DMSO positive control (DMSO response = 100%).

#### 3.3.3. ER Ca^2+^ Mobilization Assay (Compound Followed by Thapsigargin)

The medium of the confluent cells was replaced by 50 μL buffer containing 2 mM CaCl_2_ as well as calcium-5 dye (50 μL per mL). The cells were incubated in the dark for 1 h at 37 °C. After the dye was incubated, the cells were washed twice: first with 250 μL of 500 μM EGTA in NCF Krebs buffer and afterwards with 250 μL NCF Krebs buffer. Finally, the cells were loaded with 50 μL NCF Krebs buffer and subjected to the FLIPR experiment. Total of 50 μL of 2× of the intended concentration of drug of interest in NCF Krebs buffer was applied and incubated for 10 min while recording the Ca^2+^ response. Afterwards 25 μL of 12.5 μM Tg (2.5 µM final concentration) was applied and the subsequent response was monitored for 10 min. The response after each addition step was quantified by calculating the slope of the curve and normalizing it to the slope of the response after Tg administration.

### 3.4. Electrophysiology

Currents from whole cell patch clamp recordings were acquired and filtered at 2.9 kHz with a HEKA EPC-10 amplifier (HEKA Elektronik, Lambrecht (Pfalz), Germany). Currents were corrected for a liquid junction potential of 10 mV, recorded and analyzed with HEKA Patchmaster (v2×53) software and Igor Pro 6.37 (Wavemetrics, Lake Oswego, OR, USA). 50 ms voltage ramps spanning, 150 mV to 150 mV were delivered every 2 s from a holding potential of 0. Before each voltage ramp, capacitive currents were corrected. Currents at −130 mV and 130 mV were extracted, normalized to cell capacitance, and plotted versus time. Bath solution contained (in mM): 120 NaCl, 20 CaCl_2_, 10 TEA-Cl, 10 HEPES, 2 MgCl_2_. Pipette solution contained (in mM): 120 Cs-glutamate, 20 BAPTA, 10 HEPES, 3 MgCl_2_, and 0.050 IP_3_. Osmolarity was adjusted with glucose to 310 mOsm and pH was adjusted to 7.2 with CsOH.

### 3.5. CRISPR/Cas9-Mediated Generation and Validation of STIM1 and Orai1 Knockout MDA-MB-231 Cells

Earlier described methods were used to generate STIM1 knockout [86] and Orai1 knockout MDA-MB-231 cells [87]. Single-cell derived clones D11 and G10 were functionally confirmed to have undergone knockout of STIM1 and Orai1, respectively by using SOCE functional assay on FLIPR Tetra^®^ (Molecular Devices). The knockouts were further confirmed by Western blotting. STIM1 was detected using the polyclonal hSTIM1 antibody raised in guinea pig [88] in a 1:1000 dilution. Peroxidase-conjugated affinipure goat anti-guinea pig secondary antibody was used in a 1:10,000 dilution (106-035-003, Jackson Immuno Research, West Grove, PA, USA). Orai1 was detected using rabbit polyclonal hOrai1 antibody (O8264, Sigma-Aldrich) in a 1:1000 dilution. Horseradish peroxidase (HRP)-conjugated secondary goat anti-rabbit IgG (W4011, Promega, Madison, WI, USA) was used in a 1:20,000 dilution. Tubulin (loading control) was detected using mouse monoclonal Tubulin antibody (T9028, Sigma-Aldrich) in a 1:2000 dilution. HRP-conjugated goat anti-mouse secondary antibody was used in a 1:3000 dilution (172-1011, Bio-Rad, Hercules, CA, USA).

### 3.6. Cell Viability Assay

The XTT assay was used to evaluate the effect of 2-APB analogues on cell proliferation. Cells were plated at a density of 5 × 10^3^ cells/well in 96-well plates and were incubated at 37 °C and 5% CO_2_ for 24 h. On the following day, the medium was carefully aspirated and replaced with 100 μL medium supplemented with DMSO control or different 2-APB compounds and incubated over a range of different time points from 24 to 120 h. At each time point, 25 μL of sodium 3′-[1-[(phenylamino)-carbony]-3,4-tetrazolium]-bis(4-methoxy-6-nitro)benzene-sulfonic acid hydrate (XTT 1mg/mL prepared in Hank’s Balanced Salt Solution) containing 1.25% phenazine methosulphonate (PMS) was added to each well and incubated at 37 °C for 2 h. Subsequently, the absorbance was read at 650 nm and subtracted from the absorbance of 450 nm by spectrophotometry (V_max_ Kinetic Microplate Reader, Molecular Devices LLC). The resulting subtracted absorbance for each compound was expressed as a percentage of viable cells relative to the DMSO control. XTT sodium salt and PMS were purchased from Sigma-Aldrich. The MCF-10A cells were kindly provided by Prof. Dr. Olivier Pertz, Institute of Cell Biology, University of Bern and were cultured in growth medium composed by DMEM/F12 supplemented with 5% horse serum, 20 ng/mL recombinant human EGF (Sigma-Aldrich), 10 μg/mL insulin (Sigma-Aldrich), and 0.5 mg/mL hydrocortisone (Sigma-Aldrich).

### 3.7. Data Analysis

Statistical significance between the two groups was estimated using student’s T-test where both groups were normally distributed and Mann Whitney test in case of data that were not distributed normally. GraphPad Prism 8.4.3 was used to perform the statistical analysis as well as to generate the bar graphs with scattered individual data points. Data are usually shown as mean ± SD.

## 4. Conclusions

In an effort to develop new potent inhibitors of store-operated calcium entry we have synthesized several mono-substituted and amino acid analogues of 2-APB. We tested these 2-APB derivatives using a FLIPR assay format in MDA-MB-231 cells. This breast cancer cell line already has been established by others [26,72] as a viable tool for screening new SOCE inhibitors. The compound series of mono-halogenated 2-APB derivatives, in particular, showed promising inhibitory activity compared to 2-APB. Some members of this series, e.g., ***m*-Cl-2APB**, ***m*-Br-2APB**, ***m*-I-2APB**, and ***p*-I-2APB**, exhibited efficient SOCE block at a five times lower concentration than 2-APB. It is worth noting that at this low concentration 2-APB generally enhances SOCE. Throughout the course of our investigation we have also demonstrated that some of our novel analogues (***m*-I-2APB**, ***p*-Cl-2APB**, ***p*-Br-2APB**, ***p*-I-2APB**), but also known 2-APB-type SOCE inhibitors (**DPB162-AE**), are able to mobilize calcium from intracellular stores of MDA-MB-231 cells at high concentration. In this regard, ***m*-Cl-2APB** is a promising tool compound from our synthetic series as it efficiently blocked SOCE at a low concentration (10 µM) but did not completely deplete internal stores at higher concentrations (50 µM). While structurally very close isomers like ***m*-Br-2APB** and ***p*-Br-2APB** did not show different behaviour in blocking *I*_CRAC_, they had a strikingly different effect on MDA-MB-231 cells viability.

## Figures and Tables

**Scheme A1:**
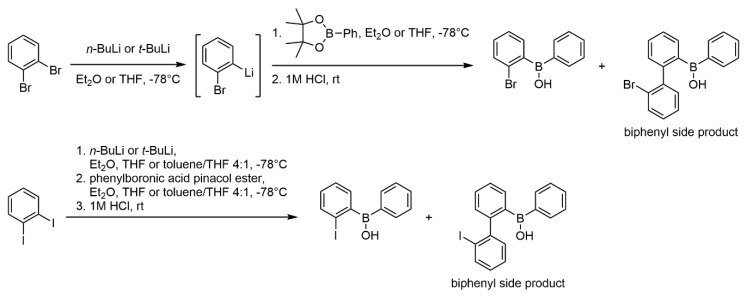
Lithiation of 1,2-dibromo- or 1,2-diiodobenzene, and subsequent borination of the organolithium species led to significant formation of biphenyl side products that were inseparable from the desired (aryl)(phenyl)borinic acids.

## Supplementary Materials

The following are available online at https://www.mdpi.com/1422-0067/21/16/5604/s1. Supplementary data (adapted synthesis of **DPB162-AE** (Scheme S1), **GSK-7975A** (Scheme S2), and **Synta66** (Scheme S3), crystal structures of **GSK-7975A** (Figure S1), **cBu-2APB** (Figure S2), ***p*-SO_2_NMe_2_-2APB** (Figure S3), ***o*-F-2APB** (Figure S4), and ***o*-Cl-2APB** (Figure S5), SOCE blockade by **GSK-7975A** and **Synta66** with pre-incubation (Figure S6), SOCE in STIM1 KO and Orai1 KO cells (Figure S7) and copies of ^1^H, ^11^B, ^13^C, and ^19^F NMR spectra of all final compounds).

## Abbreviations

2-APB	2-Aminoethyl diphenylborinate
AUC	Area under the curve
CD	Current density
CRAC	Ca^2+^ release-activated Ca^2+^ current
ER	Endoplasmic reticulum
FLIPR	Fluorometric imaging plate reader
IP_3_	Inositol 1,4,5-triphosphate
NCF	Nominal calcium-free (buffer)
PI(4,5)P_2_	Phosphatidylinositol 4,5-bisphosphate
PM	Plasma membrane
SERCA	Sarco/endoplasmic reticulum Ca^2+^-ATPase
SOCE	Store-operated calcium entry
STIM	Stromal interaction molecule
Tg	Thapsigargin

## Appendix A

When we used the general lithiation/borination protocol with 1,2-dibromobenzene (Scheme A1) we observed significant attack of the aryl lithium species at the halogenated carbon of the borinic intermediate. This led to the shown biphenyl side product which we were unable to separate from the desired borinic acid by chromatography or crystallization. Changing the solvent to toluene/THF 4:1 to carry out the halogen lithium exchange and subsequent reaction with phenylboronic acid pinacol ester suppressed the formation of the biphenyl side product and enabled us to isolate pure (2-bromophenyl)(phenyl) borinic acid. The solvent change strategy did not work with 1,2-diiodobenzene, however, and gave inseparable mixtures of desired (2-iodophenyl)(phenyl)borinic acid and biphenyl side product.
