# Supplementary material for: Synthesis and Pharmacological Characterization of 2-Aminoethyl Diphenylborinate (2-APB) Derivatives for Inhibition of Store-Operated Calcium Entry (SOCE) in MDA-MB-231 Breast Cancer Cells
Source: Int J Mol Sci. 2020 Aug 5;21(16):5604. doi: 10.3390/ijms21165604 (PMC7460636; doi:10.3390/ijms21165604)
Supplement: Supplementary file 1 [file ijms-21-05604-s001.pdf]

SUPPLEMENTARY MATERIALS

for

Synthesis and Pharmacological Characterization of 2-Aminoethyl Diphenylborinate (2-APB) Derivatives for Inhibition of Store-operated Calcium Entry (SOCE) in MDA-MB-231 Breast Cancer Cells

Achille Schild[1,#], Rajesh Bhardwaj[2,#], Nicolas Wenger[2], Dominic Tscherrig[1], Palanivel Kandasamy[2], Jan Dernič[2], Roland Baur[1], Christine Peinelt[1], Matthias A. Hediger[2,*] and Martin Lochner[1,*]

[1]*Institute of Biochemistry and Molecular Medicine, University of Bern, Bühlstrasse 28, 3012 Bern, Switzerland*

[2]*Department of BioMedical Research, University of Bern, Murtenstrasse 35, 3008 Bern, Switzerland*

Table of Contents

1. Adapted Synthesis of Dimeric 2-APB Analogue **DPB162-AE** (*Scheme S1*) S2
2. Synthesis of **GSK-7975A** and **Synta66** (*Schemes S2* and *S3*)..S3
3. Crystal Structures of Compounds (*Figures S1-S5*) S7
4. SOCE Blockade by **GSK-7975A** and **Synta66** with Pre-incubation (*Figure S6*).......................S10
5. SOCE in STIM1 KO and Orai1 KO MDA-MB-231 Cells (*Figure S7*) S11
6. Copies of ^{1}H, ^{11}B, ^{13}C and ^{19}F NMR Spectra of 2-APB Analogues S12-S64

1. Adapted Synthesis of Dimeric 2-APB Analogue DPB162-AE

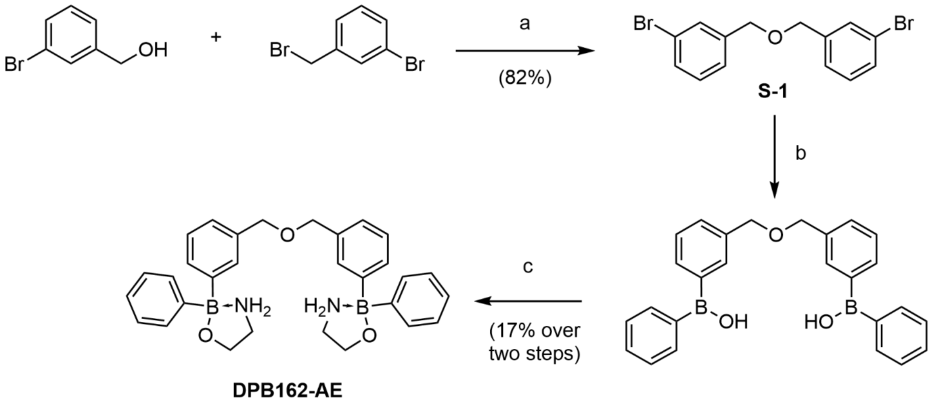

Scheme S1. Synthesis of **DPB162-AE**. Reagents and conditions: (a) NaH, THF, -15°C to rt; then reflux. (b) *t*-BuLi, THF, -78°C; (*i*-PrO)$_3$B, THF, -78°C; PhLi, -78°C to rt; then 1M HCl, rt. (c) 2-aminoethanol, EtOH, rt.

3,3'-(Oxybis(methylene))bis(bromobenzene) (S-1): A solution of 3-bromobenzyl alcohol (2.38 mL, 20 mmol) and 3-bromobenzyl bromide (5 g, 20 mmol) in dry THF (40 mL) was cooled to -15°C. NaH (960 mg, 60 % dispersion in mineral oil, 24 mmol) was added in small portions over a period of 5 min. The mixture was allowed to warm up to rt and was heated at reflux overnight. The mixture was quenched by the addition of water (40 mL) and washed with Et_2O (3x50 mL). All organic phases were combined and dried over Na_2SO_4. The solvent was removed under reduced pressure. The residue was purified by flash chromatography (CombiFlash, silica gel, gradient with cyclohexane and ethyl acetate) affording **S-1** as a colourless oil (5.85 g, 82%). ^{1}H NMR (300 MHz, DMSO-d_6) δ 7.57 – 7.53 (m, 2H), 7.50 (dt, *J* = 7.2, 1.9, 2H), 7.40 – 7.29 (m, 4H), 4.55 (s, 4H). ^{13}C NMR (75 MHz, DMSO-d_6) δ 141.09, 130.54, 130.32, 130.04, 126.40, 121.63, 70.66.

2,2'-((Oxybis(methylene))bis(3,1-phenylene))bis(2-phenyl-1,3,2λ^4-oxazaborolidine) (DPB162-AE): A solution of **S-1** (800 mg, 2.25 mmol) in dry THF (20 mL) was cooled to -78°C. *t*-BuLi solution (6.08 mL, 1.7M in pentane, 10.34 mmol) was added dropwise. The resulting mixture was stirred at -78°C for 30 min. A solution of (*i*-PrO)$_3$B (1.04 mL, 4.5 mmol) in THF (5 mL) was added dropwise to the mixture. The mixture was stirred at -78°C for 3 h. Phenyllithium solution (2.5 mL, 1.8M in Bu_2O, 4.5 mmol) was added dropwise to the mixture. The dark brown mixture was allowed to warm up to rt overnight. The mixture was quenched by the addition of 1M aqueous HCl (25 mL). The resulting two phases were separated. The aqueous phase was washed with Et_2O (2 x 20 mL). All organic phases were combined and washed with 1M aq. HCl (20 mL) and dried over Na_2SO_4. The solvent was removed under reduced pressure. The residue was was purified by flash chromatography (CombiFlash, silica gel, gradient with cyclohexane and ethyl acetate) affording a viscous oil (485 mg). The oil was dissolved in a mixture of absolute EtOH (4 mL) and Et_2O (4 mL). 2-Aminoethanol (140 µL, 2.32 mmol) was added and the mixture was stirred at rt for 1 h. The solvent was removed under reduced pressure and the residue was further dried in vacuo. The residual oil was mixed with Et_2O.

After the undissolved parts of the mixture settled, the liquid was removed. The residue was suspended in *i*-Pr_2O using an ultrasonic bath. After the undissolved parts settled, the liquid was removed, and the residue was dried in vacuo. The residue was mixed with *i*-PrOH (4 mL) and *i*-Pr_2O (2 mL) at reflux temperature. The resulting mixture was stirred under reflux for 30 min. During reflux of the mixture a white solid started to precipitate. To further increase the amount of precipitate the mixture was cooled down to -20°C. After no more precipitate formed, the liquid was removed and the residue was dried in vacuo affording **DPB162-AE** as a white powder (190 mg, 17%). ^{1}H NMR (300 MHz, DMSO-d_6) δ 7.43 – 7.34 (m, 6H), 7.36 – 7.27 (m, 2H), 7.18 – 7.06 (m, 6H), 7.08 – 6.96 (m, 4H), 6.06 (s, 4H), 4.40 (s, 4H), 3.75 (t, *J* = 6.5, 4H), 2.81 (p, *J* = 6.2, 4H). ^{11}B NMR (96 MHz, DMSO) δ 4.28. ^{13}C NMR (75 MHz, DMSO) δ 136.20, 131.49, 131.05, 130.74, 126.60, 126.49, 124.88, 124.56, 117.00, 72.25, 62.40, 41.35. MS (ESI+) *m/z* 493.28 $[M+H]^+$. HRMS (ESI+) *m/z* calc. for $C_{30}H_{35}O_3N_2B_2$ $[M+H]^+$: 493.2835; found 493.2828.

2. Synthesis of GSK-7975A and Synta66

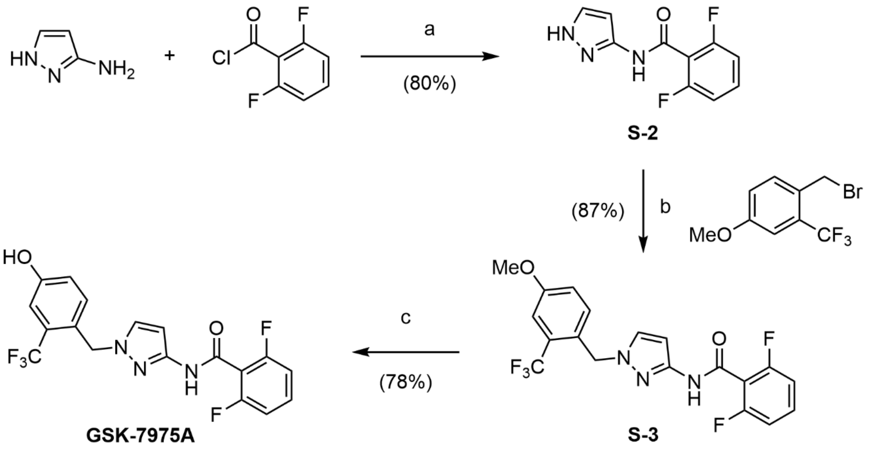

Scheme S2. Synthesis of **GSK-7975A**. Reagents and conditions: (a) Et_3N, MeCN, 0°C to rt. (b) 1-(bromomethyl)-4-methoxy-2-(trifluoromethyl)benzene, $LiN(SiMe_3)_2$, THF, rt. (c) BBr_3, CH_2Cl_2, rt.

2,6-Difluoro-*N*-(1*H*-pyrazol-3-yl)benzamide (S-2): To a stirred solution of 3-aminopyrazole (831 mg, 10 mmol) in MeCN (25 mL) at 0°C Et_3N (2.77 mL, 20 mmol) was added dropwise. The reaction mixture was stirred at 0°C for 30 min, then a solution of 2,6-difluorobenzoyl chloride (1.77 g, 10 mmol) in MeCN (25 mL) was slowly added keeping the temperature between 0-7°C for one hour. The reaction was then allowed to warm up to rt and stirred overnight. The mixture was concentrated under reduced pressure and the residue partitioned between water and EtOAc. The aqueous layer was separated and extracted with EtOAc (2x). The combined organics were washed with brine, dried over Na_2SO_4, filtered and concentrated under reduced pressure to give a yellow solid. Purification of the crude product by flash column chromatography on silica (gradient: 100% cyclohexane to cyclohexane/EtOAc 70:30) provided the product as a white solid (1.79 g, 80%). ^{1}H NMR (400 MHz, DMSO-d_6) δ 12.47 (s, 1H), 11.20 (s, 1H), 7.71-7.66 (m, 1H), 7.61-7.47 (m, 1H), 7.19 (t, *J* = 8.0, 2H), 6.67-6.54 (m, 1H). ^{13}C NMR (101 MHz,

DMSO-d_6) δ 158.9 (dd, *J* = 248.2, 7.8), 157.4, 146.5, 131.8 (t, *J* = 10.0), 128.9, 115.3 (t, *J* = 22.8), 112.2-111.4 (m), 96.4. ^{19}F NMR (282 MHz, DMSO-d_6) δ -113.28 to -113.42 (m), -113.93 to -114.21 (m). HRMS (ESI+) *m/z* calc. for $C_{10}H_8ON_3F_2$ [M+H]$^+$: 224.0630; found: 224.0628.

2,6-Difluoro-*N*-(1-(4-methoxy-2-(trifluoromethyl)benzyl)-1*H*-pyrazol-3-yl)benzamide (S-3): A solution of **S-2** (1.30 g, 5.8 mmol) in THF (50 mL) was treated with 1M lithium bis(trimethylsilyl)amide in THF (5.8 mL, 5.8 mmol) and stirred at ambient temperature for 15 min. 1-(bromomethyl)-4-methoxy-2-(trifluoromethyl)benzene (1.42 g, 5.3 mmol) in THF (2 mL) was added and the mixture stirred for 24 h. The solution was concentrated in vacuo and the residue partitioned between $CHCl_3$ and sat. aq. $NaHCO_3$. The organic layer was separated and the aqueous extracted with $CHCl_3$ (2x). The combined extracts were washed with brine, dried over $MgSO_4$ and concentrated. Flash column chromatography on silica (gradient: 100% cyclohexane to cyclohexane/EtOAc 70:30) gave a solid, white product (1.9 g, 87%). ^{1}H NMR (300 MHz, DMSO-d_6) δ 11.29 (s, 1H), 7.78 (d, *J* = 2.3, 1H), 7.58-7.47 (m, 1H), 7.27-7.21 (m, 2H), 7.17 (t, *J* = 8.1, 2H), 7.09-7.01 (m, 1H), 6.65 (d, *J* = 2.3, 1H), 5.37 (s, 2H), 3.82 (s, 3H). ^{13}C NMR (101 MHz, DMSO-d_6) δ 158.9 (dd, *J* = 248.4, 7.7), 158.7, 157.45, 146.9, 131.9 (t, *J* = 10.0), 131.8, 131.6, 127.5 (q, *J* = 30.4), 126.8 (q, *J* = 1.6), 123.9 (q, *J* = 274.2), 117.9, 115.0 (t, *J* = 22.7), 112.0-111.7 (m), 111.6 (q, *J* = 5.8), 97.4, 55.6, 50.8 (q, *J* = 2.7). ^{19}F NMR (282 MHz, DMSO-d_6) δ -114.1. HRMS (ESI+) *m/z* calc. for $C_{19}H_{15}O_2N_3F_5$ [M+H]$^+$: 412.1079; found: 412.1081.

2,6-Difluoro-*N*-(1-(4-hydroxy-2-(trifluoromethyl)benzyl)-1*H*-pyrazol-3-yl)benzamide (GSK-7975A): S-3 (2.04 g, 4.95 mmol) was dissolved in CH_2Cl_2 (35 mL), then treated with boron tribromide (1M in CH_2Cl_2, 9.9 mL, 9.9 mmol) at ambient temperature and stirred under argon for 18 h. The mixture was treated with water and partitioned with $CHCl_3$. The layers were separated, and the aqueous phase extracted once more with $CHCl_3$. The combined extracts were dried over Na_2SO_4, filtered and concentrated. The residue was purified by silica column chromatography (gradient: 100% cyclohexane to cyclohexane/EtOAc 55:45). This gave the final compound **GSK-7975A** as a white solid (1.54 g, 78%). Single crystals for X-ray measurements were obtained by recrystallization from CH_2Cl_2/*n*-hexane. ^{1}H NMR (300 MHz, DMSO-d_6) δ 11.29 (s, 1H), 7.78 (d, *J* = 2.3, 1H), 7.58-7.47 (m, 1H), 7.27-7.21 (m, 2H), 7.17 (t, *J* = 8.1, 2H), 7.09-7.01 (m, 1H), 6.65 (d, *J* = 2.3, 1H), 5.37 (s, 2H), 3.82 (s, 3H). ^{13}C NMR (101 MHz, DMSO-d_6) δ 161.0 (dd, *J* = 250.4, 7.2), 160.5 (s), 158.7 (s), 148.1 (s), 133.3 (t, *J* = 10.1), 132.7 (s), 132.4 (s), 129.8 (q, *J* = 30.6), 126.4 (d, *J* = 1.5), 125.6 (dd, *J* = 546.4, 273.2), 120.1 (s), 115.9 (t, *J* = 21.8), 113.9 (q, *J* = 5.8), 113.2 – 112.4 (m), 99.3 (s), 52.6 (dd, *J* = 5.6, 2.7). ^{19}F NMR (282 MHz, DMSO-d_6) δ -58.6 (s), -114.0 (t, *J* = 6.9). HRMS (ESI+) *m/z* calc. for $C_{18}H_{13}O_2N_3F_5$ [M+H]$^+$: 398.0922; found: 398.0912. UPLC (254 nm) t_R = 3.30 min, 99.7% purity.

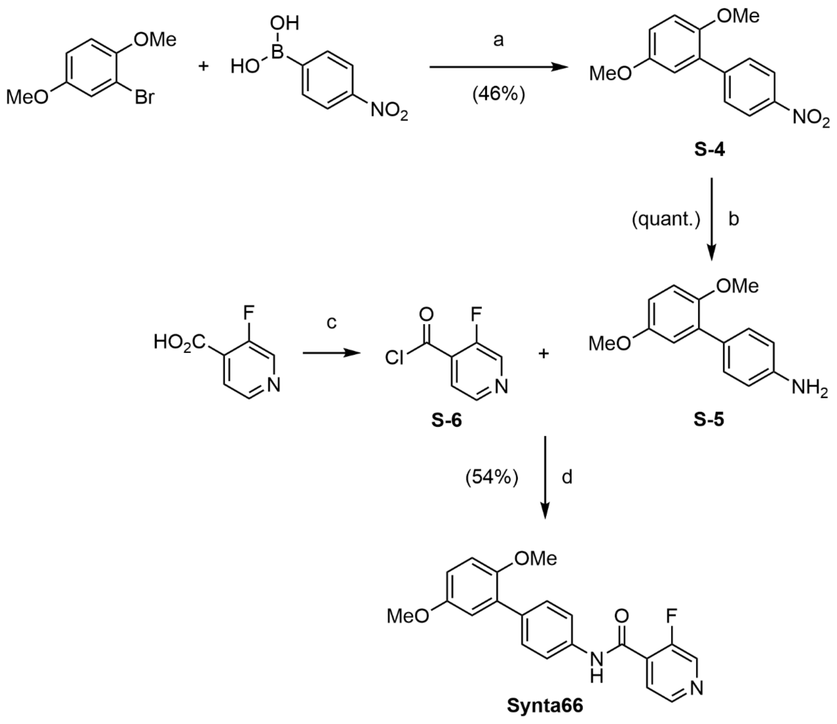

Scheme S3. Synthesis of **Synta66**. Reagents and conditions: (a) $PdBzCl(PPh_3)_2$ (*cat.*), K_2CO_3, NMP, reflux. (b) $SnCl_2$, $CH_2Cl_2/EtOH/H_2O$ (1:1:0.03), rt, quant. (c) $SOCl_2$, DMF (*cat.*), reflux. (d) Et_3N, CH_2Cl_2, rt.

2,5-Dimethoxy-4'-nitro-1,1'-biphenyl (S-4): 2-bromo-1,4-dimethoxybenzene (434 mg, 2 mmol) and (4-nitrophenyl)boronic acid (334 mg, 2 mmol), catalyst $PdBzCl(PPh_3)_2$ (76 mg, 0.1 mmol) and K_2CO_3 (1.38 g, 10 mmol) were transferred to a 25 mL round-bottom flask and put under argon atmosphere. Then dry NMP (10 mL) was added and the mixture heated under reflux for two days. After TLC indicated the completion of the Suzuki coupling, the solvent was evaporated, and the residue taken up in H_2O and EtOAc. The layers were separated, and the aqueous layer extracted with EtOAc (2x). The combined organics were dried (Na_2SO_4) filtered and the solvent was evaporated. The crude product was further purified by flash column chromatography on silica gel (gradient: 100% cyclohexane to cyclohexane/EtOAc 90:10) to yield the product as a yellow solid (238 mg, 46%). ^{1}H NMR (300 MHz, DMSO-d_6) δ 8.26 (d, J = 8.9, 2H), 7.79 (d, J = 8.9, 2H), 7.11 (d, J = 8.9, 1H), 7.01 (dd, J = 8.9, 3.1, 1H), 6.96 (d, J = 3.1, 1H), 3.76 (s, 3H), 3.74 (s, 3H). ^{13}C NMR (75 MHz, Methanol-d_4) δ 153.4, 150.2, 146.2, 144.9, 130.5, 128.2, 123.1, 115.9, 115.0, 113.3, 56.2, 55.5. HRMS (ESI+) *m/z* calc. for $C_{14}H_{14}O_4N$ $[M+H]^+$: 260.0928; found: 260.0926.

2',5'-Dimethoxy-[1,1'-biphenyl]-4-amine (S-5): Biphenyl **S-4** was dissolved in a mixture of CH_2Cl_2 and EtOH (1:1, 10 mL). $SnCl_2$ was added followed by the addition of water (300 µL). After stirring at rt for two days, the mixture was neutralized with 2M aq. NaOH solution, filtered over celite and the residue was washed with EtOAc. The layers were separated, and the aqueous layer extracted with EtOAc (2x). The combined organics were washed with brine, dried over Na_2SO_4, filtered and reduced in vacuo to give the product in quantitative yield as a white solid. ^{1}H NMR (300 MHz, DMSO-d_6) δ 7.21 (d, J = 8.4, 2H), 6.96 (d, J = 8.8, 1H), 6.85-6.74 (m, 2H), 6.65 (d, J = 8.4, 2H), 5.75 (s, 1H), 3.72 (s, 3H),

3.67 (s, 3H). ^{13}C NMR (101 MHz, DMSO-d_6) δ 155.4, 152.2, 147.7, 133.5, 131.1, 129.6, 117.3, 116.1, 114.4, 113.0, 56.9, 56.1. HRMS (ESI+) *m/z* calc. for $C_{14}H_{16}O_2N$ [M + H]$^+$: 230.1176; found: 230.1173.

3-Fluoroisonicotinoyl chloride (S-6): A mixture of 3-fluoroisonicotinic acid (1.41 g, 10 mmol), thionyl chloride (5 mL, 69 mmol) and a few drops of DMF were heated to reflux overnight. Reaction control was done by TLC, treating of the probe with methanol before spotting to create the methylester. After TLC control showed completion of the reaction, the mixture was allowed to cool to rt and was concentrated under reduced pressure to obtain **S-6** as a yellowish oil (1.17 g). The crude product was used in the next step without further purification. ^{1}H NMR (300 MHz, DMSO-d_6) δ 8.73 (d, *J* = 2.4, 1H), 8.57 (d, *J* = 4.9, 1H), 7.82-7.70 (m, 1H).

***N*-(2',5'-Dimethoxy-[1,1'-biphenyl]-4-yl)-3-fluoroisonicotinamide (Synta66):** To a solution of amine **S-5** (100 mg, 0.44 mmol) in CH_2Cl_2 (5 mL), acid chloride **S-6** (70 mg, 0.44 mmol) dissolved in CH_2Cl_2 (1 mL) was added, followed by the dropwise addition of Et_3N (53 mg, 0.52 mmol, 73 µL). TLC control after stirring one hour at rt indicated completion of the reaction. The mixture was taken up in H_2O and CH_2Cl_2 and the layers were separated. The aqueous layer was extracted with CH_2Cl_2 (2x) and the combined organics washed with brine and dried over Na_2SO_4. Filtration and evaporation of the solvent followed by flash column chromatography (gradient: 100% cyclohexane to cyclohexane/EtOAc 50:50) resulted in the desired product as a white solid (83 mg, 54%). ^{1}H NMR (300 MHz, DMSO-d_6) δ 10.74 (s, 1H), 8.78 (d, *J* = 1.2, 2H), 8.60 (dd, *J* = 4.8, 1.2, 1H), 7.76-7.68 (m, 3H), 7.51 (d, *J* = 8.7, 2H), 7.04 (d, *J* = 8.7, 1H), 6.94-6.84 (m, 2H), 3.75 (s, 3H), 3.71 (s, 3H). ^{13}C NMR (101 MHz, $CDCl_3$) δ 159.3, 156.2 (d, *J* = 256.1), 154.2, 151.1, 147.2 (d, *J* = 5.1), 139.5 (d, *J* = 27.6), 136.0 (d, *J* = 22.6), 131.1, 130.4, 128.4 (d, *J* = 9.6), 124.8, 120.4, 116.9, 113.5, 113.3, 56.6, 56.0. ^{19}F NMR (282 MHz, DMSO-d_6) δ -129.88 (s). HRMS (ESI+) *m/z* calc. for $C_{20}H_{18}O_3N_2F$ [M+H]$^+$: 353.1296; found: 353.1300. UPLC (254 nm) t_R = 3.52 min, 99.9% purity.

3. Crystal Structures of Compounds

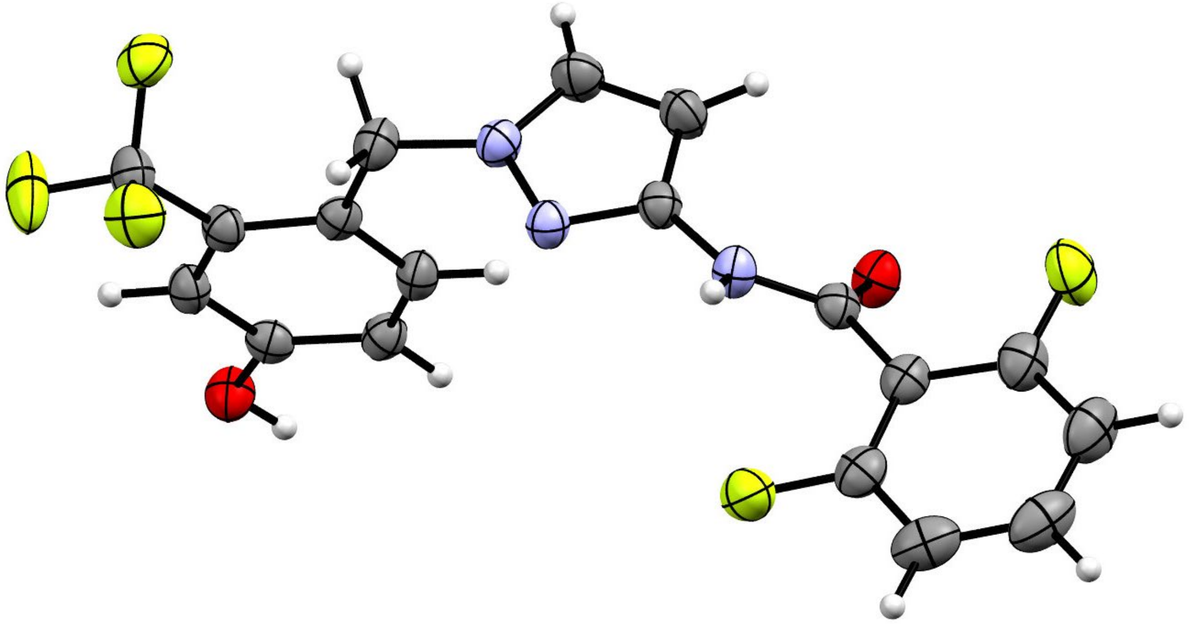

Figure S1. Crystal structure of **GSK-7975A**. Thermal ellipsoids are shown at 50% probability level. Carbons grey, hydrogens white, oxygens red, nitrogens blue, fluorines green-yellow.

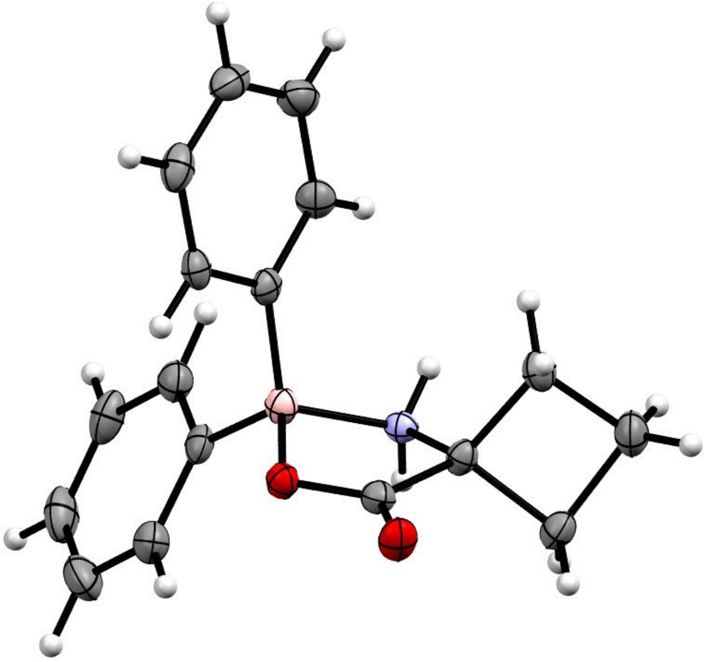

Figure S2. Crystal structure of **cBu-2APB**. Thermal ellipsoids are shown at 50% probability level. Carbons grey, hydrogens white, oxygens red, nitrogens blue, boron pink. Selected bond lengths [Å] and bond angles [°]: B-N 1.63, B-O 1.53, B-C 1.61; N-B-O 97.8, C-B-C 115.6.

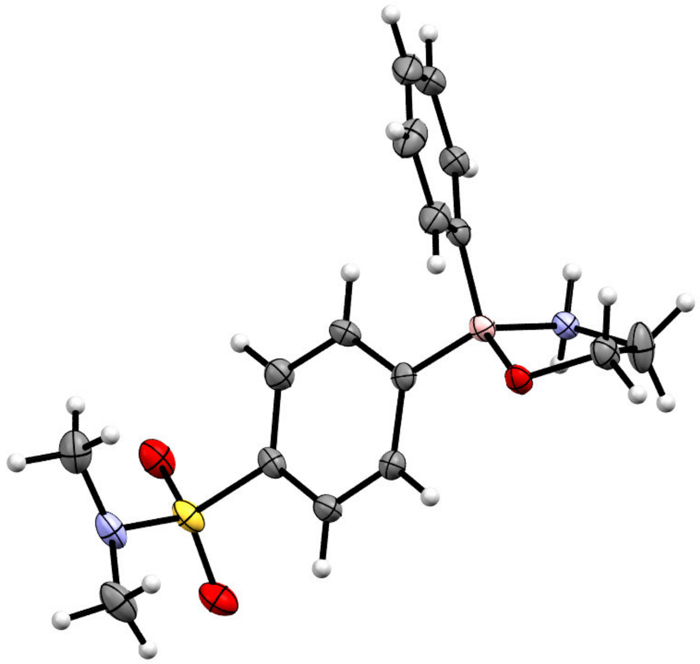

Figure S3. Crystal structure of ***p*-SO_2NMe_2-2APB**. Thermal ellipsoids are shown at 50% probability level. Carbons grey, hydrogens white, oxygens red, nitrogens blue, sulfur yellow, boron pink. Selected bond lengths [Å] and bond angles [°]: B-N 1.65, B-O 1.47, B-C 1.62, N-B-O 99.2, C-B-C 113.1.

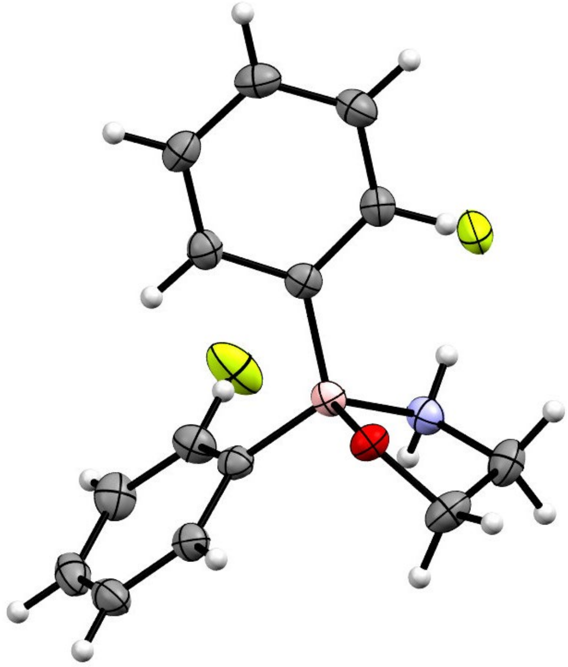

Figure S4. Crystal structure of ***o*-F-2APB**. Thermal ellipsoids are shown at 50% probability level. Carbons grey, hydrogens white, oxygens red, nitrogens blue, fluorines green-yellow, boron pink. The phenyl and fluorophenyl groups are mutually disordered in the structure (75%:25% in the crystal

examined). Selected bond lengths [Å] and bond angles [°]: B-N 1.65, B-O 1.49, B-C 1.62, N-B-O 100.7, C-B-C 114.2.

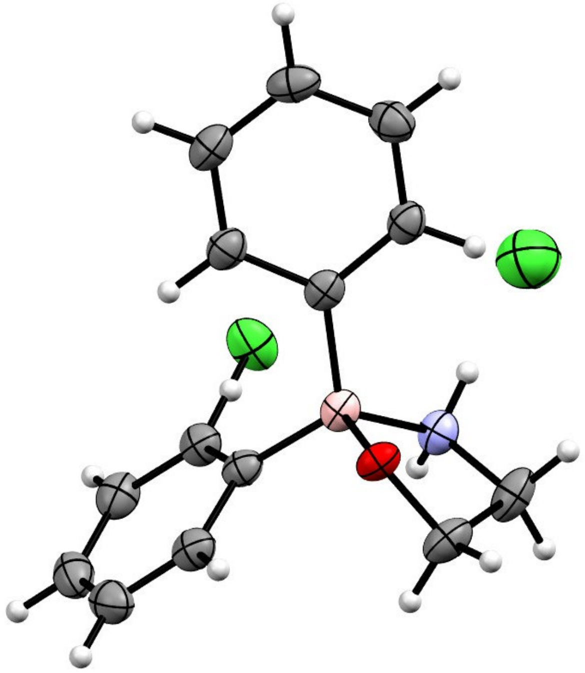

Figure S5. Crystal structure of ***o*-Cl-2APB**. Thermal ellipsoids are shown at 50% probability level. Carbons grey, hydrogens white, oxygens red, nitrogens blue, chlorines green, boron pink. The phenyl and chlorophenyl groups are mutually disordered in the structure (90%:10% in the crystal examined). Selected bond lengths [Å] and bond angles [°]: B-N 1.66, B-O 1.48, B-C 1.62, N-B-O 100.2, C-B-C 115.3.

4. SOCE Blockade by GSK-7975A and Synta66 with Pre-incubation

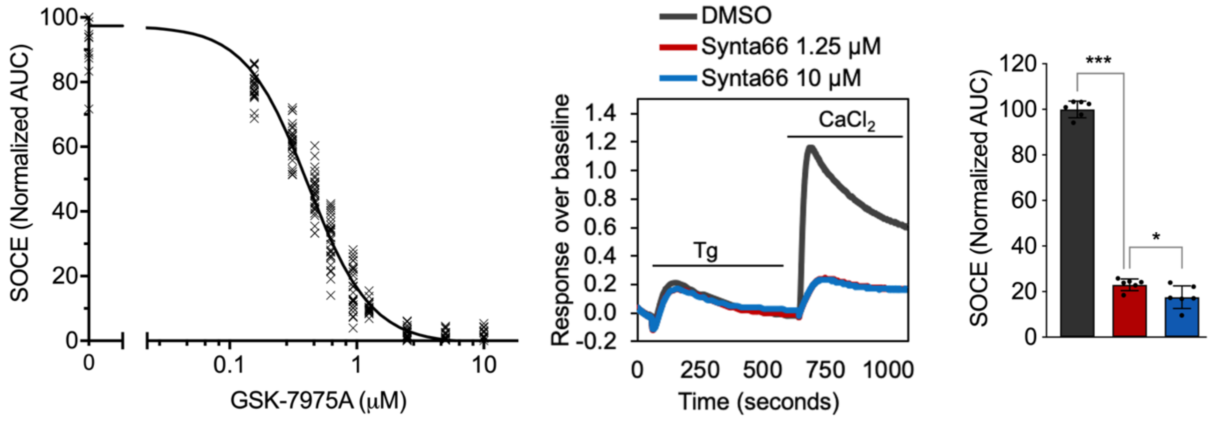

(a) (b) (c)

Figure S6. SOCE blockade by GSK-7975A and Synta66 with a total incubation time of 30 min. (a) Dose response curve of GSK-7975A on SOCE in MDA-MB-231 cells incubated for 30 min with the compound (IC_{50} = 0.3971 to 0.4336 μM). Data shown are mean ± SD of the normalized AUC of the SOCE traces (n ≥ 20/concentration). (b) SOCE traces of MDA-MB-231 cells pre-treated for 20 min with DMSO or indicated doses of Synta66 before the addition of thapsigargin (30 min total incubation time before the addition of calcium chloride). Thapsigargin (1 μM Tg) and calcium chloride (2 mM $CaCl_2$) administrations are indicated on the traces. (c) Mean ± SD of the normalized AUC of the SOCE traces shown in (b) (n = 6/concentration). Statistical significance is indicated as "***" for $p \leq 0.001$ and "*" for $0.01 < p \leq 0.05$.

5. SOCE in STIM1 KO and Orai1 KO MDA-MB-231 Cells

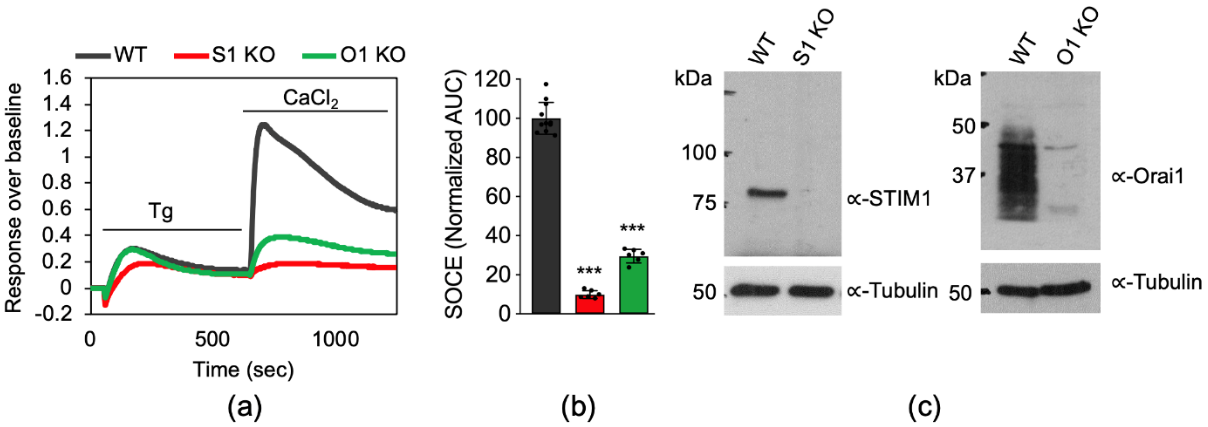

Figure S7. Knock-out of STIM1 or Orai1 in MDA-MB-231 cells reduces SOCE. (**a**) FLIPR recorded intracellular calcium signal in WT (black trace), STIM1 KO (red trace) or Orai1 KO (green trace) MDA-MB-231 cells. Black bars above traces indicate duration of application of thapsigargin (Tg, to deplete ER calcium stores) and $CaCl_2$ (2 mM final concentration). (**b**) Quantification of calcium influx after $CaCl_2$ application (AUC, mean ± SD), normalized to influx in WT cells ($n \geq 6$ for all data points). Statistical significance is indicated as "***" for $p \leq 0.001$. (**c**) Western blots of STIM1 KO and Orai1 KO MDA-MB-231 cells.

6. Copies of ^{1}H, ^{11}B, ^{13}C and ^{19}F NMR Spectra of 2-APB Analogues

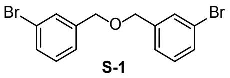

S-1

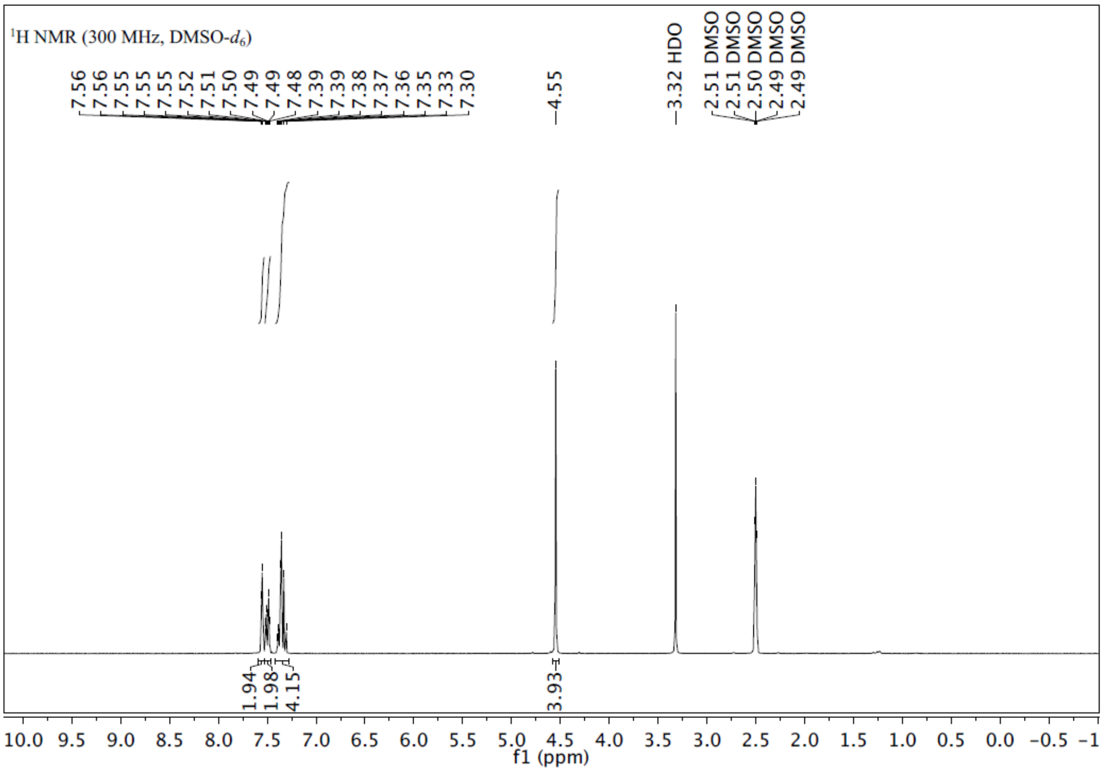

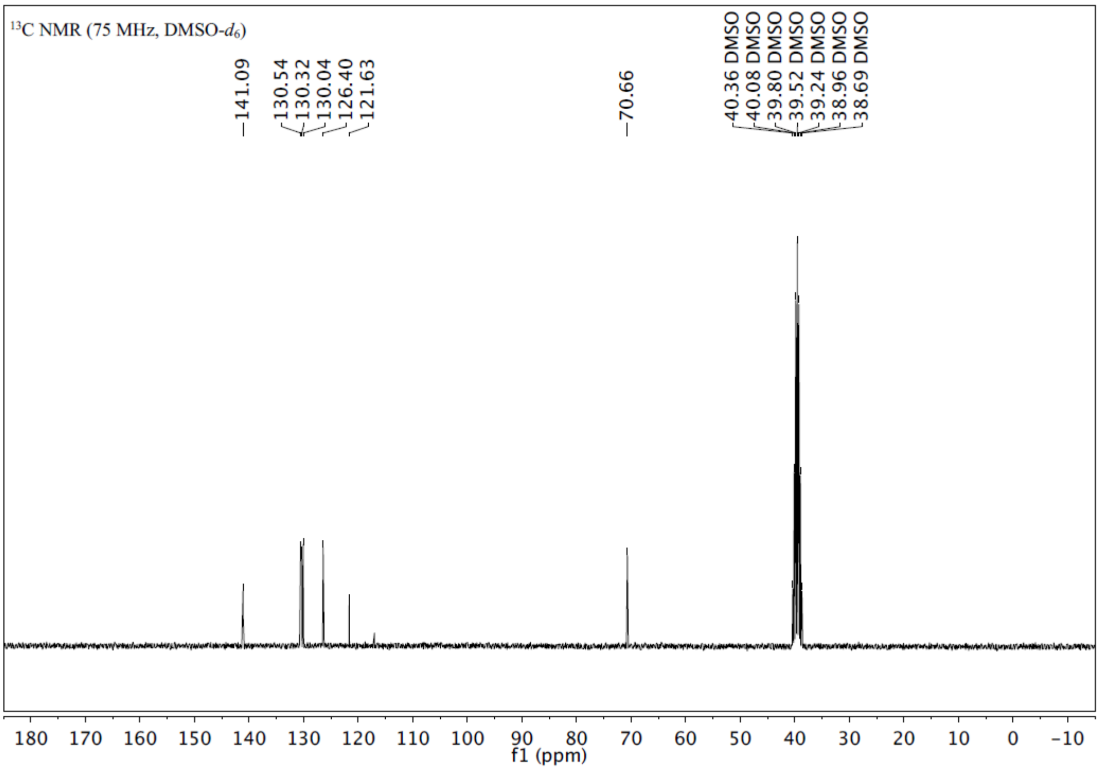

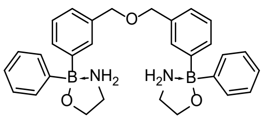
B
NH2
O
H2N
B
O

DPB162-AE

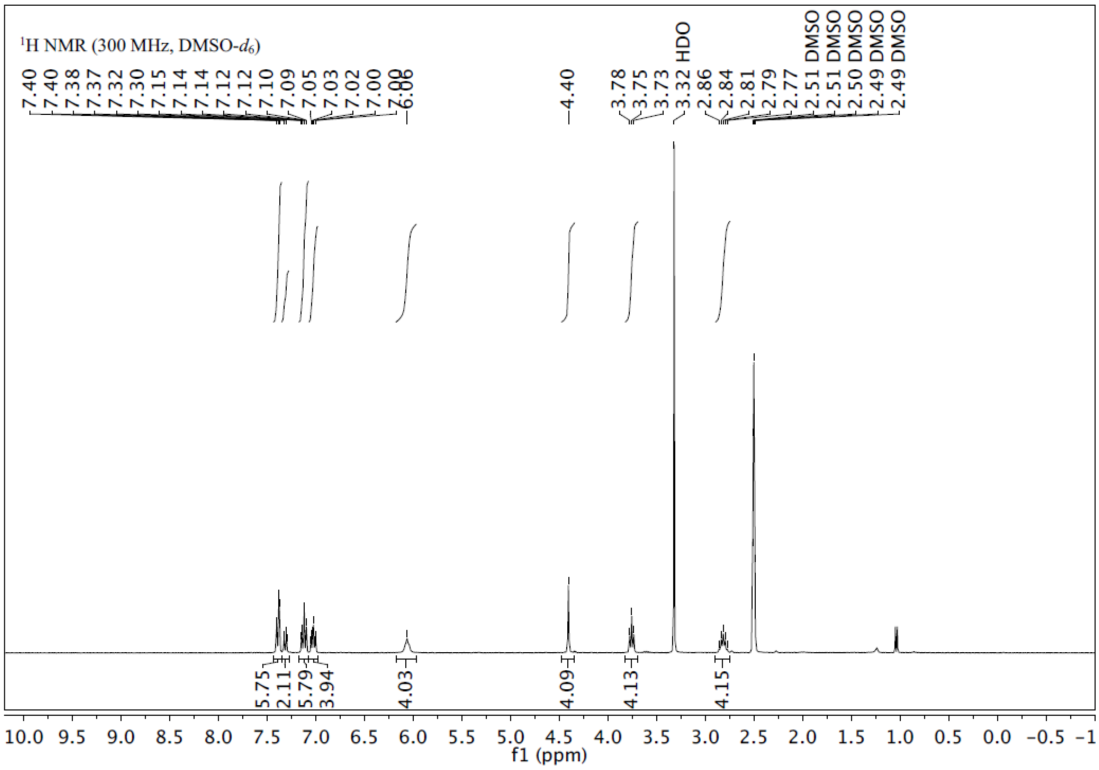
1H NMR (300 MHz, DMSO-d6)
7.40 7.40 7.38 7.37 7.32 7.30 7.15 7.14 7.14 7.12 7.12 7.10 7.09 7.05 7.03 7.02 7.00
4.40
3.78 3.75 3.73
3.32 HDO
2.86 2.84 2.81 2.79 2.77
2.51 DMSO 2.51 DMSO 2.50 DMSO 2.49 DMSO 2.49 DMSO
5.75 2.11 5.79 3.94 4.03 4.09 4.13 4.15
10.0 9.5 9.0 8.5 8.0 7.5 7.0 6.5 6.0 5.5 5.0 4.5 4.0 3.5 3.0 2.5 2.0 1.5 1.0 0.5 0.0 −0.5 −1
f1 (ppm)

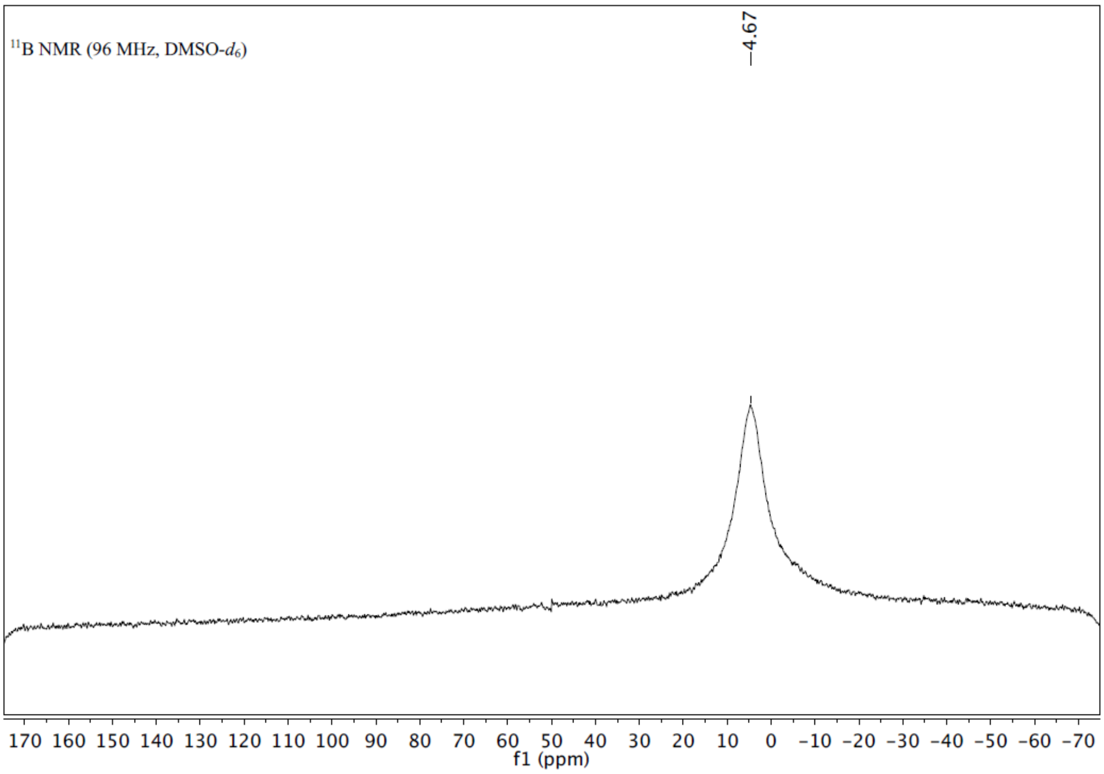
11B NMR (96 MHz, DMSO-d6)
4.67
170 160 150 140 130 120 110 100 90 80 70 60 50 40 30 20 10 0 −10 −20 −30 −40 −50 −60 −70
f1 (ppm)

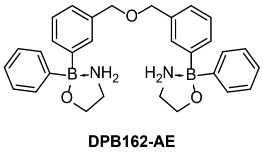

DPB162-AE

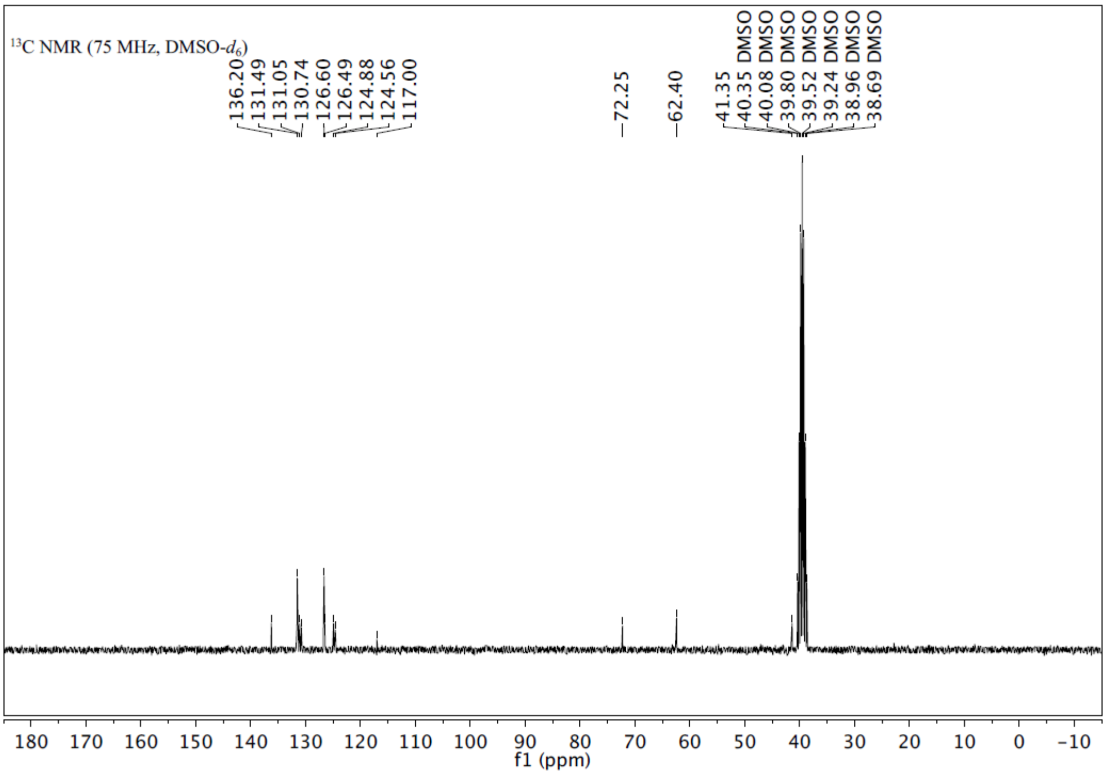

^{13}C NMR (75 MHz, DMSO-d_6)
136.20
131.49
131.05
130.74
126.60
126.49
124.88
124.56
117.00
72.25
62.40
41.35
40.35 DMSO
40.08 DMSO
39.80 DMSO
39.52 DMSO
39.24 DMSO
38.96 DMSO
38.69 DMSO
180 170 160 150 140 130 120 110 100 90 80 70 60 50 40 30 20 10 0 −10
f1 (ppm)

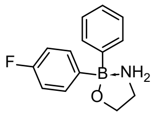
F
B
NH2
O

***p*-F-2APB**

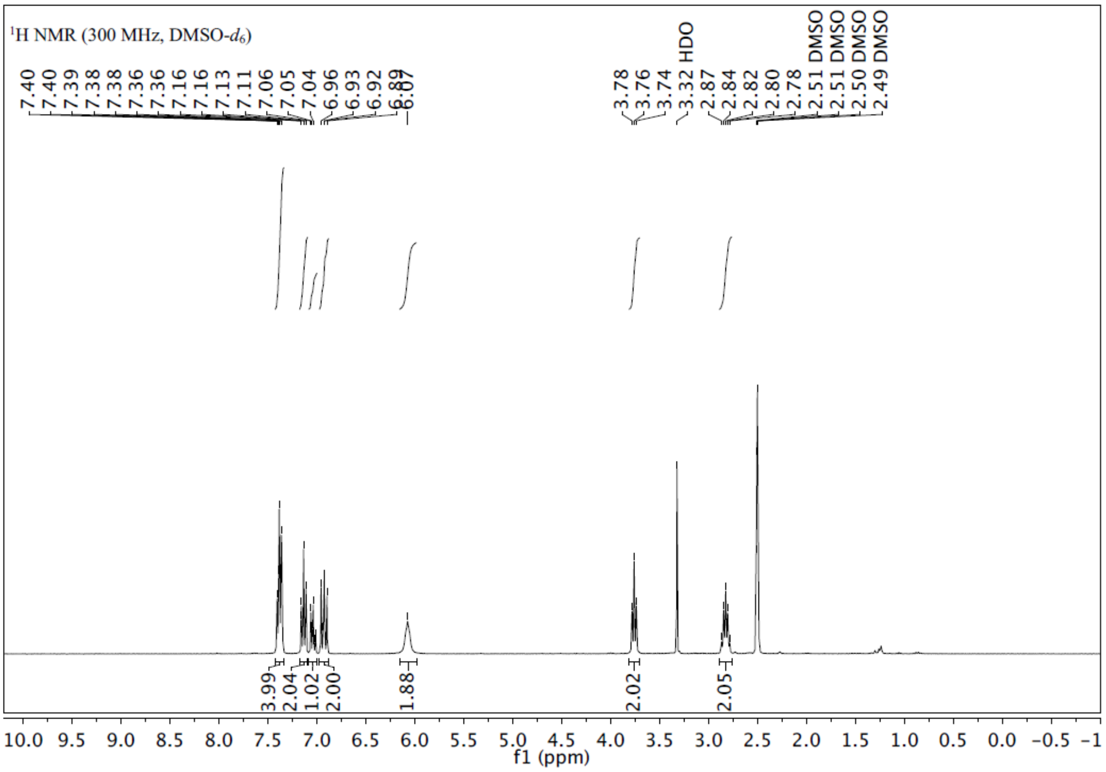
1H NMR (300 MHz, DMSO-d6)
7.40 7.40 7.39 7.38 7.38 7.36 7.36 7.16 7.16 7.13 7.11 7.06 7.05 7.04 6.96 6.93 6.92 6.89 6.07
3.78 3.76 3.74 3.32 HDO 2.87 2.84 2.82 2.80 2.78 2.51 DMSO 2.51 DMSO 2.50 DMSO 2.49 DMSO
3.99 2.04 1.02 2.00 1.88 2.02 2.05
10.0 9.5 9.0 8.5 8.0 7.5 7.0 6.5 6.0 5.5 5.0 4.5 4.0 3.5 3.0 2.5 2.0 1.5 1.0 0.5 0.0 −0.5 −1
f1 (ppm)

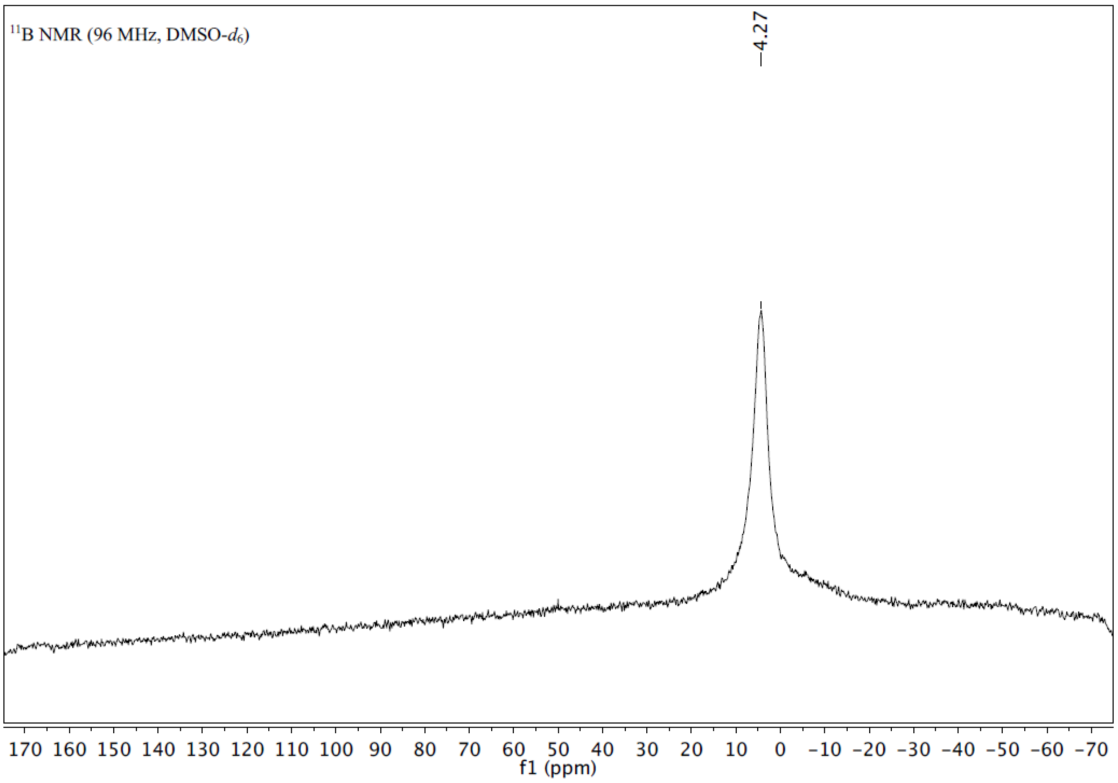
11B NMR (96 MHz, DMSO-d6)
4.27
170 160 150 140 130 120 110 100 90 80 70 60 50 40 30 20 10 0 −10 −20 −30 −40 −50 −60 −70
f1 (ppm)

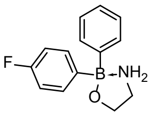

***p*-F-2APB**

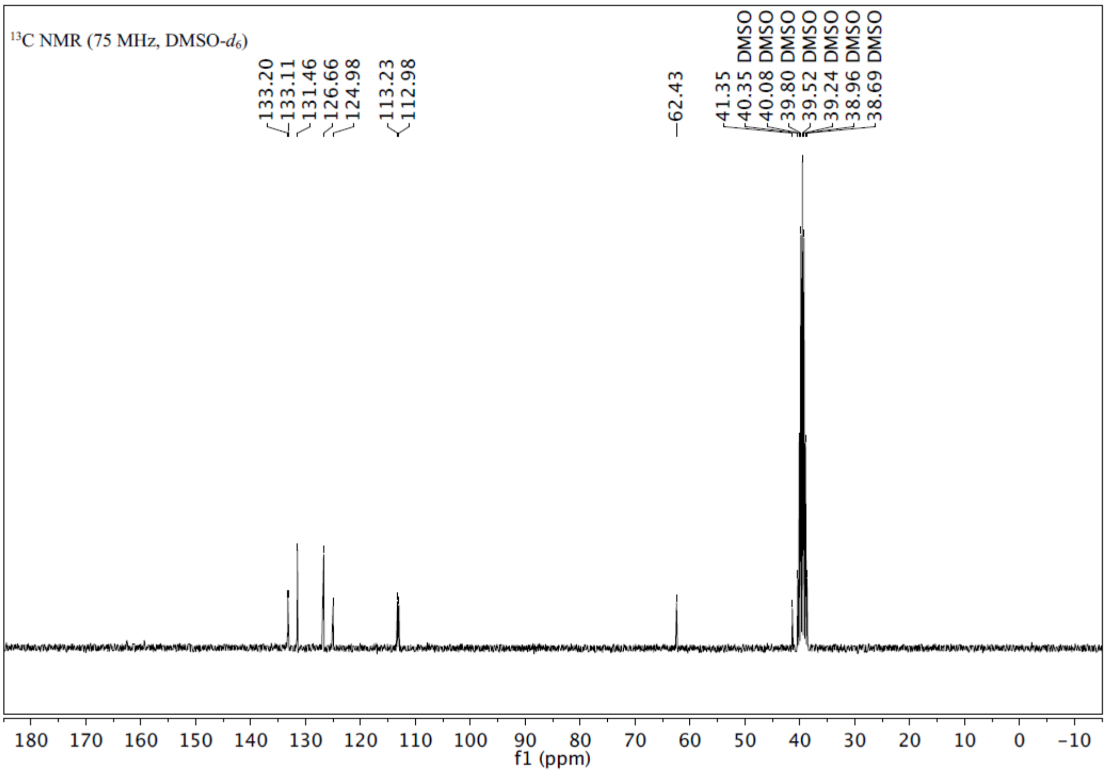

^{13}C NMR (75 MHz, DMSO-d_6)
133.20
133.11
131.46
126.66
124.98
113.23
112.98
62.43
41.35
40.35 DMSO
40.08 DMSO
39.80 DMSO
39.52 DMSO
39.24 DMSO
38.96 DMSO
38.69 DMSO
f1 (ppm)

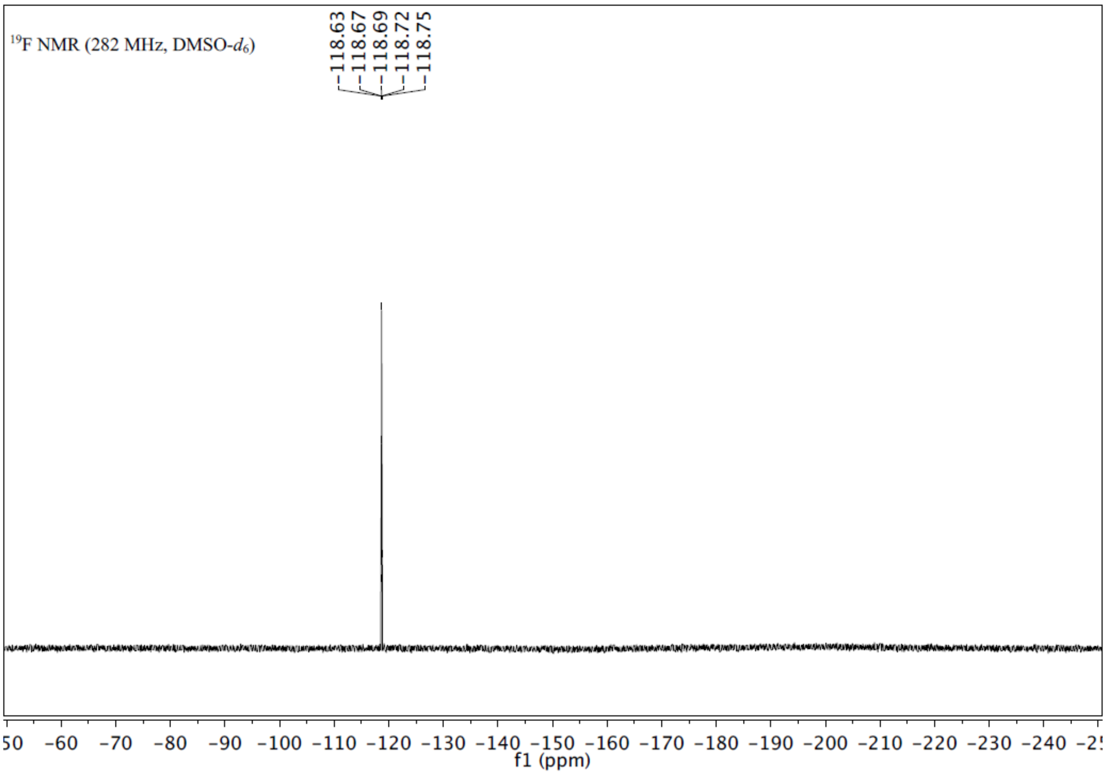

^{19}F NMR (282 MHz, DMSO-d_6)
-118.63
-118.67
-118.69
-118.72
-118.75
f1 (ppm)

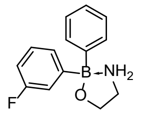

F
B
NH2
O

m-F-2APB

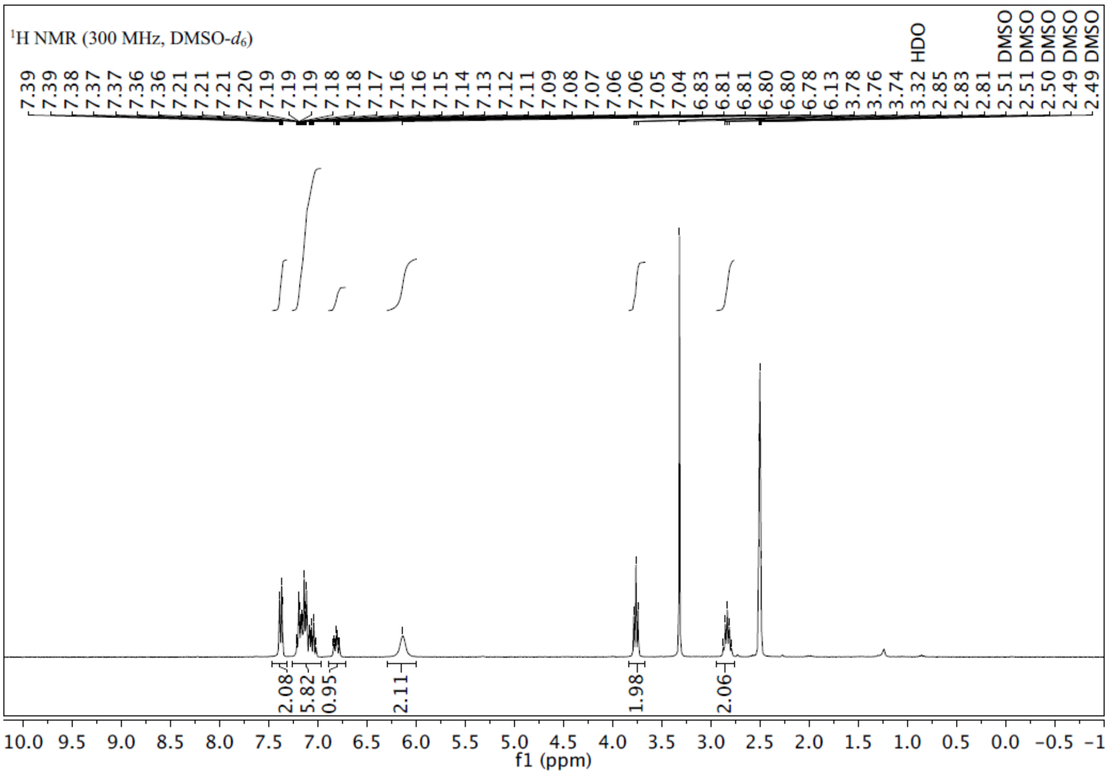

1H NMR (300 MHz, DMSO-d6)
7.39 7.39 7.38 7.37 7.37 7.36 7.36 7.21 7.21 7.21 7.20 7.19 7.19 7.19 7.18 7.18 7.17 7.16 7.16 7.15 7.14 7.13 7.12 7.11 7.09 7.08 7.07 7.06 7.06 7.05 7.04 6.83 6.81 6.81 6.80 6.80 6.78 6.13 3.78 3.76 3.74 3.32 HDO 2.85 2.83 2.81 2.51 DMSO 2.51 DMSO 2.50 DMSO 2.49 DMSO 2.49 DMSO
2.08 5.82 0.95 2.11 1.98 2.06
10.0 9.5 9.0 8.5 8.0 7.5 7.0 6.5 6.0 5.5 5.0 4.5 4.0 3.5 3.0 2.5 2.0 1.5 1.0 0.5 0.0 −0.5 −1
f1 (ppm)

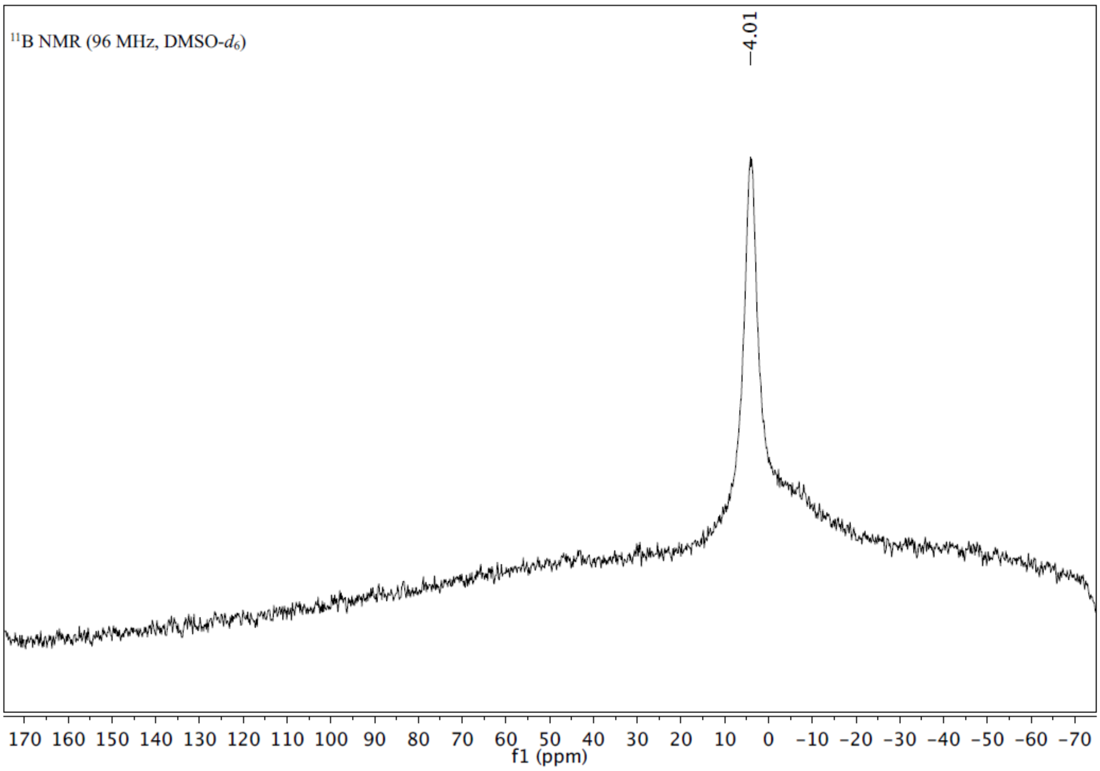

11B NMR (96 MHz, DMSO-d6)
−4.01
170 160 150 140 130 120 110 100 90 80 70 60 50 40 30 20 10 0 −10 −20 −30 −40 −50 −60 −70
f1 (ppm)

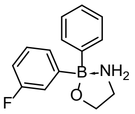
F
B
NH2
O

***m*-F-2APB**

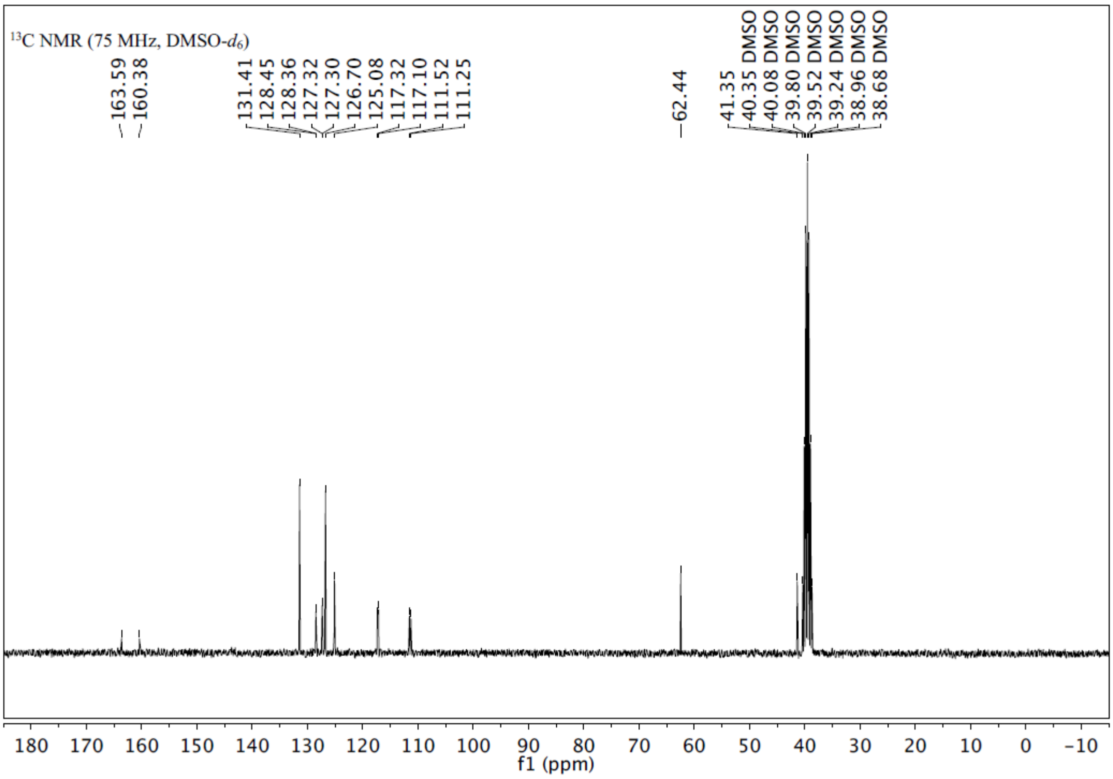
13C NMR (75 MHz, DMSO-d6)
163.59
160.38
131.41
128.45
128.36
127.32
127.30
126.70
125.08
117.32
117.10
111.52
111.25
62.44
41.35
40.35 DMSO
40.08 DMSO
39.80 DMSO
39.52 DMSO
39.24 DMSO
38.96 DMSO
38.68 DMSO
f1 (ppm)

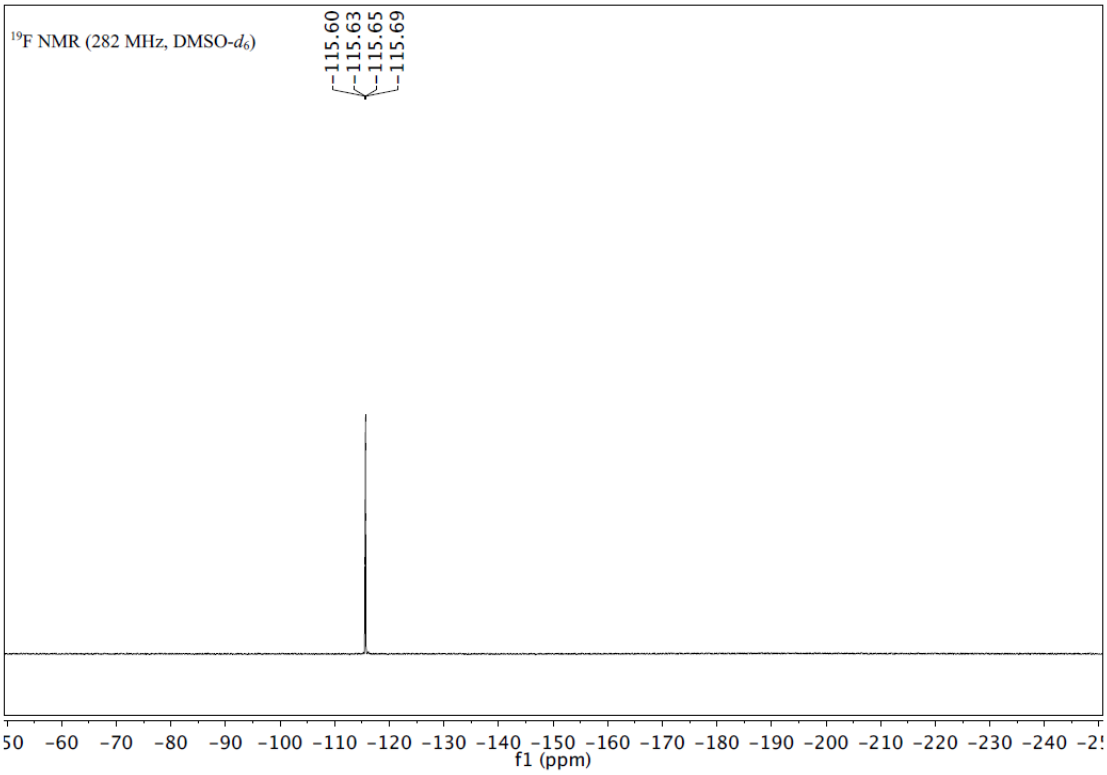
19F NMR (282 MHz, DMSO-d6)
-115.60
-115.63
-115.65
-115.69
f1 (ppm)

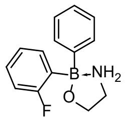

B
NH2
O
F

o-F-2APB

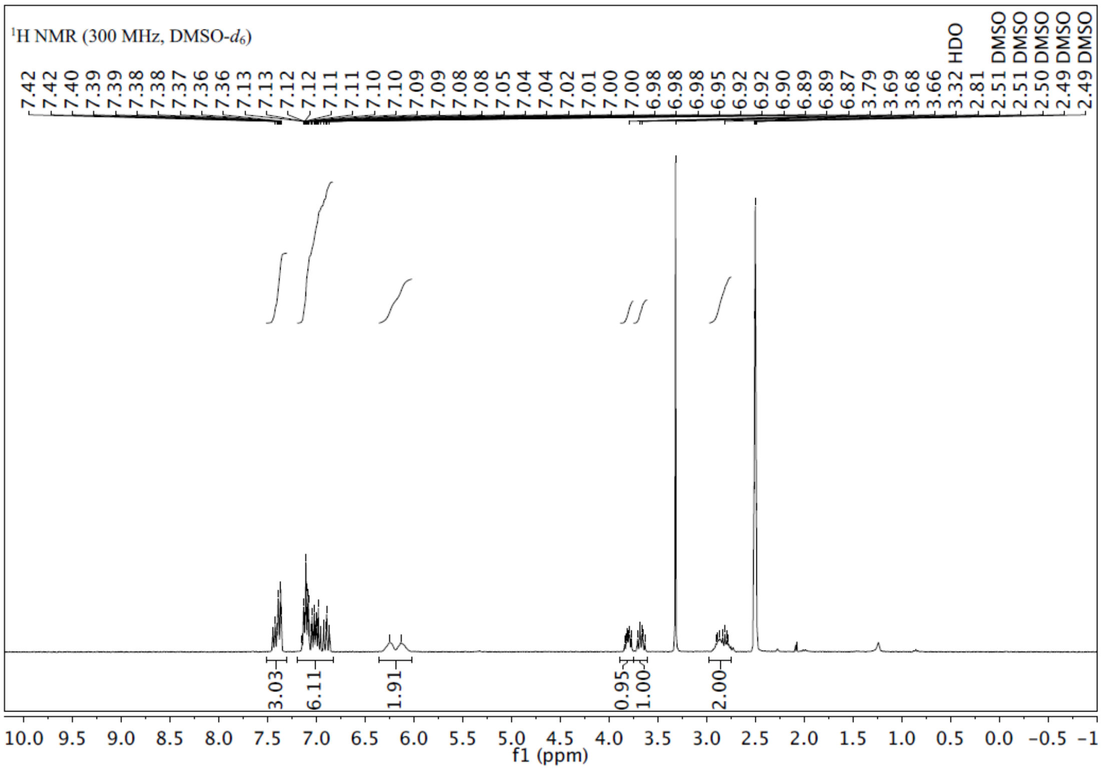

1H NMR (300 MHz, DMSO-d6)
7.42
7.42
7.40
7.39
7.39
7.38
7.38
7.37
7.36
7.36
7.13
7.12
7.12
7.11
7.11
7.10
7.10
7.09
7.09
7.08
7.08
7.05
7.04
7.04
7.02
7.01
7.00
7.00
6.98
6.98
6.98
6.95
6.92
6.92
6.90
6.89
6.89
6.87
3.79
3.69
3.68
3.66
3.32 HDO
2.81
2.51 DMSO
2.51 DMSO
2.50 DMSO
2.49 DMSO
2.49 DMSO
3.03
6.11
1.91
0.95
1.00
2.00
10.0 9.5 9.0 8.5 8.0 7.5 7.0 6.5 6.0 5.5 5.0 4.5 4.0 3.5 3.0 2.5 2.0 1.5 1.0 0.5 0.0 −0.5 −1
f1 (ppm)

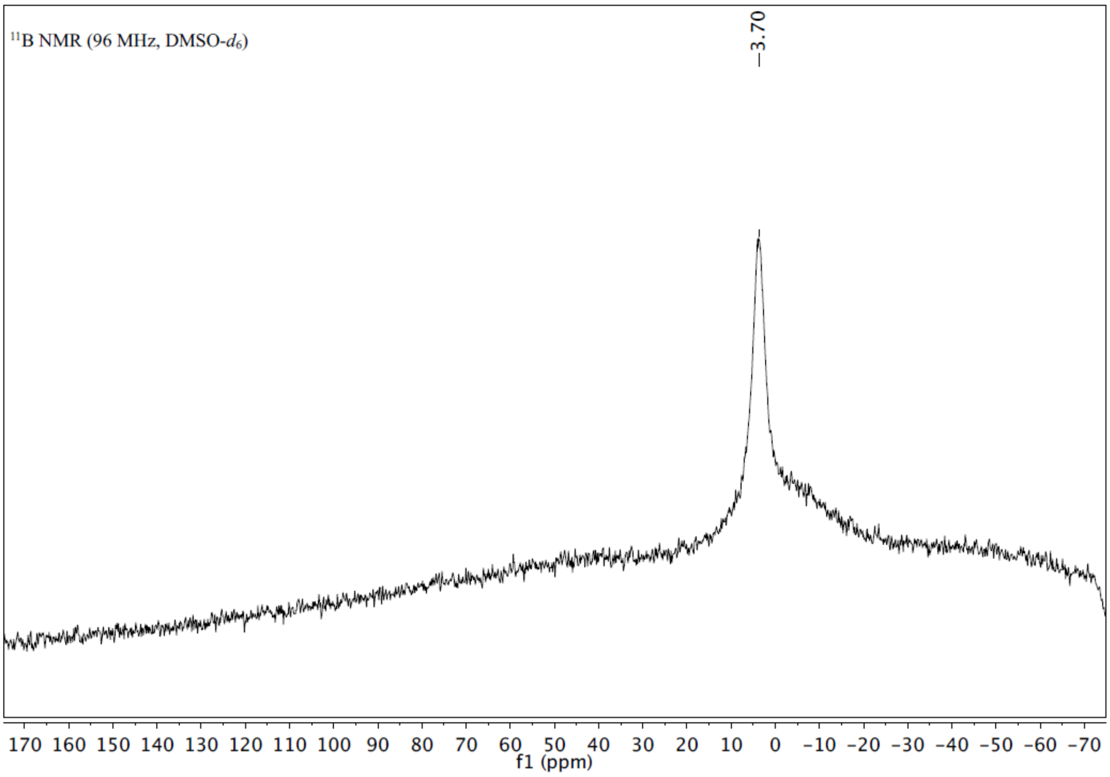

11B NMR (96 MHz, DMSO-d6)
−3.70
170 160 150 140 130 120 110 100 90 80 70 60 50 40 30 20 10 0 −10 −20 −30 −40 −50 −60 −70
f1 (ppm)

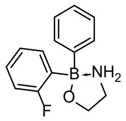
B
NH2
O
F

o-F-2APB

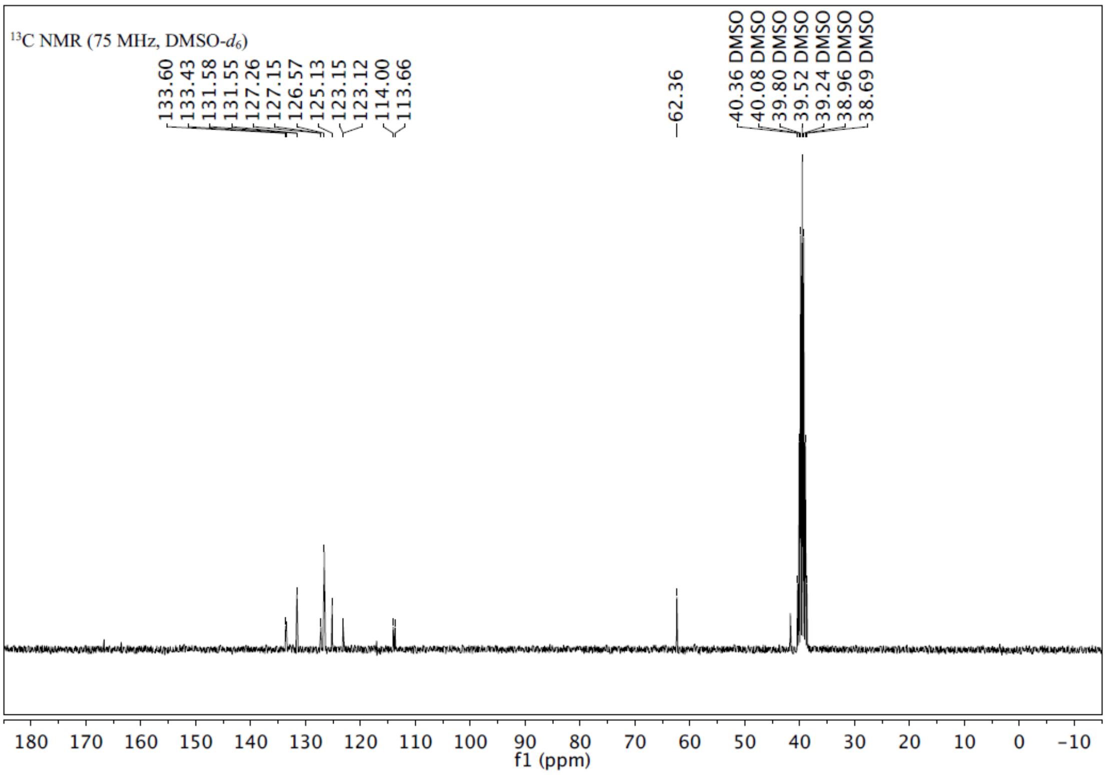
13C NMR (75 MHz, DMSO-d6)
133.60
133.43
131.58
131.55
127.26
127.15
126.57
125.13
123.15
123.12
114.00
113.66
62.36
40.36 DMSO
40.08 DMSO
39.80 DMSO
39.52 DMSO
39.24 DMSO
38.96 DMSO
38.69 DMSO
180 170 160 150 140 130 120 110 100 90 80 70 60 50 40 30 20 10 0 -10
f1 (ppm)

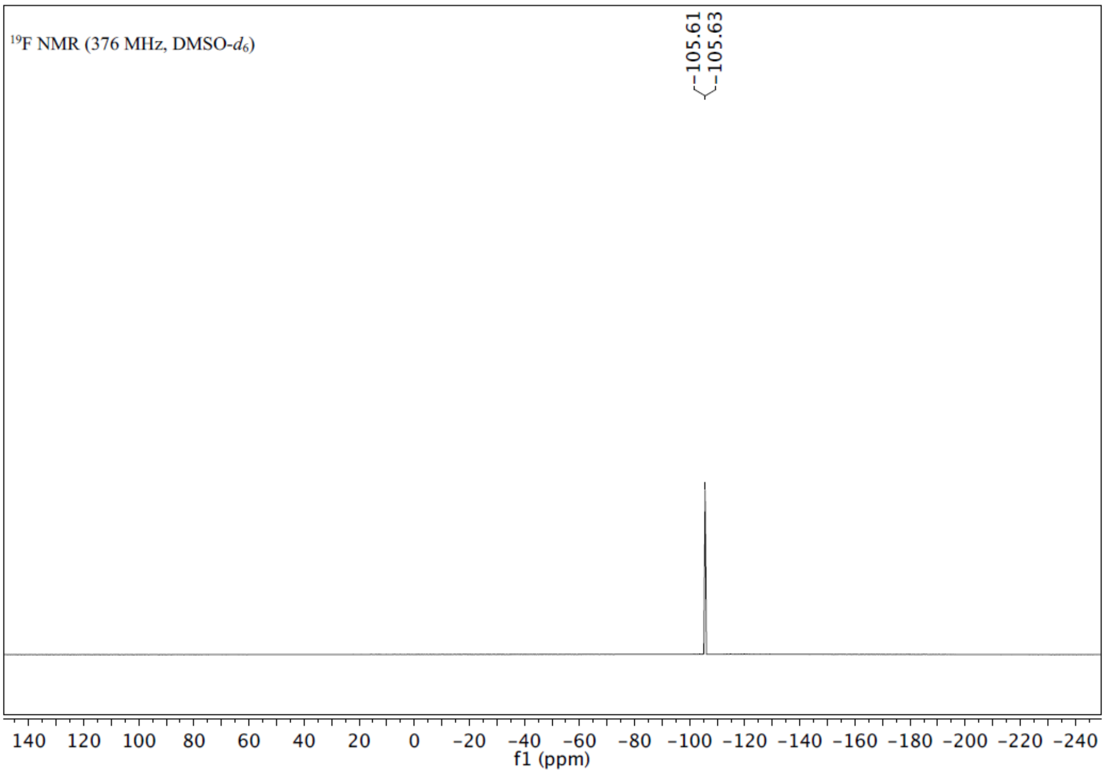
19F NMR (376 MHz, DMSO-d6)
-105.61
-105.63
140 120 100 80 60 40 20 0 -20 -40 -60 -80 -100 -120 -140 -160 -180 -200 -220 -240
f1 (ppm)

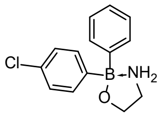
Cl
B
NH2
O

***p*-Cl-2APB**

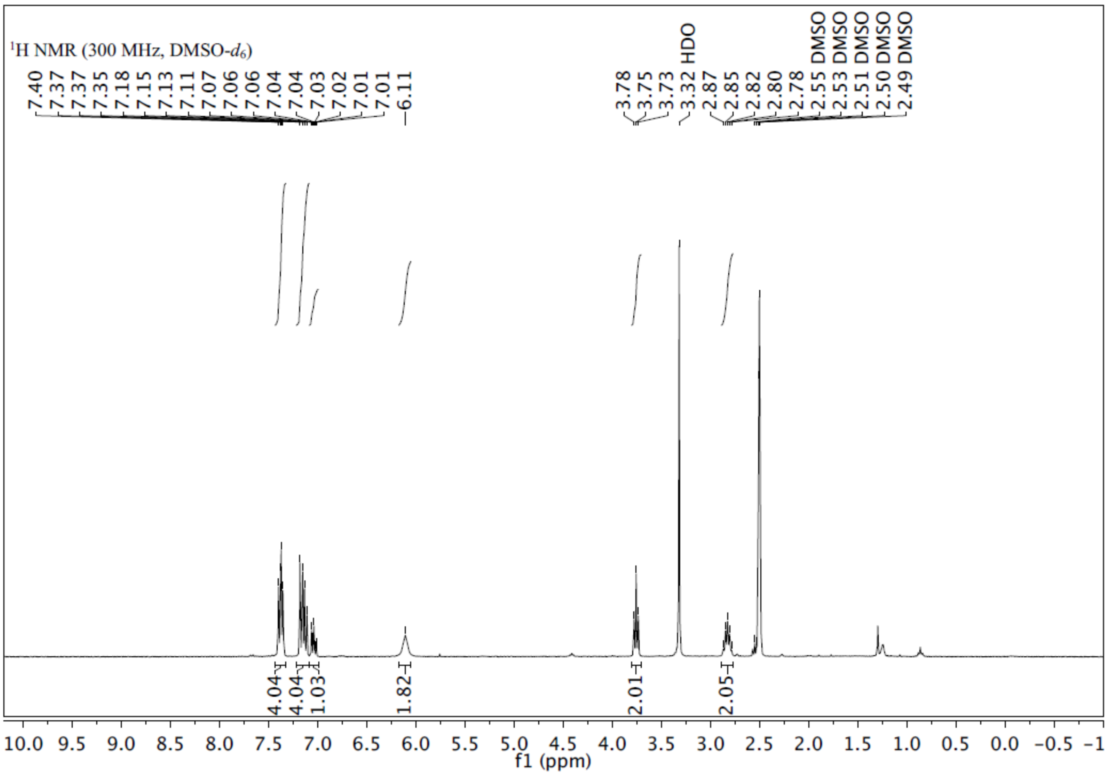
1H NMR (300 MHz, DMSO-d6)
7.40
7.37
7.37
7.35
7.18
7.15
7.13
7.11
7.07
7.06
7.06
7.04
7.04
7.03
7.02
7.01
7.01
6.11
3.78
3.75
3.73
3.32 HDO
2.87
2.85
2.82
2.80
2.78
2.55 DMSO
2.53 DMSO
2.51 DMSO
2.50 DMSO
2.49 DMSO
4.04
4.04
1.03
1.82
2.01
2.05
f1 (ppm)

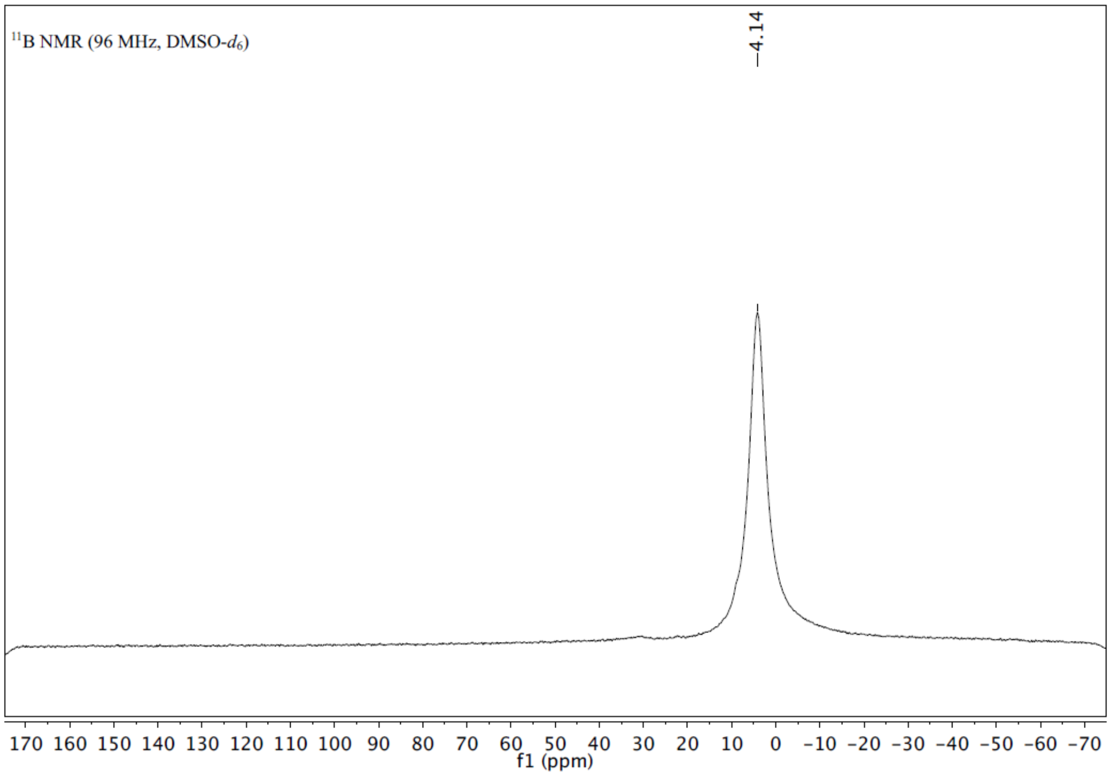
11B NMR (96 MHz, DMSO-d6)
4.14
f1 (ppm)

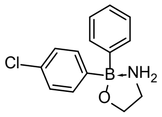

***p*-Cl-2APB**

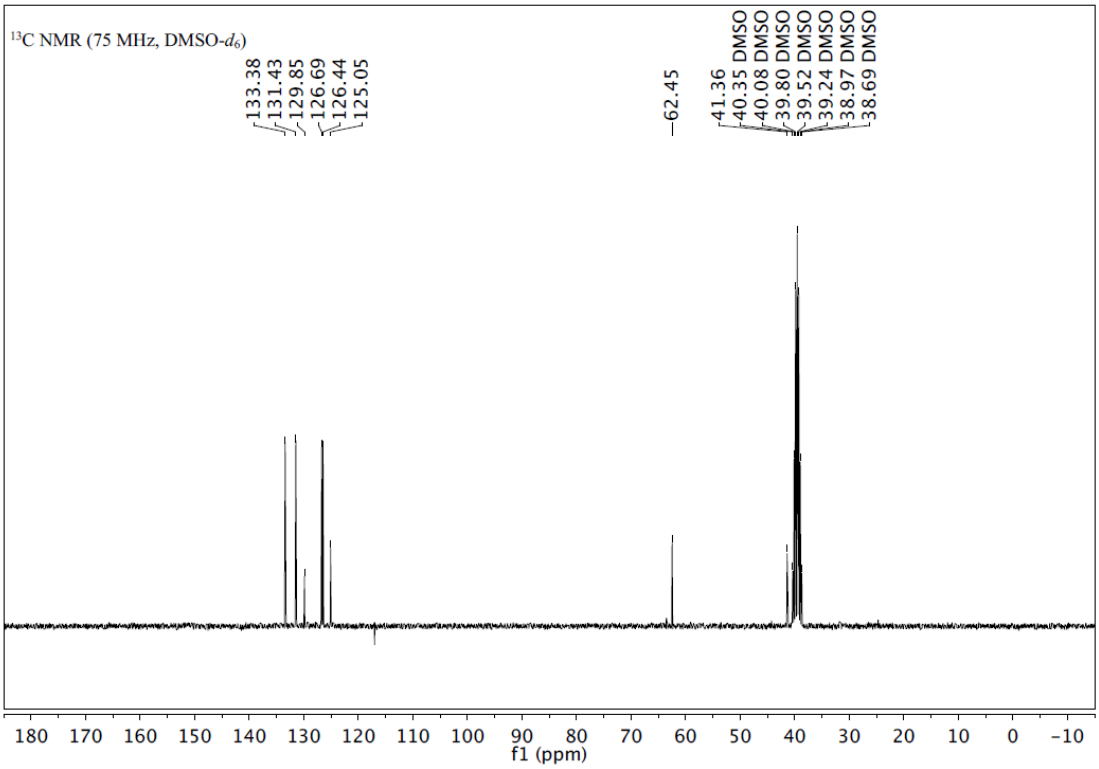

^{13}C NMR (75 MHz, DMSO-d_6)
133.38
131.43
129.85
126.69
126.44
125.05
62.45
41.36
40.35 DMSO
40.08 DMSO
39.80 DMSO
39.52 DMSO
39.24 DMSO
38.97 DMSO
38.69 DMSO
180 170 160 150 140 130 120 110 100 90 80 70 60 50 40 30 20 10 0 −10
f1 (ppm)

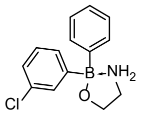
Cl
B
NH2
O

***m*-Cl-2APB**

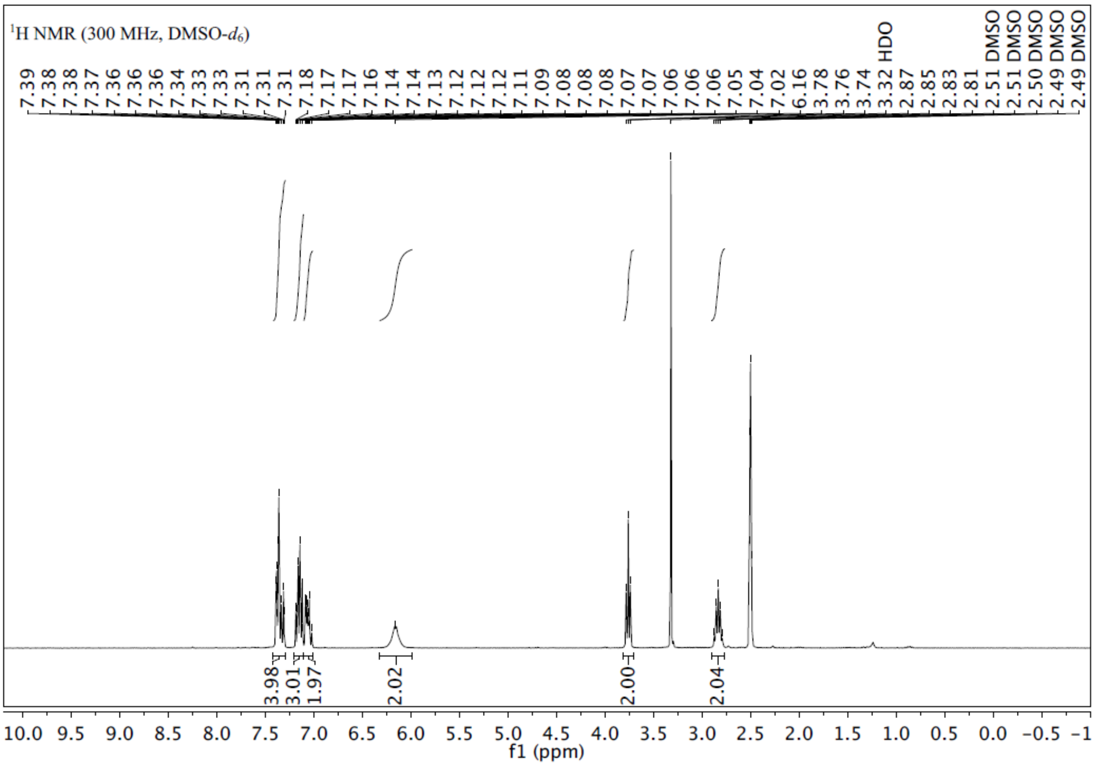
1H NMR (300 MHz, DMSO-d6)
7.39
7.38
7.38
7.37
7.36
7.36
7.36
7.34
7.33
7.33
7.31
7.31
7.31
7.18
7.17
7.17
7.16
7.14
7.14
7.13
7.12
7.12
7.12
7.11
7.09
7.08
7.08
7.08
7.07
7.07
7.06
7.06
7.06
7.05
7.04
7.02
6.16
3.78
3.76
3.74
3.32 HDO
2.87
2.85
2.83
2.81
2.51 DMSO
2.51 DMSO
2.50 DMSO
2.49 DMSO
2.49 DMSO
3.98
3.01
1.97
2.02
2.00
2.04
10.0 9.5 9.0 8.5 8.0 7.5 7.0 6.5 6.0 5.5 5.0 4.5 4.0 3.5 3.0 2.5 2.0 1.5 1.0 0.5 0.0 −0.5 −1
f1 (ppm)

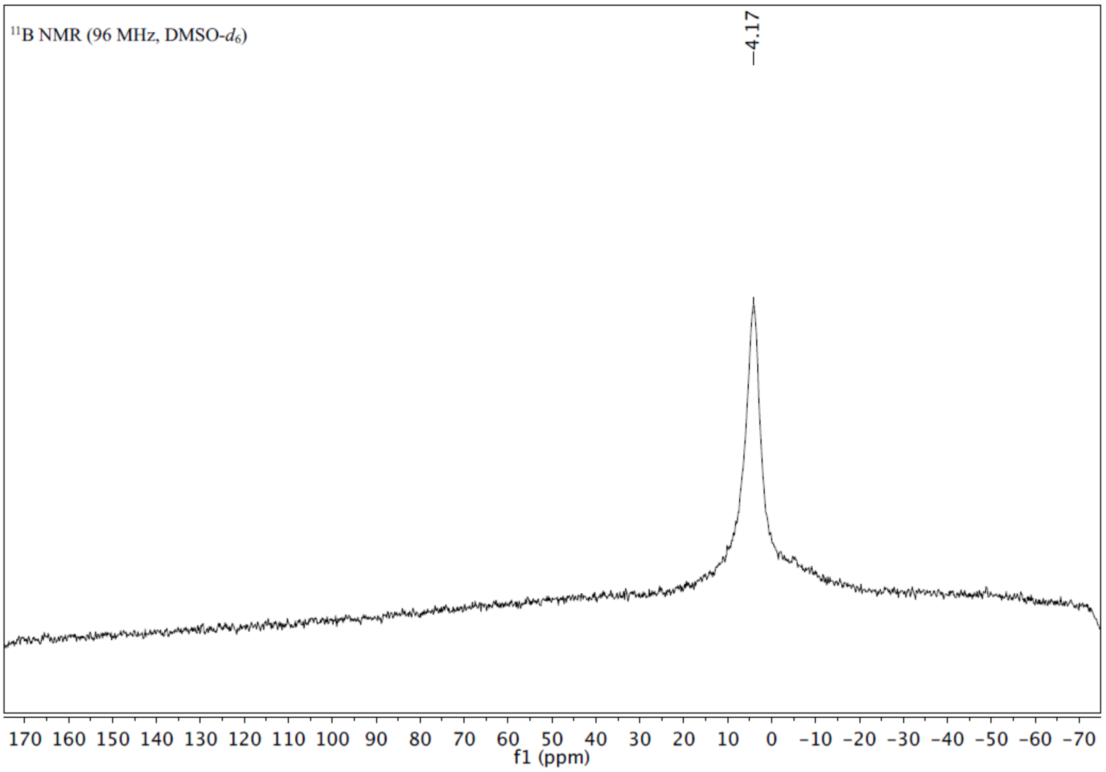
11B NMR (96 MHz, DMSO-d6)
4.17
170 160 150 140 130 120 110 100 90 80 70 60 50 40 30 20 10 0 −10 −20 −30 −40 −50 −60 −70
f1 (ppm)

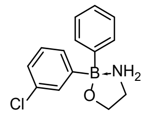

***m*-Cl-2APB**

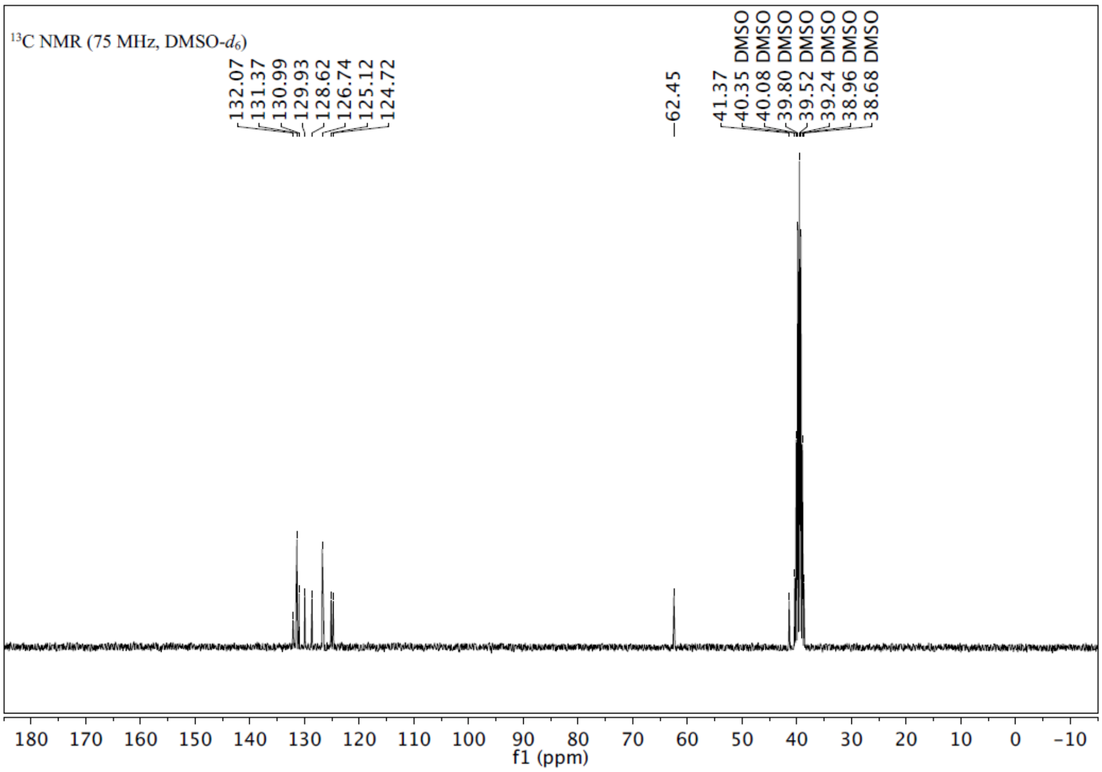

13C NMR (75 MHz, DMSO-d_6)
132.07
131.37
130.99
129.93
128.62
126.74
125.12
124.72
62.45
41.37
40.35 DMSO
40.08 DMSO
39.80 DMSO
39.52 DMSO
39.24 DMSO
38.96 DMSO
38.68 DMSO
180 170 160 150 140 130 120 110 100 90 80 70 60 50 40 30 20 10 0 −10
f1 (ppm)

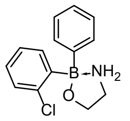
B
NH2
O
Cl

***o*-Cl-2APB**

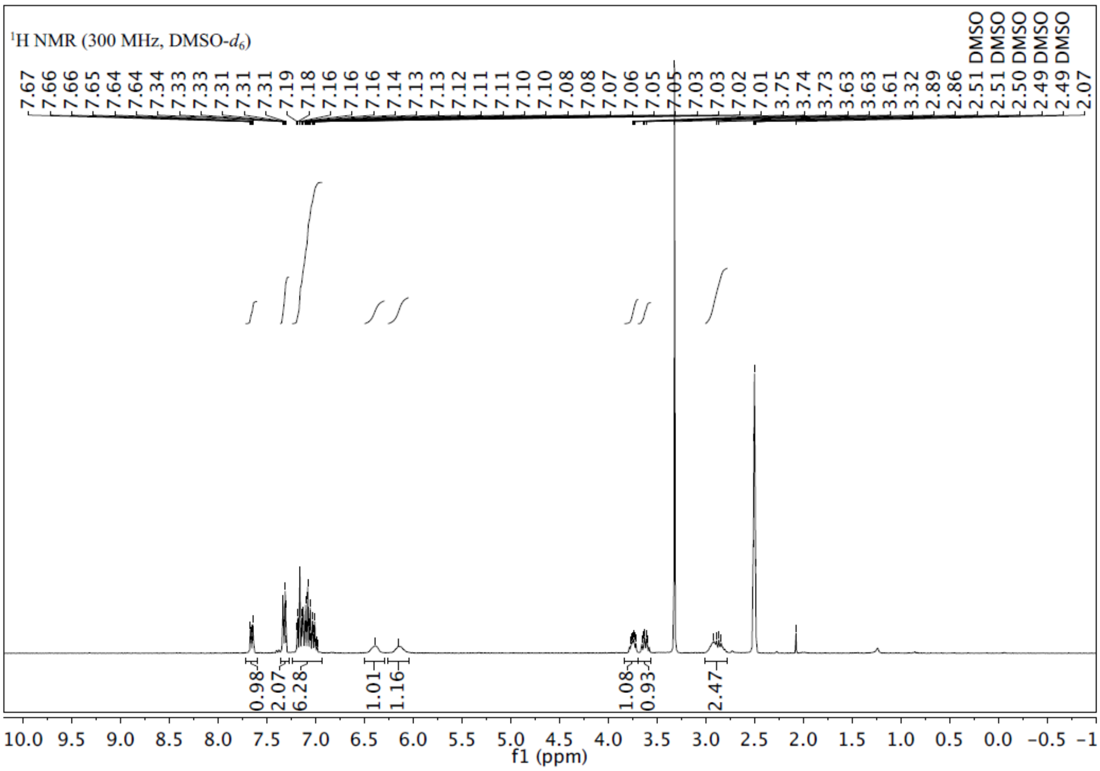
1H NMR (300 MHz, DMSO-d6)
7.67 7.66 7.66 7.65 7.64 7.64 7.34 7.33 7.33 7.31 7.31 7.31 7.19 7.18 7.16 7.16 7.16 7.14 7.13 7.13 7.12 7.11 7.11 7.10 7.10 7.08 7.08 7.07 7.06 7.05 7.05 7.03 7.02 7.01 3.75 3.74 3.73 3.63 3.63 3.61 3.32 2.89 2.86 2.51 DMSO 2.51 DMSO 2.50 DMSO 2.49 DMSO 2.49 DMSO 2.07
0.98 2.07 6.28 1.01 1.16 1.08 0.93 2.47
10.0 9.5 9.0 8.5 8.0 7.5 7.0 6.5 6.0 5.5 5.0 4.5 4.0 3.5 3.0 2.5 2.0 1.5 1.0 0.5 0.0 −0.5 −1
f1 (ppm)

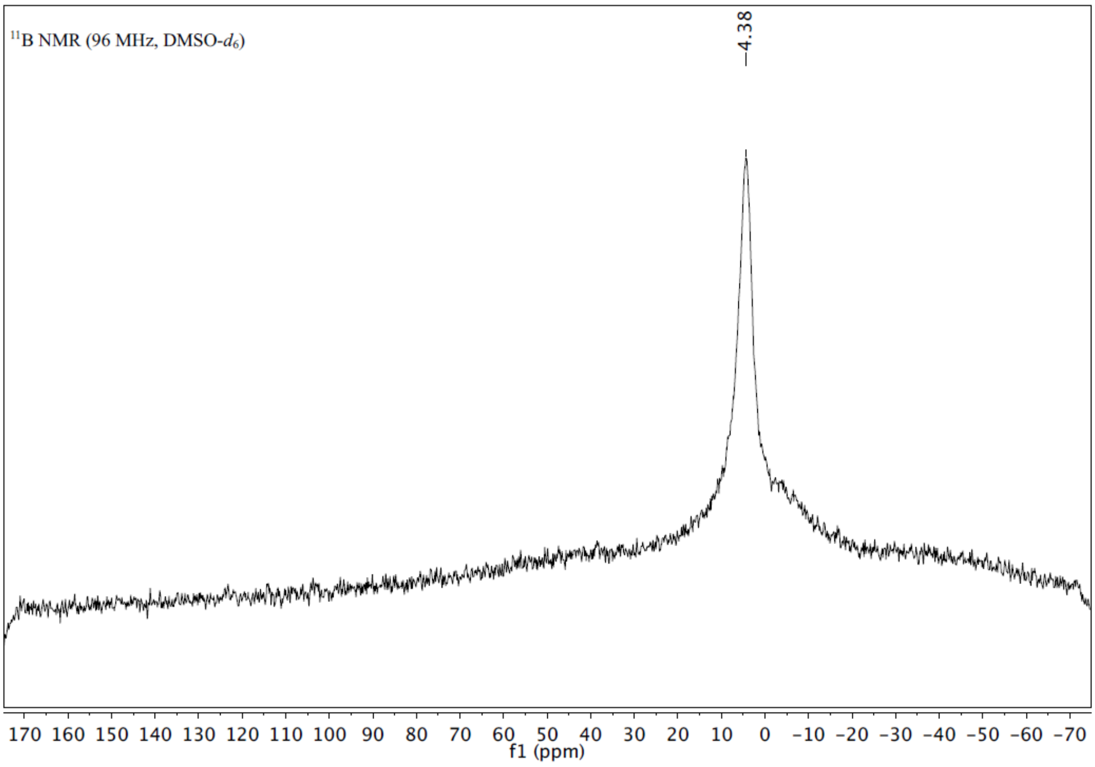
11B NMR (96 MHz, DMSO-d6)
4.38
170 160 150 140 130 120 110 100 90 80 70 60 50 40 30 20 10 0 −10 −20 −30 −40 −50 −60 −70
f1 (ppm)

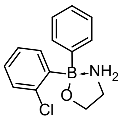
NH2
B
O
Cl

***o*-Cl-2APB**

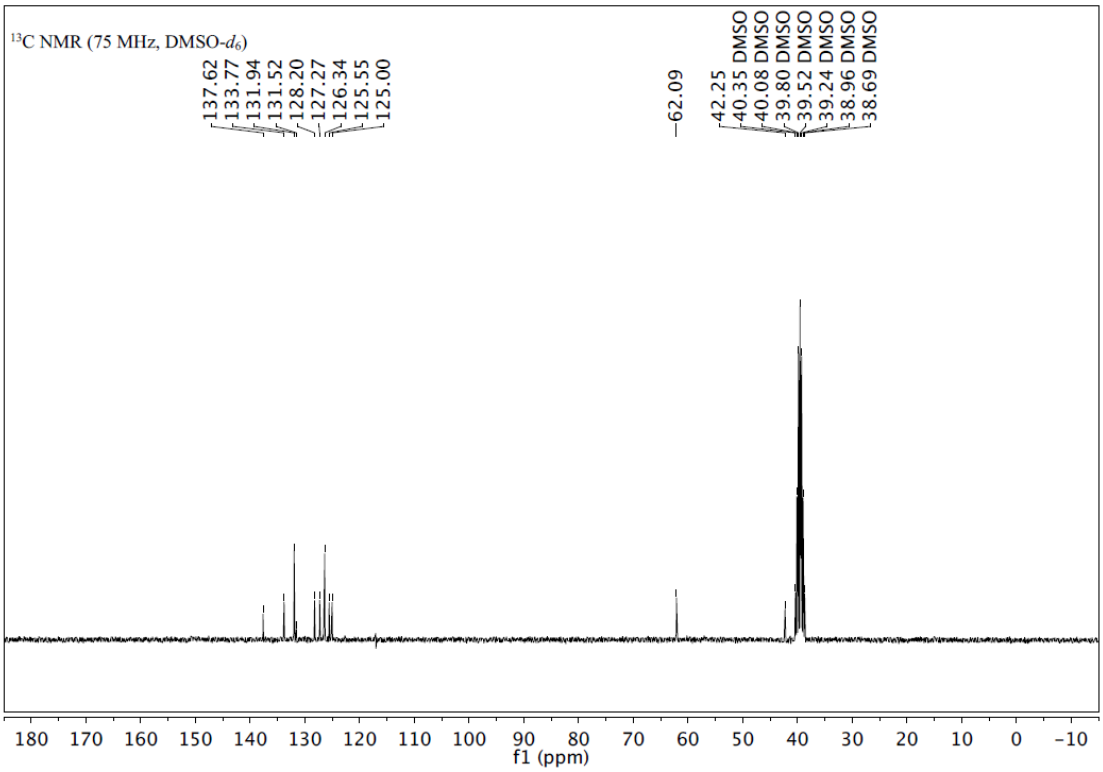
13C NMR (75 MHz, DMSO-d6)
137.62
133.77
131.94
131.52
128.20
127.27
126.34
125.55
125.00
62.09
42.25
40.35 DMSO
40.08 DMSO
39.80 DMSO
39.52 DMSO
39.24 DMSO
38.96 DMSO
38.69 DMSO
180 170 160 150 140 130 120 110 100 90 80 70 60 50 40 30 20 10 0 −10
f1 (ppm)

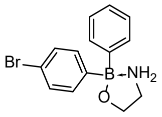
Br
B
NH2
O

***p*-Br-2APB**

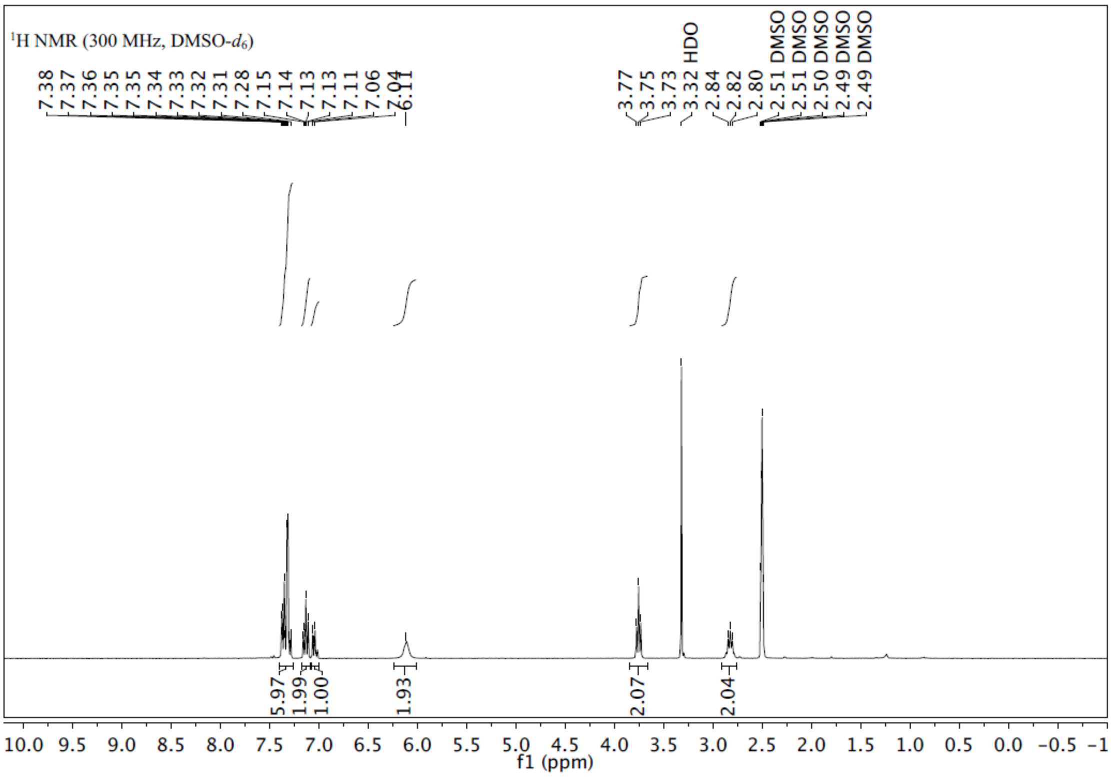
1H NMR (300 MHz, DMSO-d6)
7.38
7.37
7.36
7.35
7.35
7.34
7.33
7.32
7.31
7.28
7.15
7.14
7.13
7.13
7.11
7.06
7.04
6.11
3.77
3.75
3.73
3.32 HDO
2.84
2.82
2.80
2.51 DMSO
2.51 DMSO
2.50 DMSO
2.49 DMSO
2.49 DMSO
5.97
1.99
1.00
1.93
2.07
2.04
10.0 9.5 9.0 8.5 8.0 7.5 7.0 6.5 6.0 5.5 5.0 4.5 4.0 3.5 3.0 2.5 2.0 1.5 1.0 0.5 0.0 −0.5 −1
f1 (ppm)

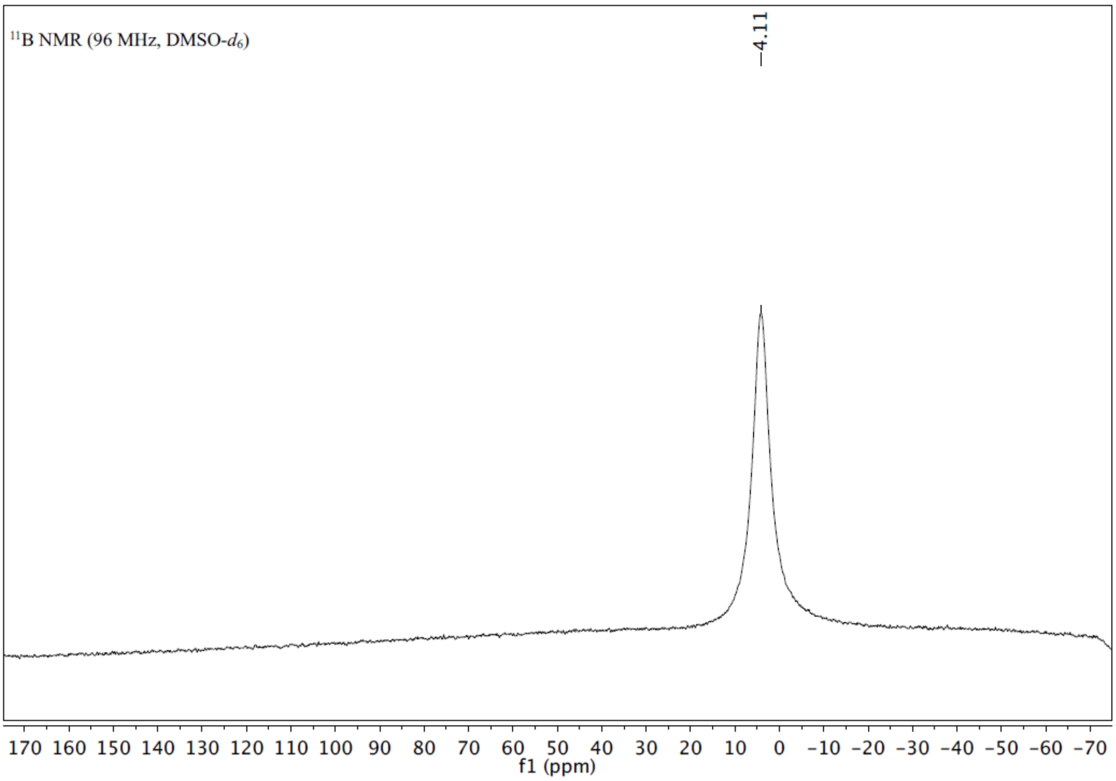
11B NMR (96 MHz, DMSO-d6)
4.11
170 160 150 140 130 120 110 100 90 80 70 60 50 40 30 20 10 0 −10 −20 −30 −40 −50 −60 −70
f1 (ppm)

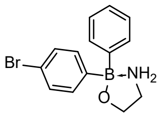

Br
B
NH2
O

***p*-Br-2APB**

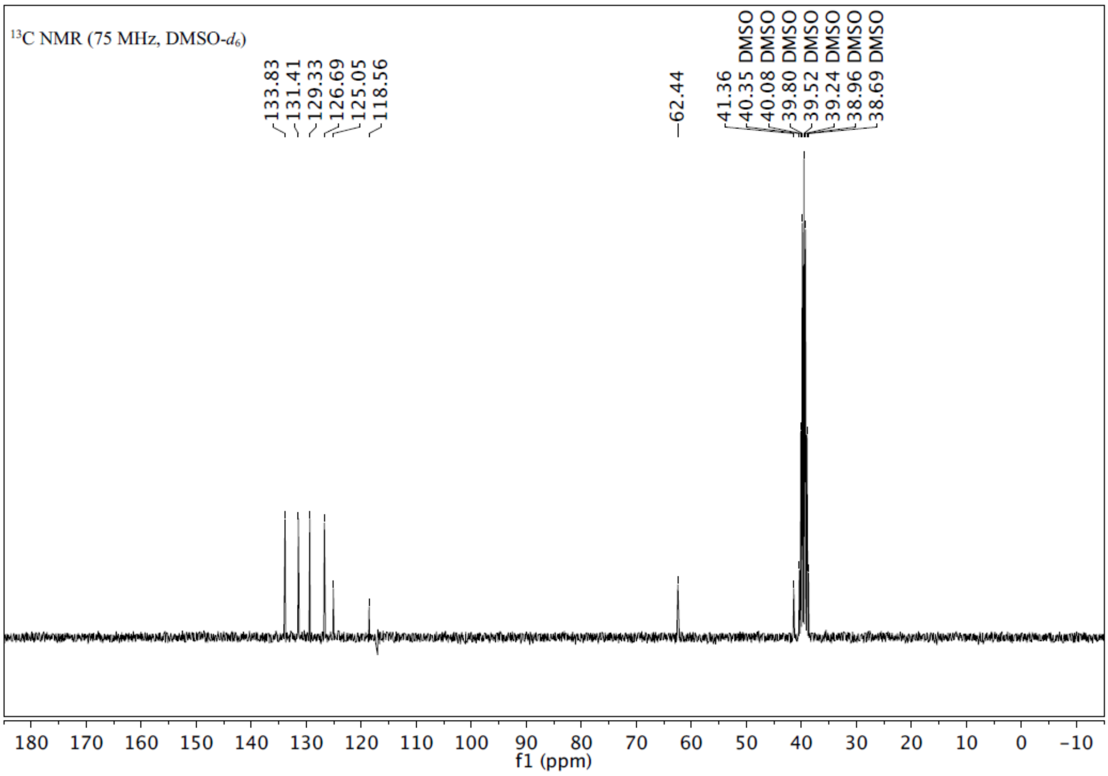

13C NMR (75 MHz, DMSO-d6)
133.83
131.41
129.33
126.69
125.05
118.56
62.44
41.36
40.35 DMSO
40.08 DMSO
39.80 DMSO
39.52 DMSO
39.24 DMSO
38.96 DMSO
38.69 DMSO
180 170 160 150 140 130 120 110 100 90 80 70 60 50 40 30 20 10 0 −10
f1 (ppm)

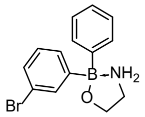

***m*-Br-2APB**

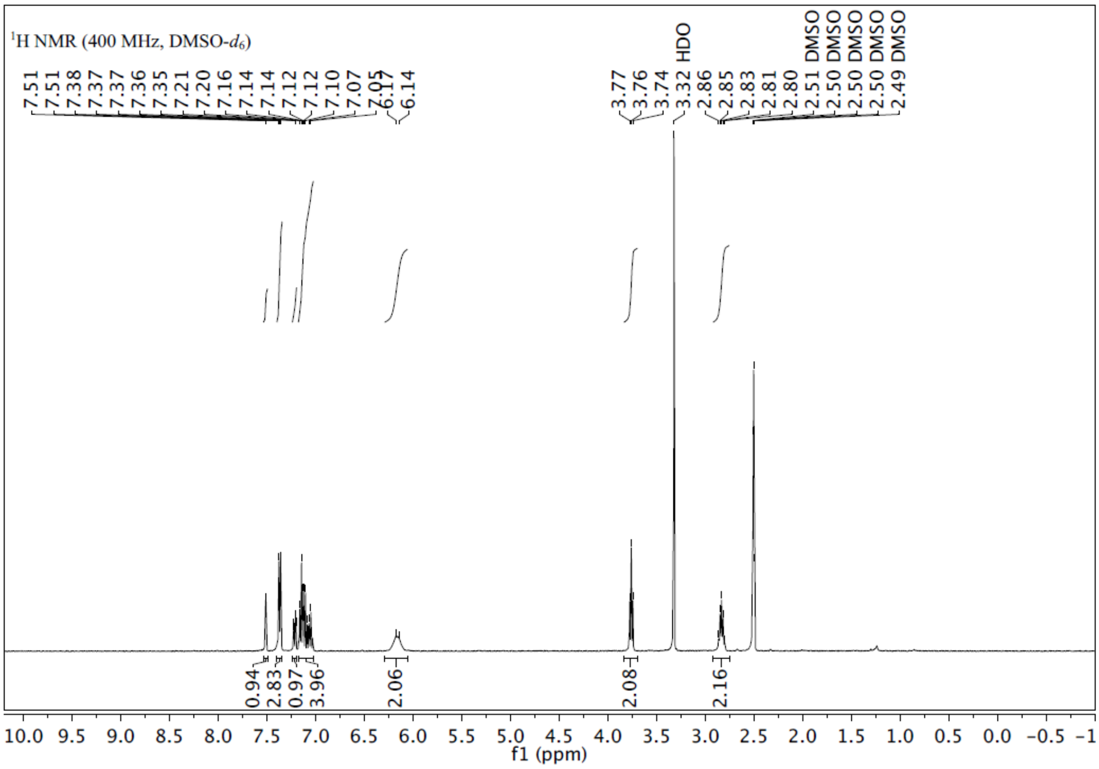

¹H NMR (400 MHz, DMSO-d6)
7.51 7.51 7.38 7.37 7.37 7.36 7.35 7.21 7.20 7.16 7.14 7.14 7.12 7.12 7.10 7.07 7.05 6.17 6.14
3.77 3.76 3.74 3.32 HDO 2.86 2.85 2.83 2.81 2.80 2.51 DMSO 2.50 DMSO 2.50 DMSO 2.50 DMSO 2.49 DMSO
0.94 2.83 0.97 3.96 2.06 2.08 2.16
f1 (ppm)

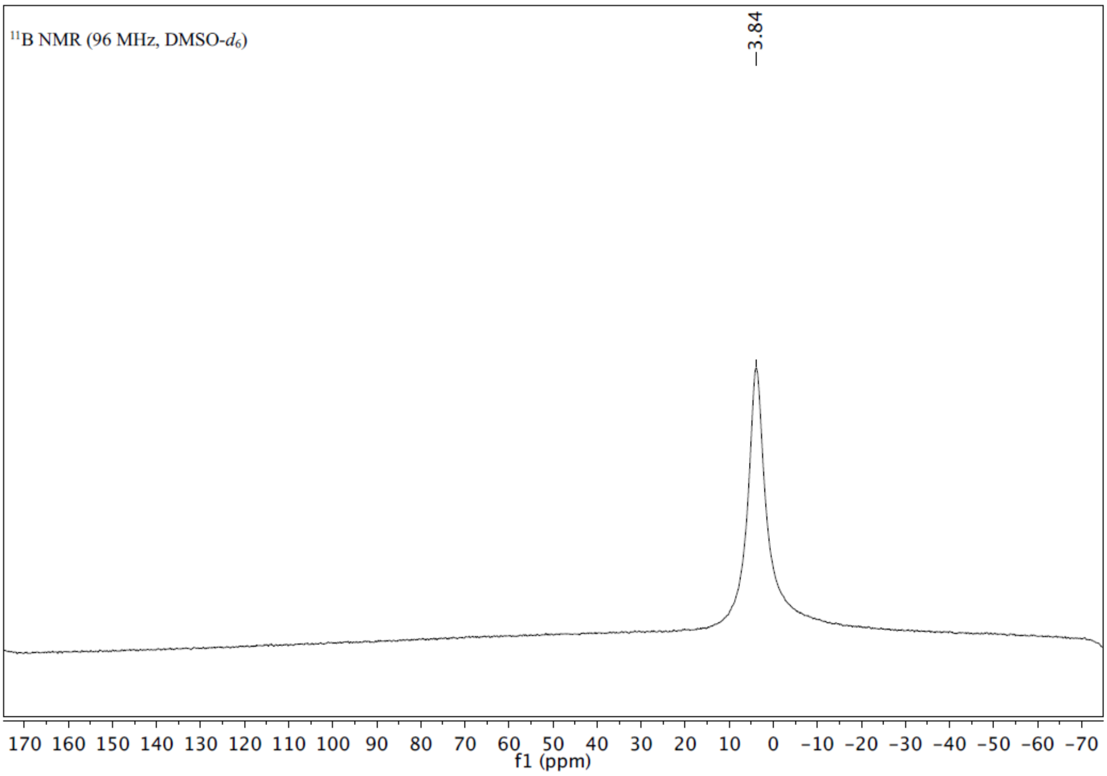

¹¹B NMR (96 MHz, DMSO-d6)
3.84
f1 (ppm)

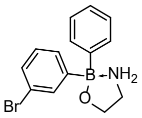
Br
B
NH2
O

***m*-Br-2APB**

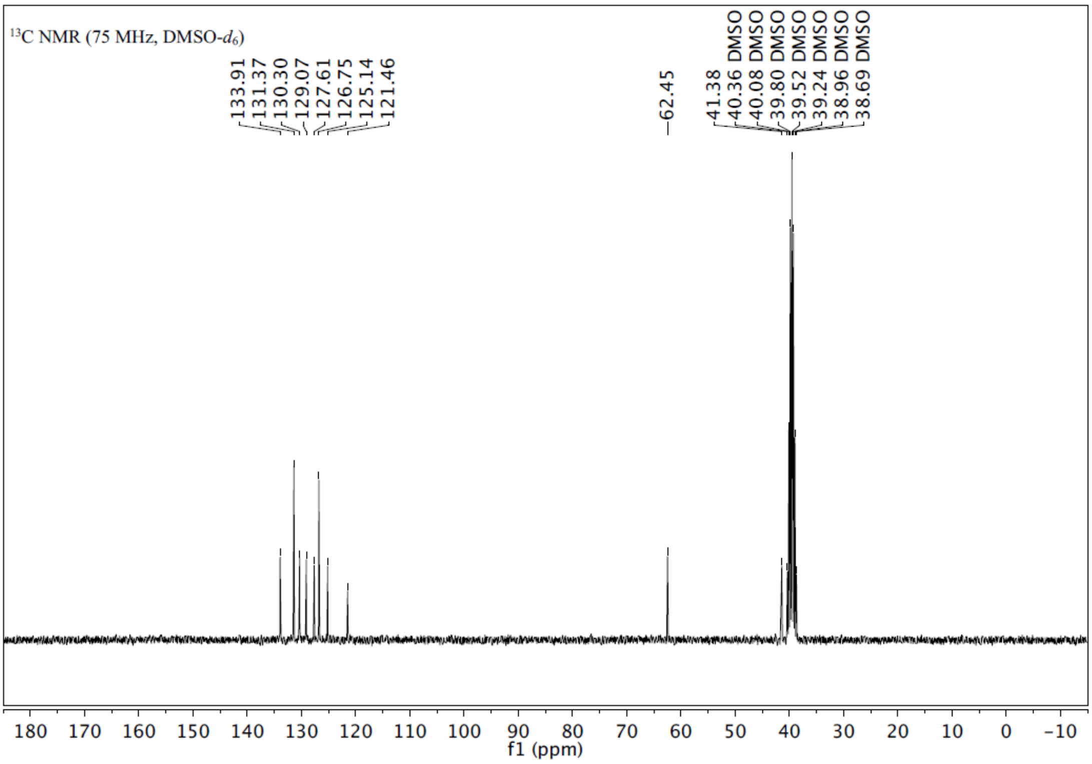
13C NMR (75 MHz, DMSO-d6)
133.91
131.37
130.30
129.07
127.61
126.75
125.14
121.46
62.45
41.38
40.36 DMSO
40.08 DMSO
39.80 DMSO
39.52 DMSO
39.24 DMSO
38.96 DMSO
38.69 DMSO
180 170 160 150 140 130 120 110 100 90 80 70 60 50 40 30 20 10 0 −10
f1 (ppm)

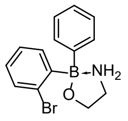
B
NH2
O
Br

o-Br-2APB

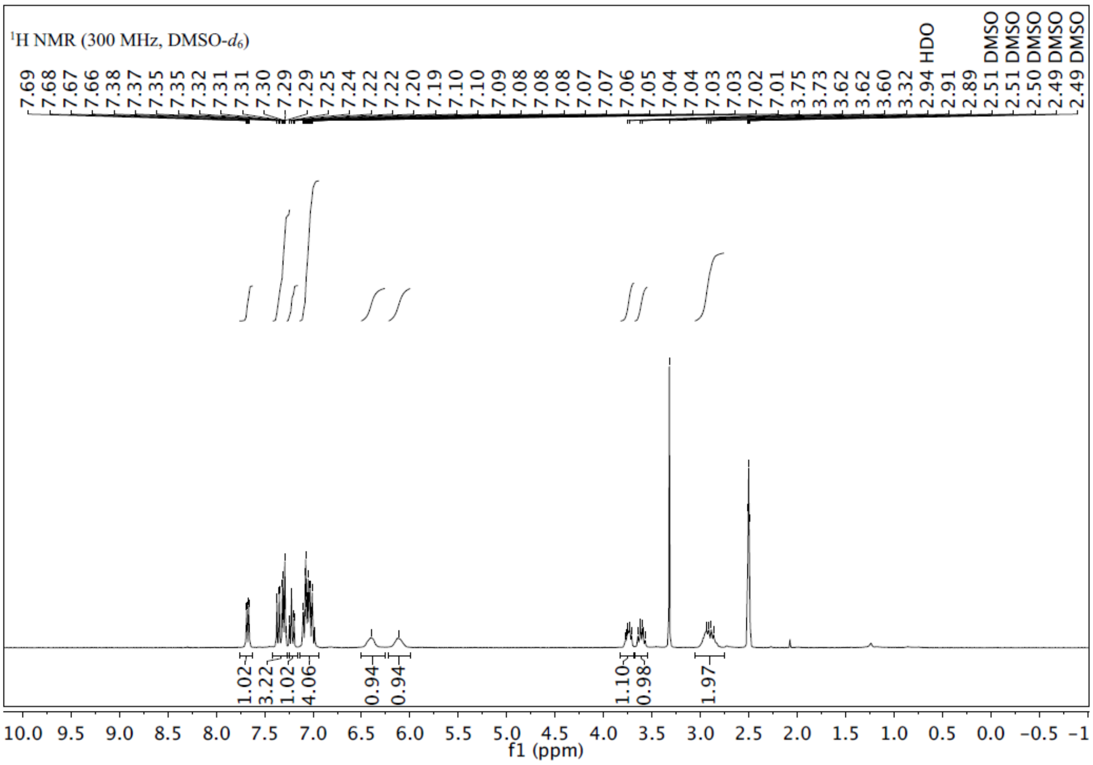
1H NMR (300 MHz, DMSO-d6)
7.69
7.68
7.67
7.66
7.38
7.37
7.35
7.35
7.32
7.31
7.31
7.30
7.29
7.29
7.25
7.24
7.22
7.22
7.20
7.19
7.10
7.10
7.09
7.08
7.08
7.07
7.07
7.06
7.05
7.04
7.04
7.03
7.03
7.02
7.01
3.75
3.73
3.62
3.62
3.60
3.32 HDO
2.94
2.91
2.89
2.51 DMSO
2.51 DMSO
2.50 DMSO
2.49 DMSO
2.49 DMSO
1.02
3.22
1.02
4.06
0.94
0.94
1.10
0.98
1.97
10.0 9.5 9.0 8.5 8.0 7.5 7.0 6.5 6.0 5.5 5.0 4.5 4.0 3.5 3.0 2.5 2.0 1.5 1.0 0.5 0.0 −0.5 −1
f1 (ppm)

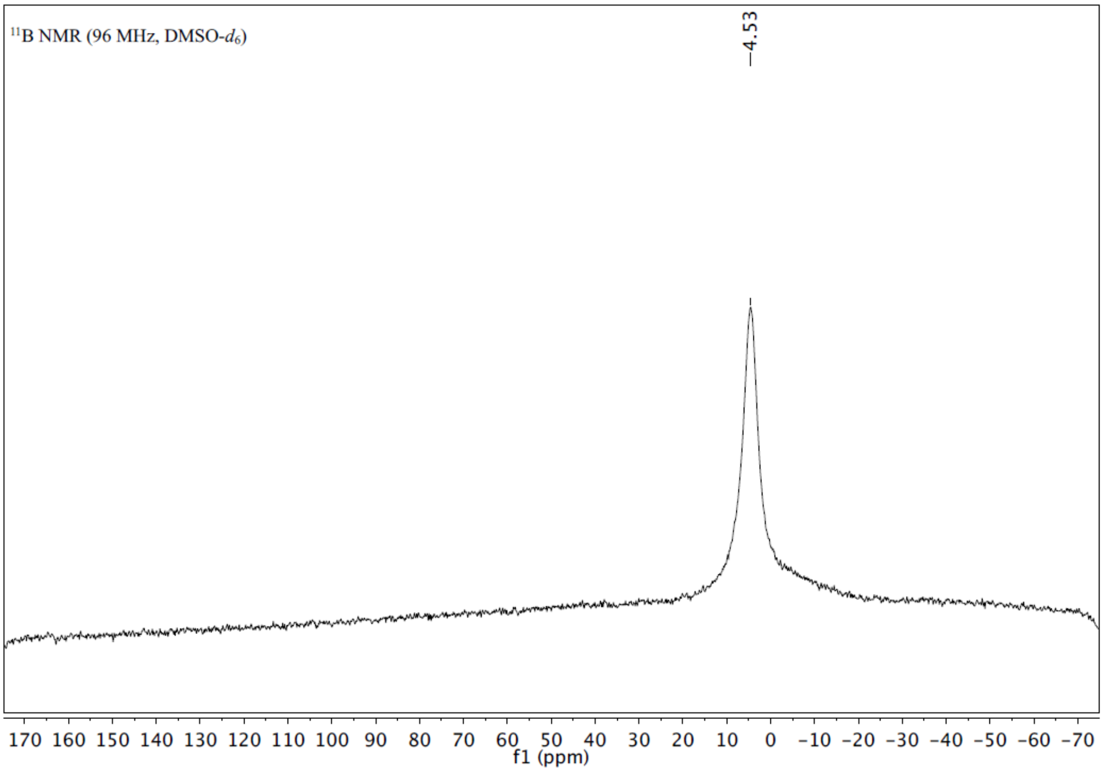
11B NMR (96 MHz, DMSO-d6)
4.53
170 160 150 140 130 120 110 100 90 80 70 60 50 40 30 20 10 0 −10 −20 −30 −40 −50 −60 −70
f1 (ppm)

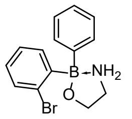
B
NH2
O
Br

***o*-Br-2APB**

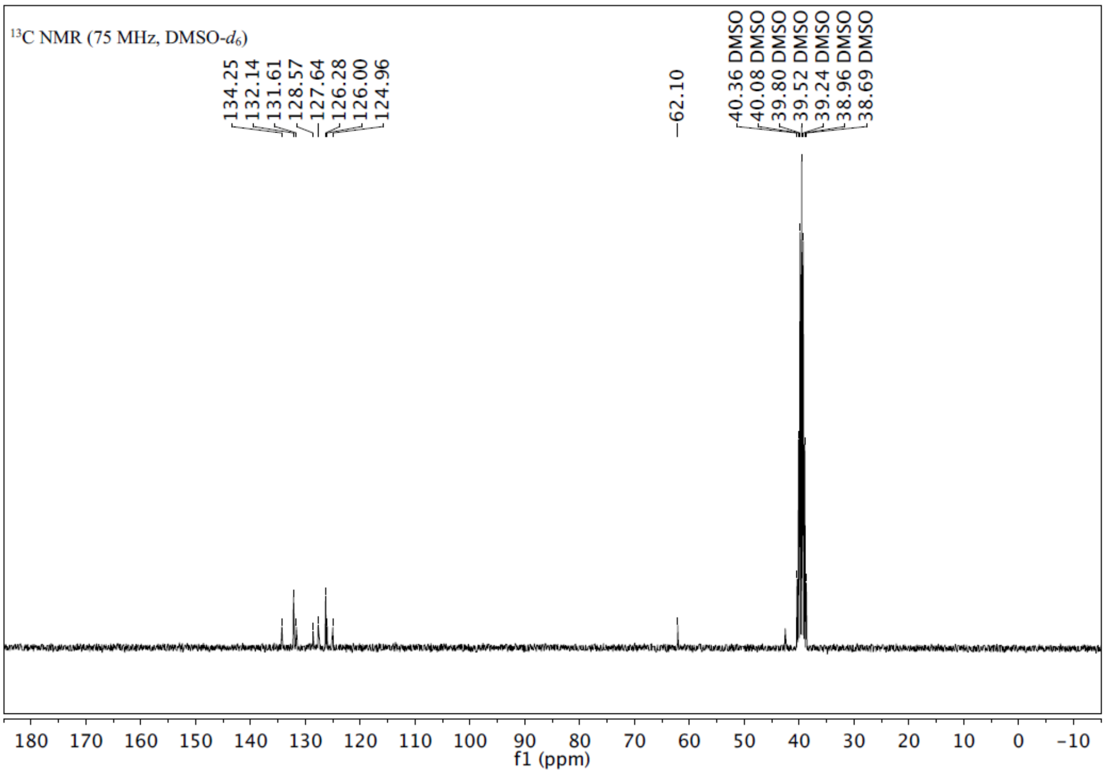
13C NMR (75 MHz, DMSO-d6)
134.25
132.14
131.61
128.57
127.64
126.28
126.00
124.96
62.10
40.36 DMSO
40.08 DMSO
39.80 DMSO
39.52 DMSO
39.24 DMSO
38.96 DMSO
38.69 DMSO
180 170 160 150 140 130 120 110 100 90 80 70 60 50 40 30 20 10 0 −10
f1 (ppm)

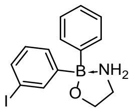
B
NH2
O
I

***m*-I-2APB**

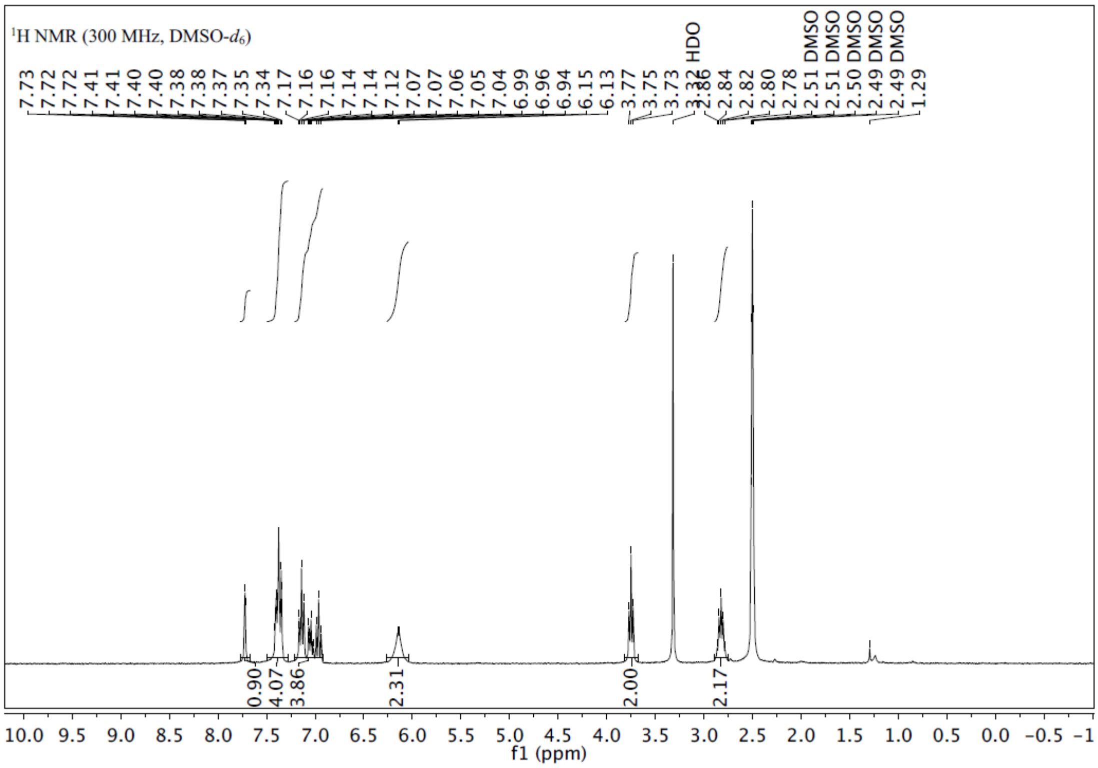
1H NMR (300 MHz, DMSO-d6)
7.73 7.72 7.72 7.41 7.41 7.40 7.40 7.38 7.38 7.37 7.35 7.34 7.17 7.16 7.16 7.14 7.14 7.12 7.07 7.07 7.06 7.05 7.04 6.99 6.96 6.94 6.15 6.13 3.77 3.75 3.73 3.32 HDO 2.86 2.84 2.82 2.80 2.78 2.51 DMSO 2.51 DMSO 2.50 DMSO 2.49 DMSO 2.49 DMSO 1.29
0.90 4.07 3.86 2.31 2.00 2.17
10.0 9.5 9.0 8.5 8.0 7.5 7.0 6.5 6.0 5.5 5.0 4.5 4.0 3.5 3.0 2.5 2.0 1.5 1.0 0.5 0.0 −0.5 −1
f1 (ppm)

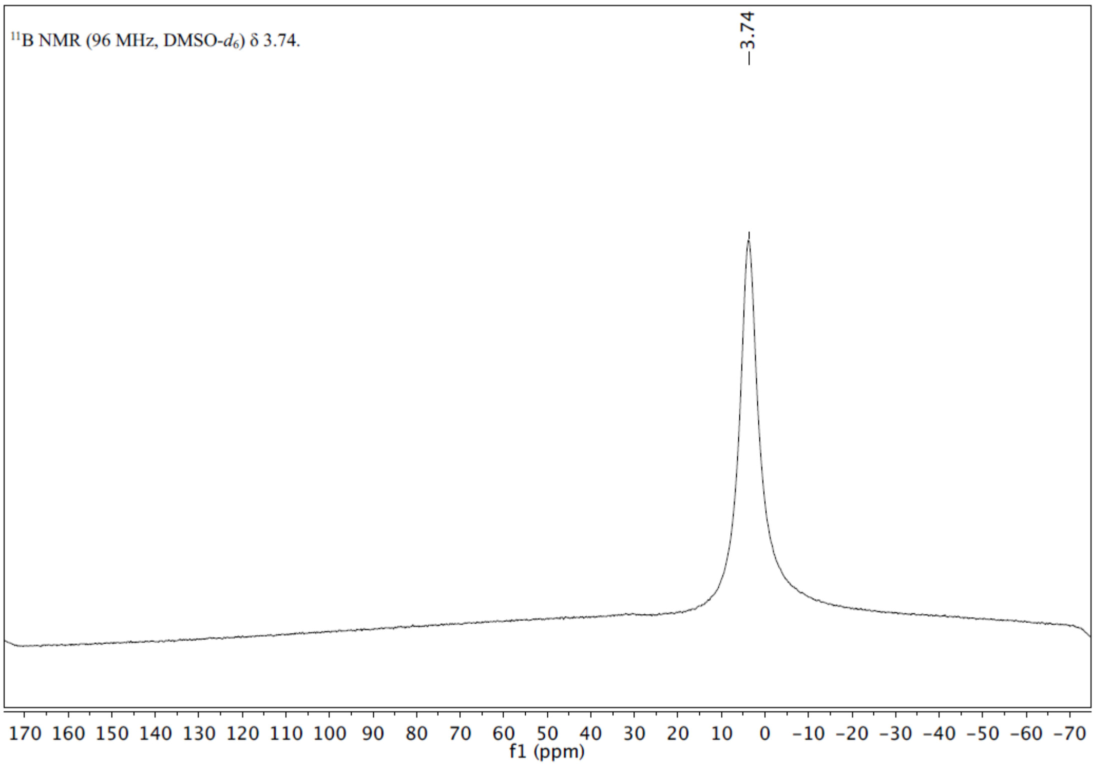
11B NMR (96 MHz, DMSO-d6) δ 3.74.
3.74
170 160 150 140 130 120 110 100 90 80 70 60 50 40 30 20 10 0 −10 −20 −30 −40 −50 −60 −70
f1 (ppm)

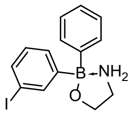

***m*-I-2APB**

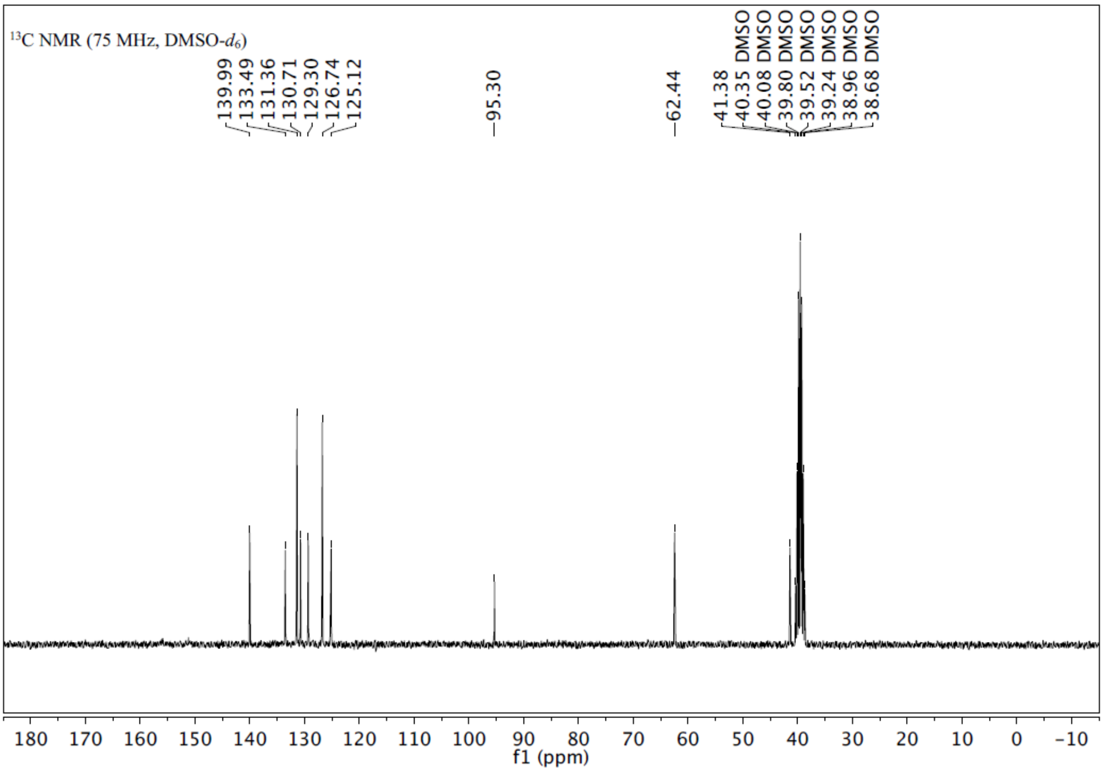

^{13}C NMR (75 MHz, DMSO-d_6)
139.99
133.49
131.36
130.71
129.30
126.74
125.12
95.30
62.44
41.38
40.35 DMSO
40.08 DMSO
39.80 DMSO
39.52 DMSO
39.24 DMSO
38.96 DMSO
38.68 DMSO
180 170 160 150 140 130 120 110 100 90 80 70 60 50 40 30 20 10 0 −10
f1 (ppm)

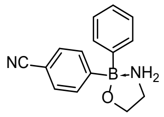
NC
B
NH2
O

***p*-CN-2APB**

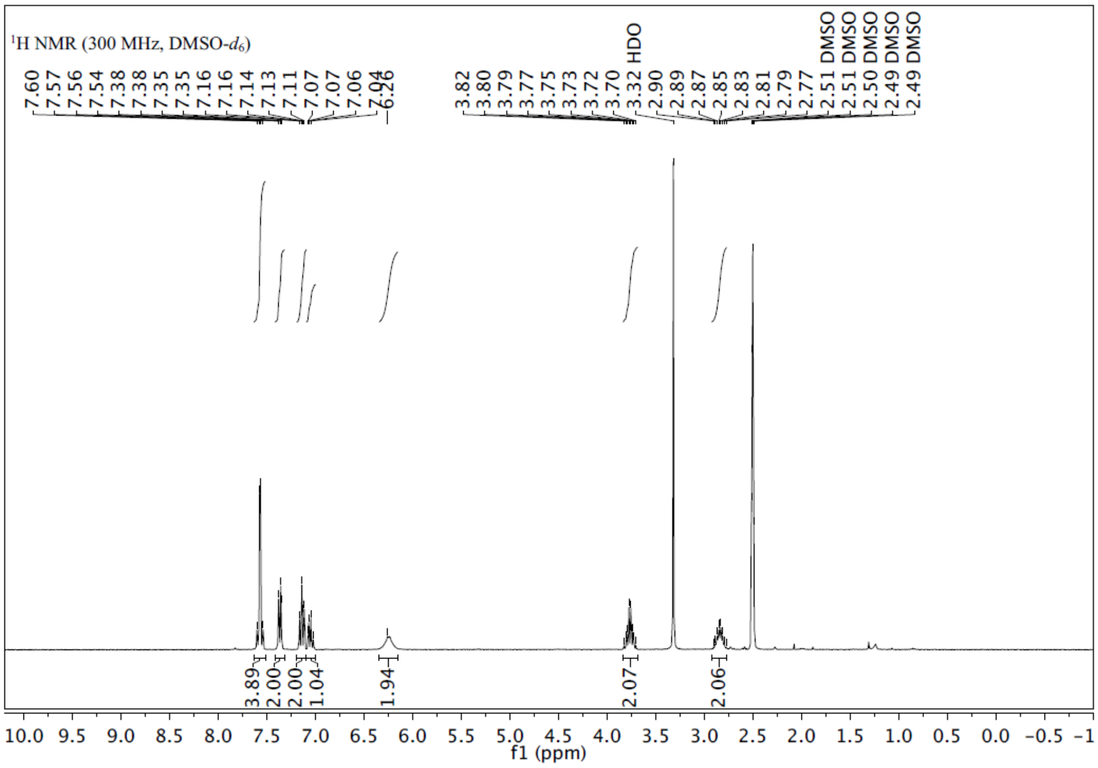
1H NMR (300 MHz, DMSO-d6)
7.60 7.57 7.56 7.54 7.38 7.38 7.35 7.35 7.16 7.16 7.14 7.13 7.11 7.07 7.07 7.06 7.04 6.26
3.82 3.80 3.79 3.77 3.75 3.73 3.72 3.70 3.32 HDO 2.90 2.89 2.87 2.85 2.83 2.81 2.79 2.77 2.51 DMSO 2.51 DMSO 2.50 DMSO 2.49 DMSO 2.49 DMSO
3.89 2.00 2.00 1.04 1.94 2.07 2.06
10.0 9.5 9.0 8.5 8.0 7.5 7.0 6.5 6.0 5.5 5.0 4.5 4.0 3.5 3.0 2.5 2.0 1.5 1.0 0.5 0.0 −0.5 −1
f1 (ppm)

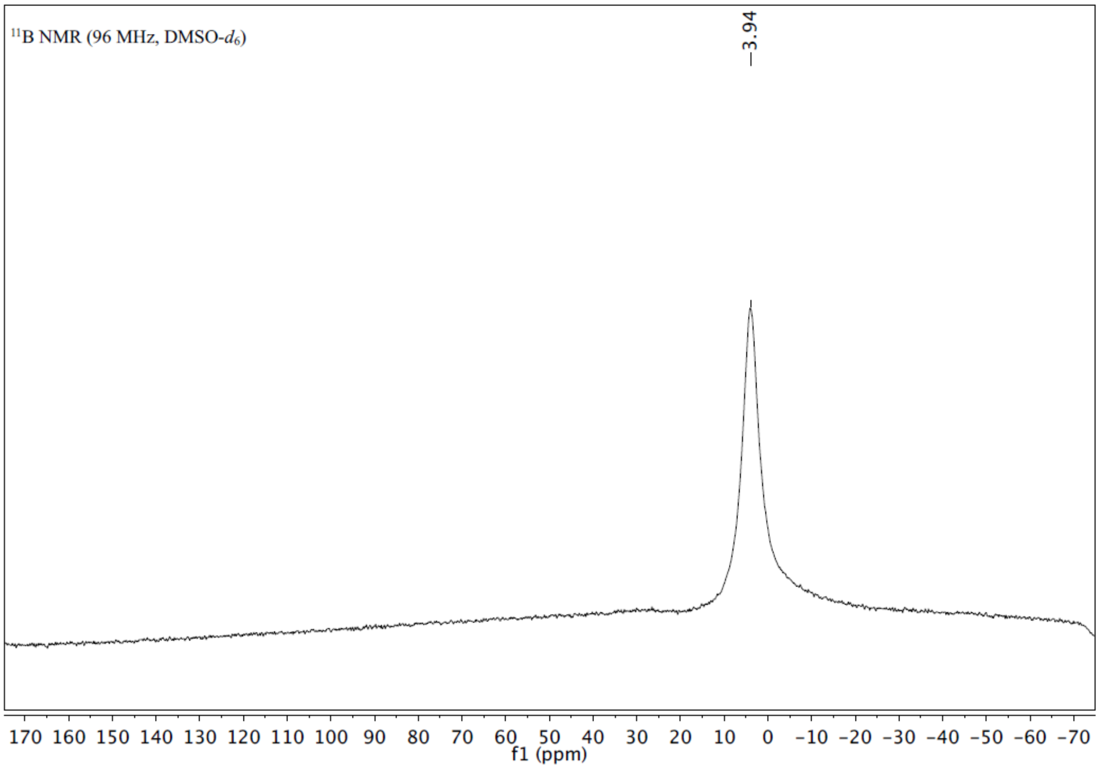
11B NMR (96 MHz, DMSO-d6)
3.94
170 160 150 140 130 120 110 100 90 80 70 60 50 40 30 20 10 0 −10 −20 −30 −40 −50 −60 −70
f1 (ppm)

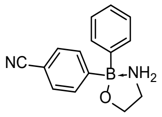

NC
B
NH2
O

***p*-CN-2APB**

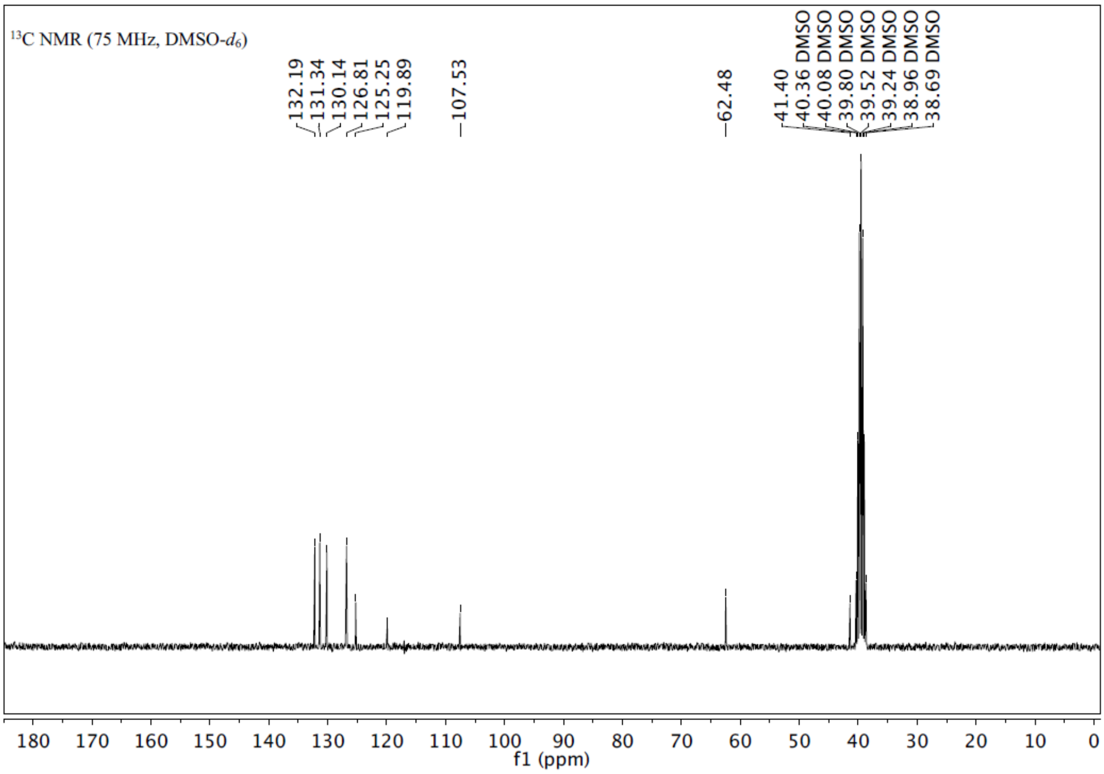

13C NMR (75 MHz, DMSO-d6)
132.19
131.34
130.14
126.81
125.25
119.89
107.53
62.48
41.40
40.36 DMSO
40.08 DMSO
39.80 DMSO
39.52 DMSO
39.24 DMSO
38.96 DMSO
38.69 DMSO
180 170 160 150 140 130 120 110 100 90 80 70 60 50 40 30 20 10 0
f1 (ppm)

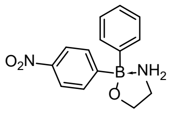
O2N
B
NH2
O

p-NO$_2$-2APB

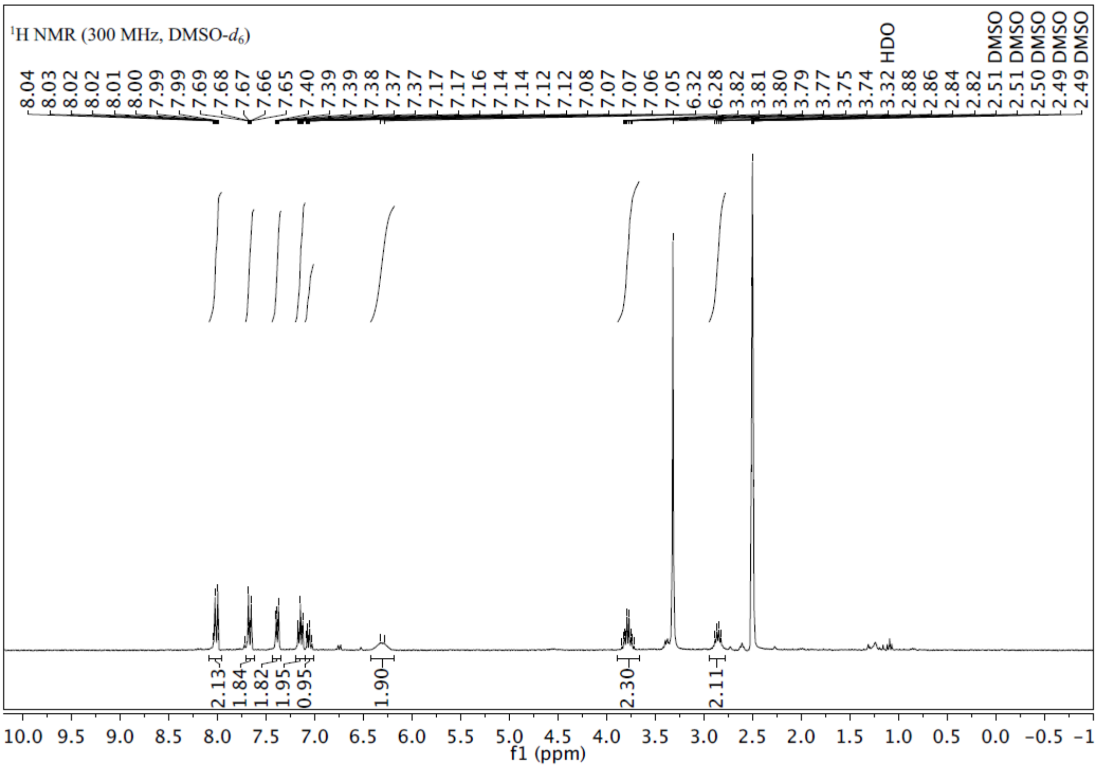
1H NMR (300 MHz, DMSO-d6)
8.04 8.03 8.02 8.02 8.01 8.00 7.99 7.99 7.69 7.68 7.67 7.66 7.65 7.40 7.39 7.38 7.37 7.37 7.17 7.17 7.16 7.14 7.14 7.12 7.12 7.08 7.07 7.06 7.05 6.32 6.28 3.82 3.81 3.80 3.79 3.77 3.75 3.74
3.32 HDO
2.88 2.86 2.84 2.82
2.51 DMSO
2.51 DMSO
2.50 DMSO
2.49 DMSO
2.49 DMSO
2.13 1.84 1.82 1.95 0.95 1.90 2.30 2.11
10.0 9.5 9.0 8.5 8.0 7.5 7.0 6.5 6.0 5.5 5.0 4.5 4.0 3.5 3.0 2.5 2.0 1.5 1.0 0.5 0.0 −0.5 −1
f1 (ppm)

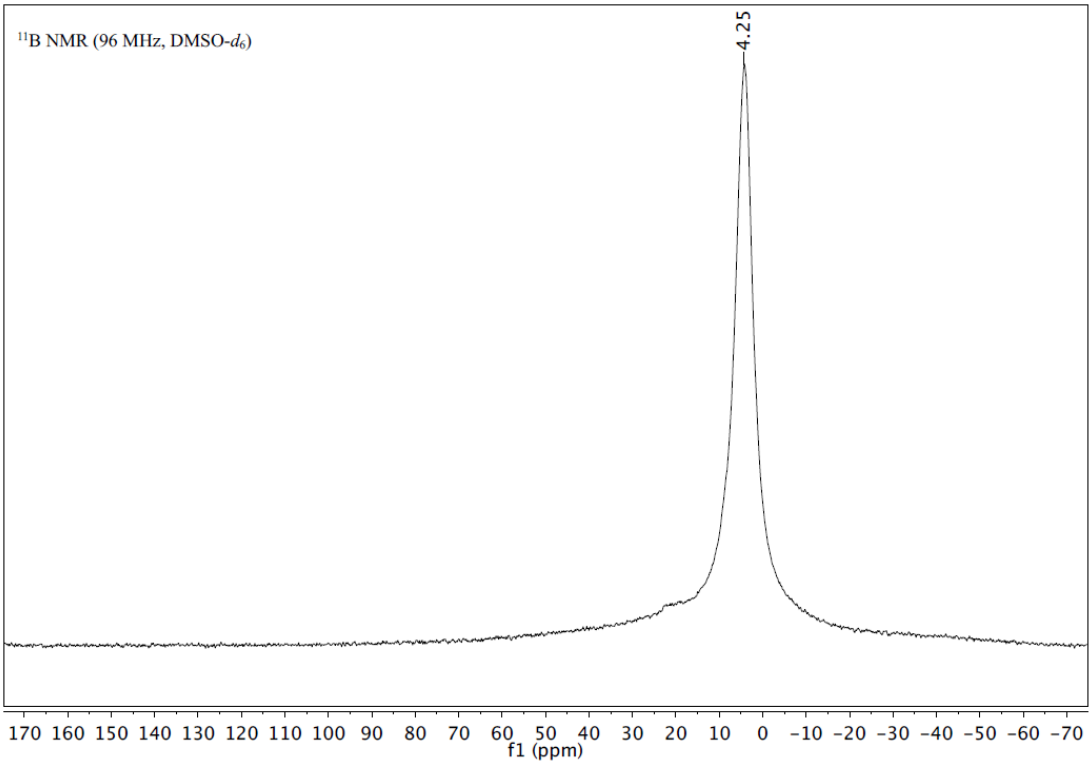
11B NMR (96 MHz, DMSO-d6)
4.25
170 160 150 140 130 120 110 100 90 80 70 60 50 40 30 20 10 0 −10 −20 −30 −40 −50 −60 −70
f1 (ppm)

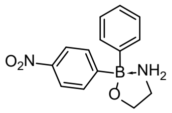

***p*-NO₂-2APB**

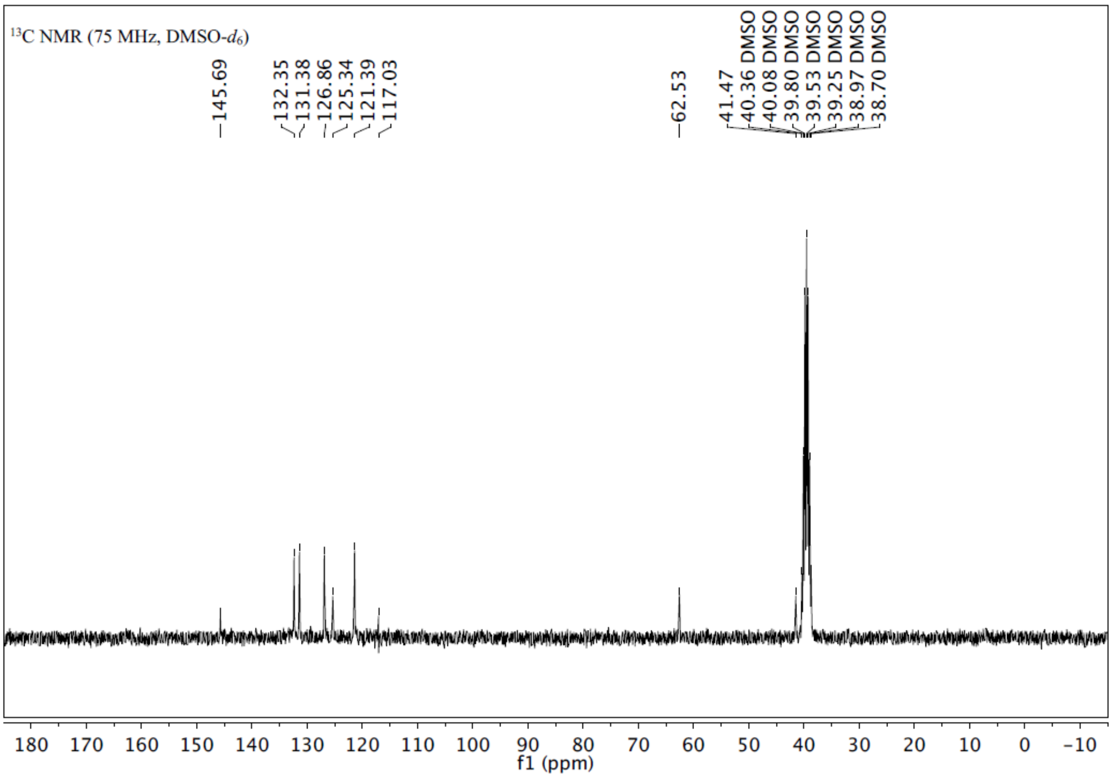

¹³C NMR (75 MHz, DMSO-d6)
145.69
132.35
131.38
126.86
125.34
121.39
117.03
62.53
41.47
40.36 DMSO
40.08 DMSO
39.80 DMSO
39.53 DMSO
39.25 DMSO
38.97 DMSO
38.70 DMSO
180 170 160 150 140 130 120 110 100 90 80 70 60 50 40 30 20 10 0 −10
f1 (ppm)

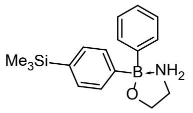
Me3Si
B
NH2
O

***p*-TMS-2APB**

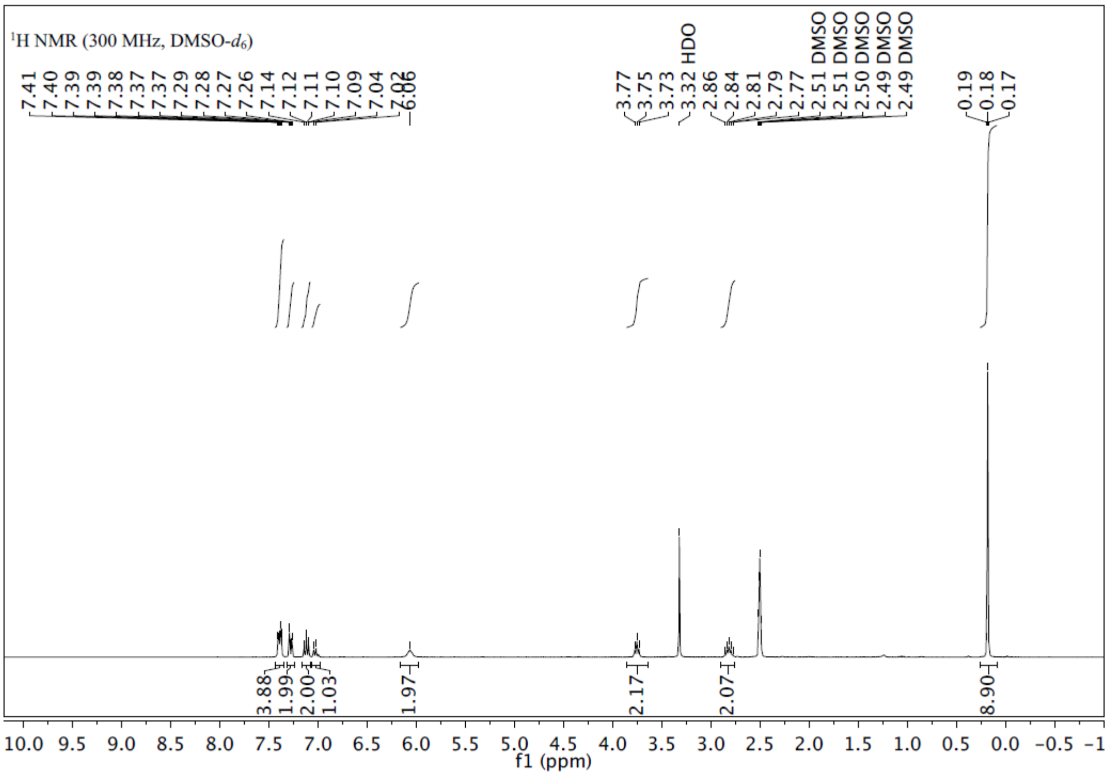
1H NMR (300 MHz, DMSO-d6)
7.41 7.40 7.39 7.39 7.38 7.37 7.37 7.29 7.28 7.27 7.26 7.14 7.12 7.11 7.10 7.09 7.04 6.06
3.77 3.75 3.73 3.32 HDO 2.86 2.84 2.81 2.79 2.77 2.51 DMSO 2.51 DMSO 2.50 DMSO 2.49 DMSO 2.49 DMSO
0.19 0.18 0.17
3.88 1.99 2.00 1.03 1.97 2.17 2.07 8.90
10.0 9.5 9.0 8.5 8.0 7.5 7.0 6.5 6.0 5.5 5.0 4.5 4.0 3.5 3.0 2.5 2.0 1.5 1.0 0.5 0.0 −0.5 −1
f1 (ppm)

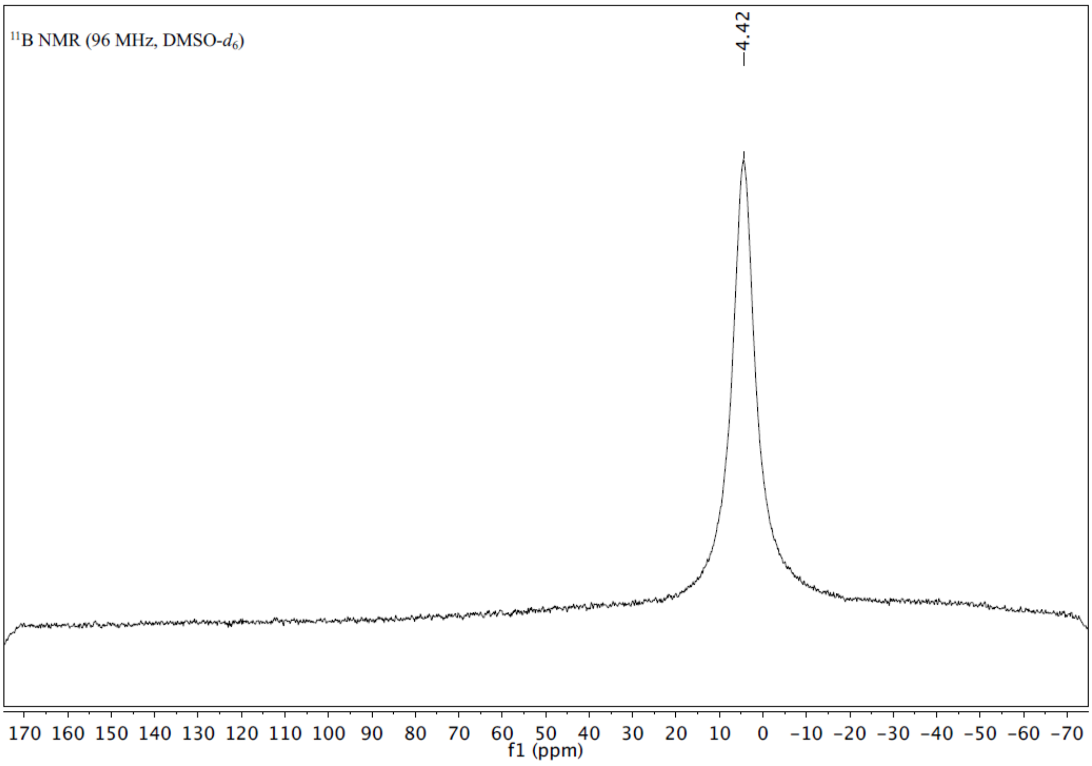
11B NMR (96 MHz, DMSO-d6)
4.42
170 160 150 140 130 120 110 100 90 80 70 60 50 40 30 20 10 0 −10 −20 −30 −40 −50 −60 −70
f1 (ppm)

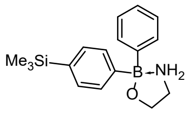

p-TMS-2APB

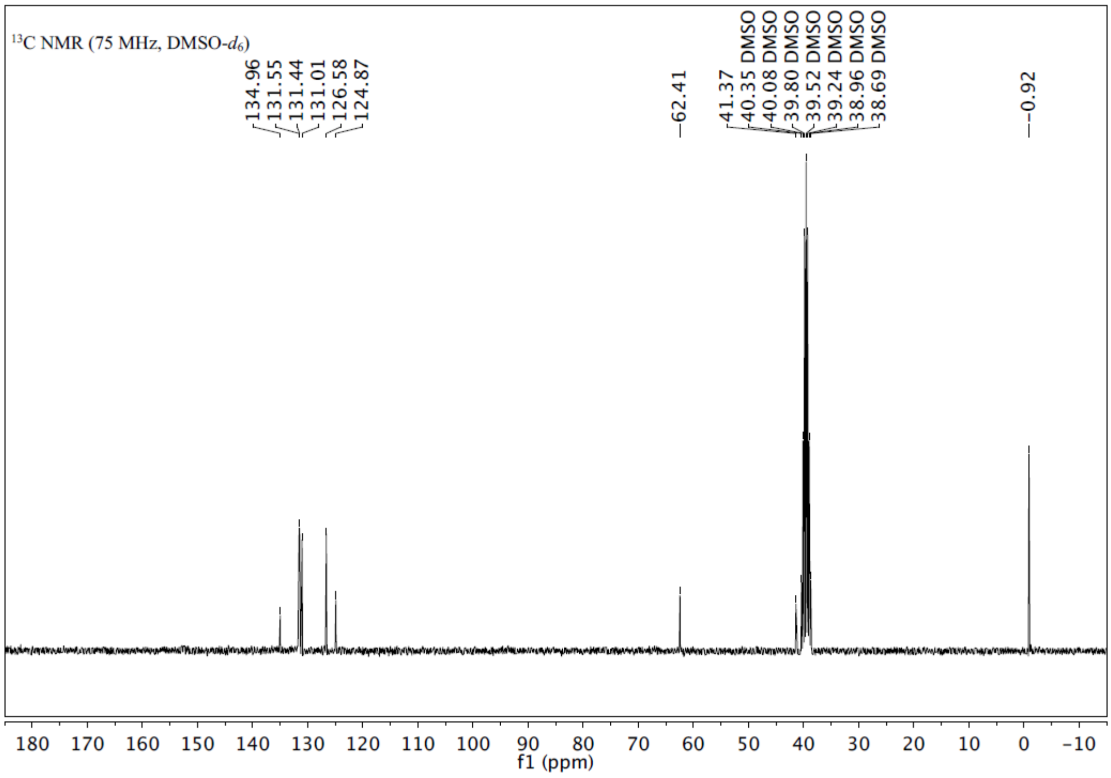

^{13}C NMR (75 MHz, DMSO-d_6)
134.96
131.55
131.44
131.01
126.58
124.87
62.41
41.37
40.35 DMSO
40.08 DMSO
39.80 DMSO
39.52 DMSO
39.24 DMSO
38.96 DMSO
38.69 DMSO
0.92
180 170 160 150 140 130 120 110 100 90 80 70 60 50 40 30 20 10 0 -10
f1 (ppm)

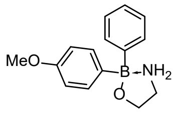
MeO
B
NH2
O

***p*-OMe-2APB**

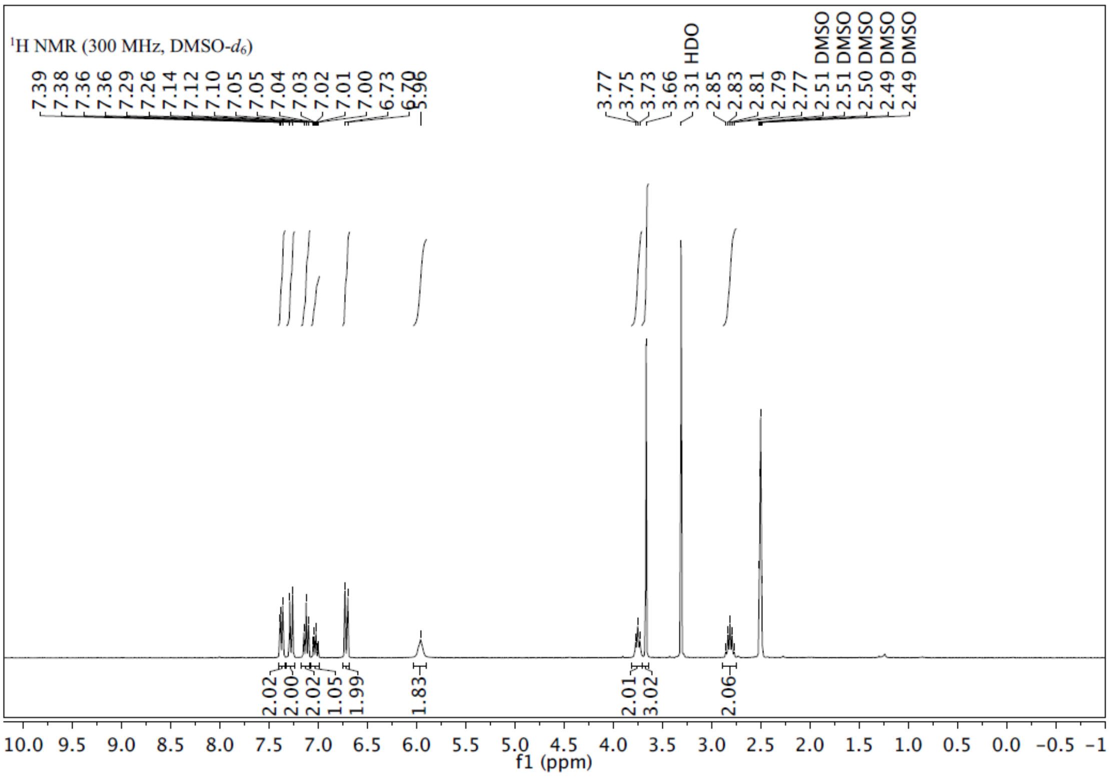
1H NMR (300 MHz, DMSO-d6)
7.39
7.38
7.36
7.36
7.29
7.26
7.14
7.12
7.10
7.05
7.05
7.04
7.03
7.02
7.01
7.00
6.73
6.70
5.96
3.77
3.75
3.73
3.66
3.31 HDO
2.85
2.83
2.81
2.79
2.77
2.51 DMSO
2.51 DMSO
2.50 DMSO
2.49 DMSO
2.49 DMSO
2.02
2.00
2.02
1.05
1.99
1.83
2.01
3.02
2.06
10.0 9.5 9.0 8.5 8.0 7.5 7.0 6.5 6.0 5.5 5.0 4.5 4.0 3.5 3.0 2.5 2.0 1.5 1.0 0.5 0.0 −0.5 −1
f1 (ppm)

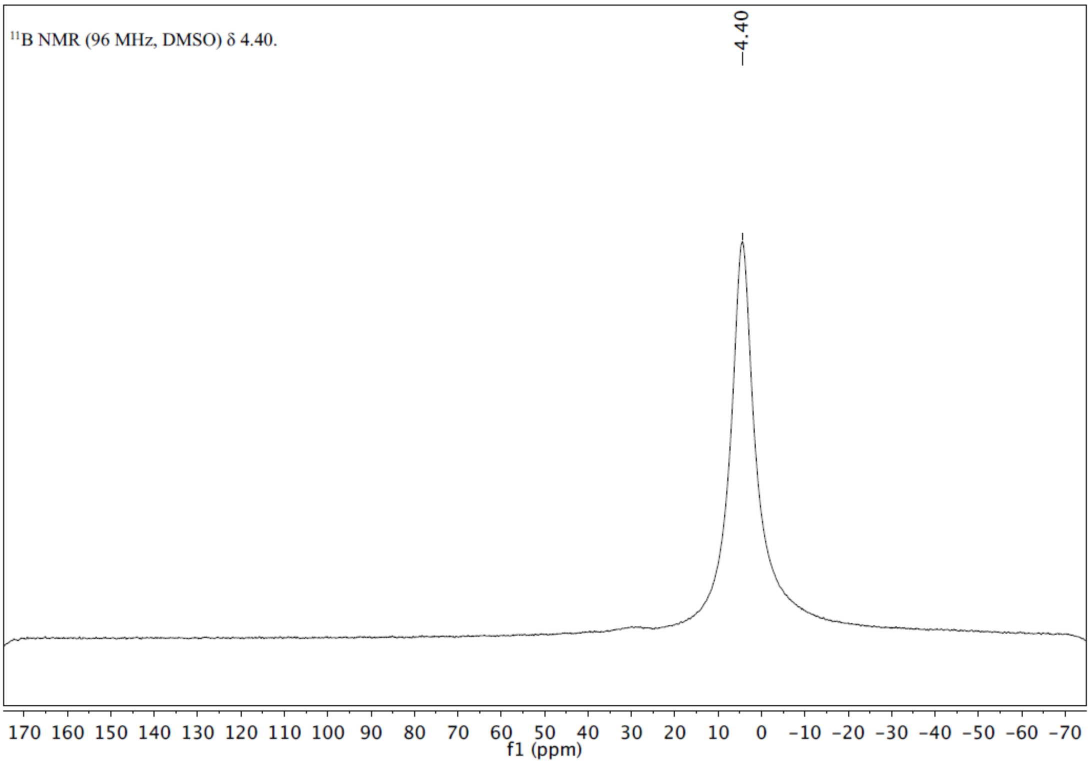
11B NMR (96 MHz, DMSO) δ 4.40.
4.40
170 160 150 140 130 120 110 100 90 80 70 60 50 40 30 20 10 0 −10 −20 −30 −40 −50 −60 −70
f1 (ppm)

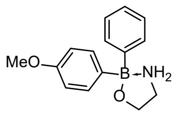
MeO
B
NH2
O

***p*-OMe-2APB**

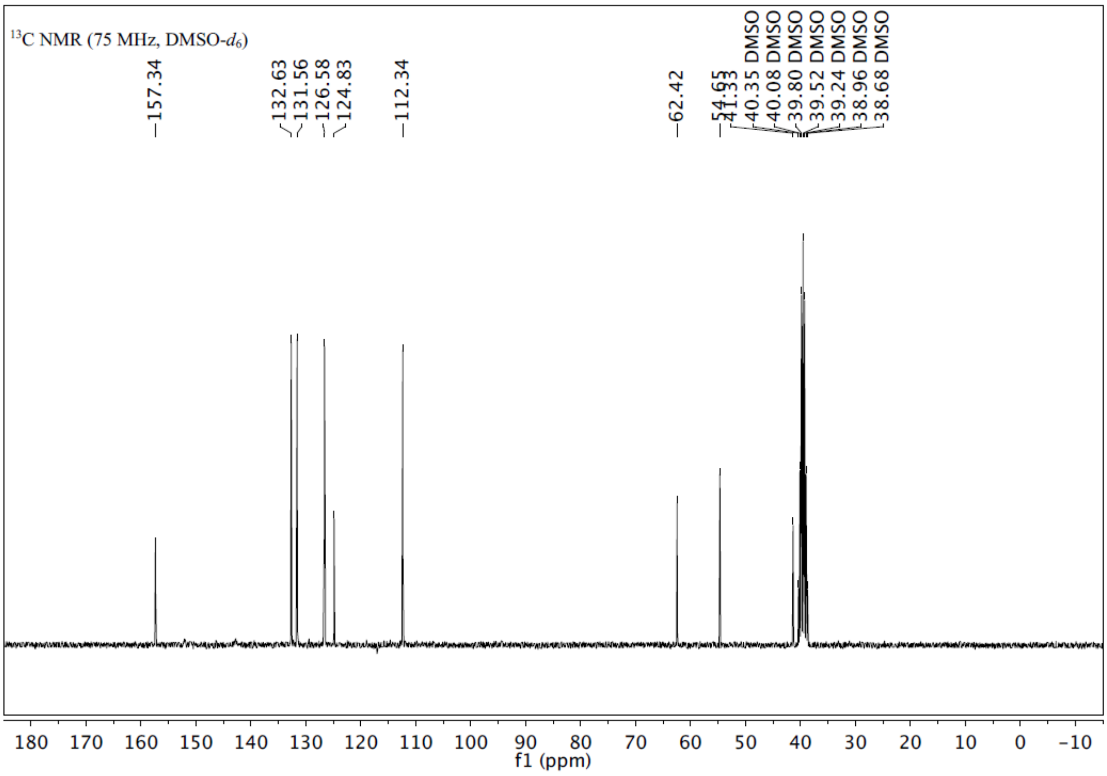
13C NMR (75 MHz, DMSO-d6)
157.34
132.63
131.56
126.58
124.83
112.34
62.42
54.65
41.33
40.35 DMSO
40.08 DMSO
39.80 DMSO
39.52 DMSO
39.24 DMSO
38.96 DMSO
38.68 DMSO
180 170 160 150 140 130 120 110 100 90 80 70 60 50 40 30 20 10 0 −10
f1 (ppm)

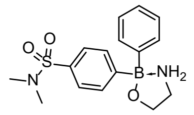

p-SO2NMe2-2APB

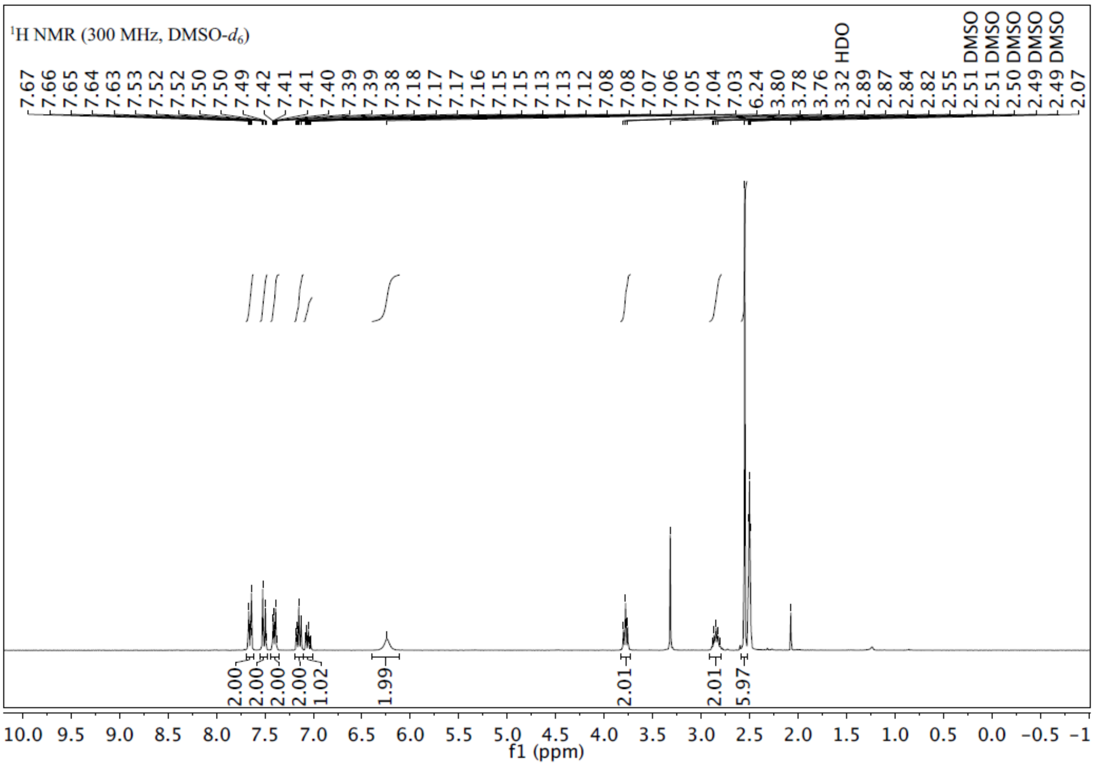

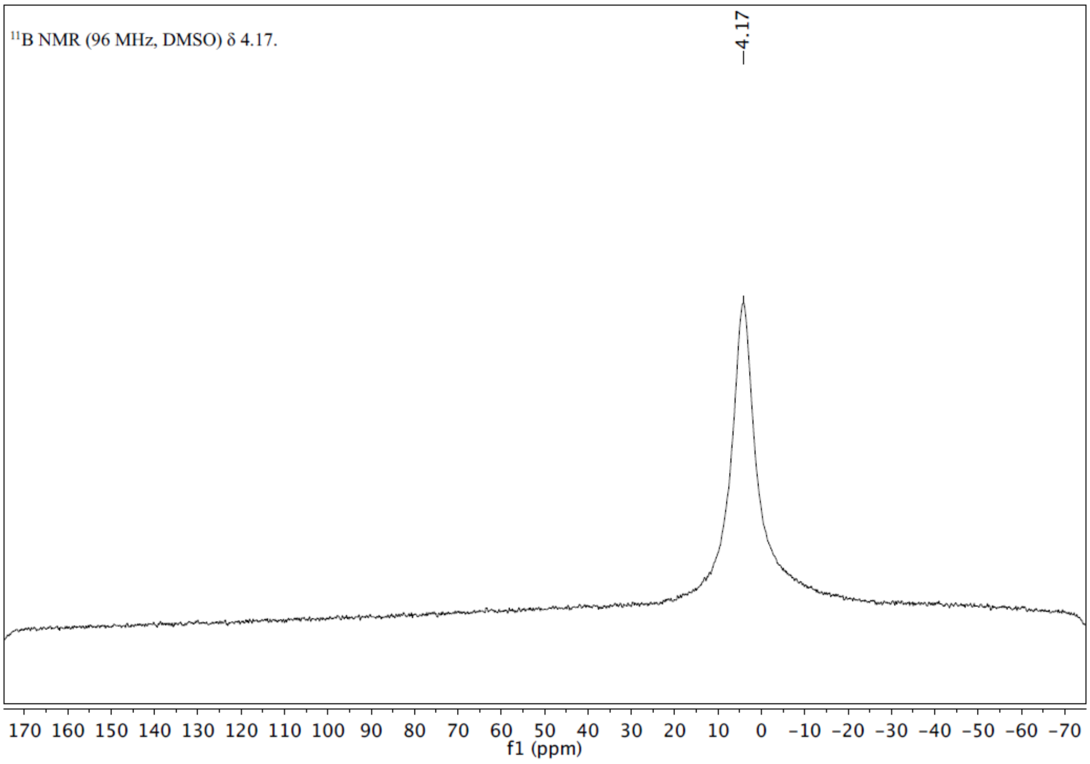

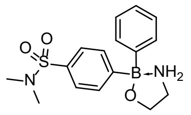

***p*-SO₂NMe₂-2APB**

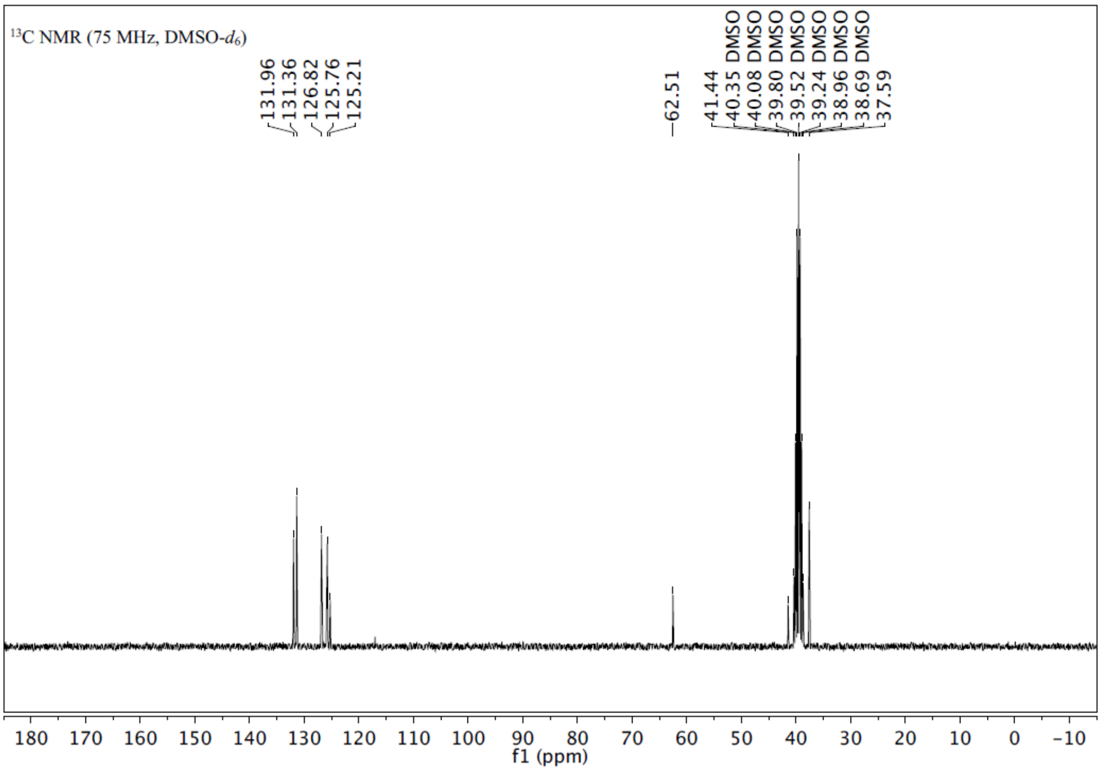

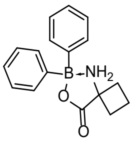

B
NH2
O
O

cBu-2APB

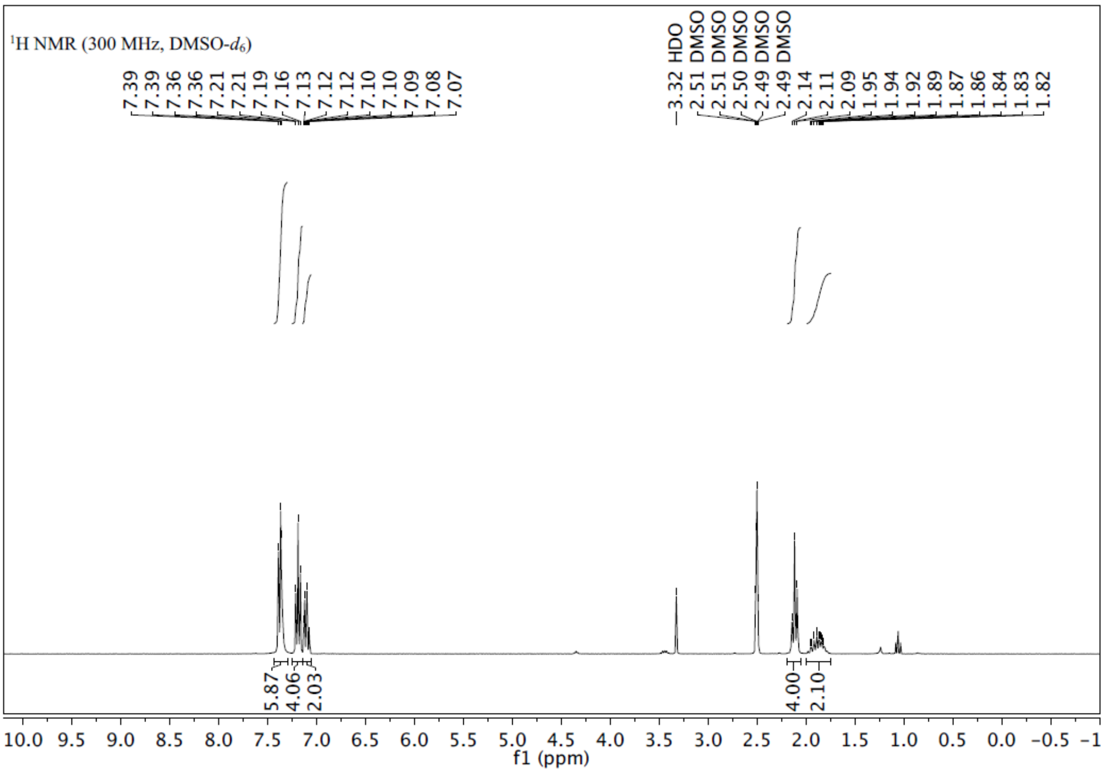

1H NMR (300 MHz, DMSO-d6)
7.39
7.39
7.36
7.36
7.21
7.21
7.19
7.16
7.13
7.12
7.12
7.10
7.10
7.09
7.08
7.07
3.32 HDO
2.51 DMSO
2.50 DMSO
2.49 DMSO
2.49 DMSO
2.49 DMSO
2.14
2.11
2.09
1.95
1.94
1.92
1.89
1.87
1.86
1.84
1.83
1.82
5.87
4.06
2.03
4.00
2.10
10.0 9.5 9.0 8.5 8.0 7.5 7.0 6.5 6.0 5.5 5.0 4.5 4.0 3.5 3.0 2.5 2.0 1.5 1.0 0.5 0.0 −0.5 −1
f1 (ppm)

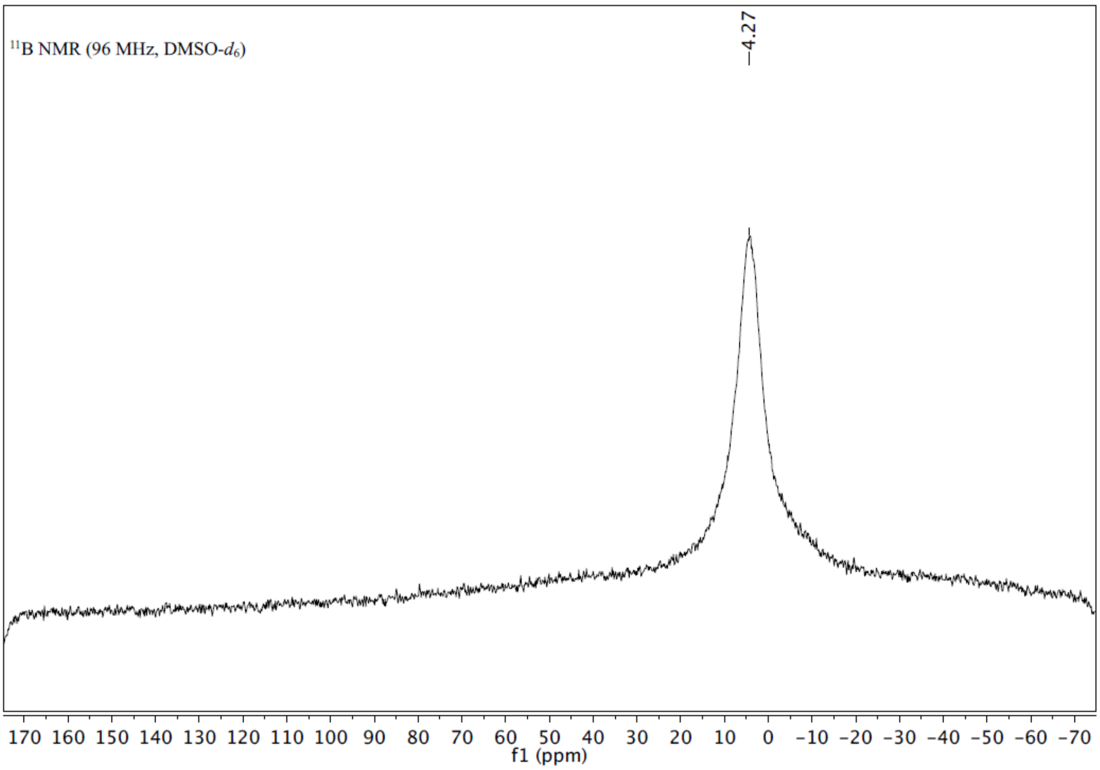

11B NMR (96 MHz, DMSO-d6)
4.27
170 160 150 140 130 120 110 100 90 80 70 60 50 40 30 20 10 0 −10 −20 −30 −40 −50 −60 −70
f1 (ppm)

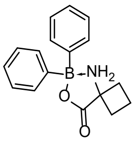

cBu-2APB

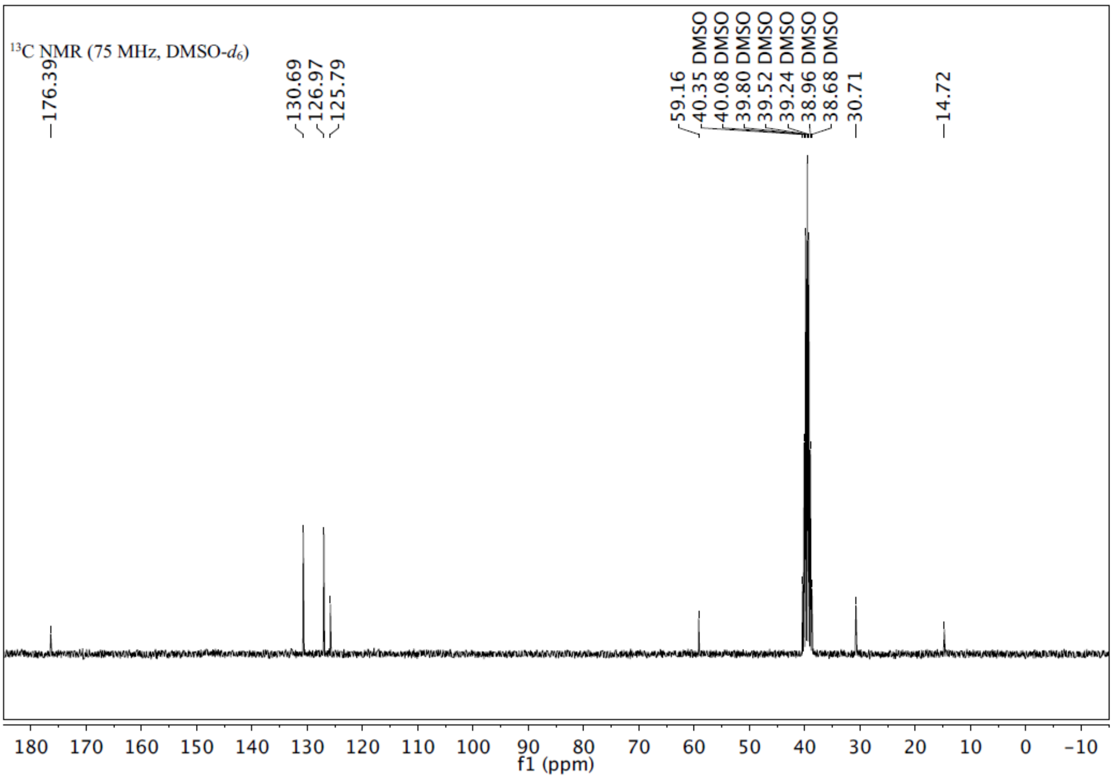

^{13}C NMR (75 MHz, DMSO-d_6)
176.39
130.69
126.97
125.79
59.16
40.35 DMSO
40.08 DMSO
39.80 DMSO
39.52 DMSO
39.24 DMSO
38.96 DMSO
38.68 DMSO
30.71
14.72
180 170 160 150 140 130 120 110 100 90 80 70 60 50 40 30 20 10 0 -10
f1 (ppm)

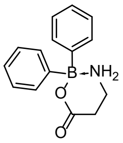
B
NH2
O
O

β-Ala-2APB

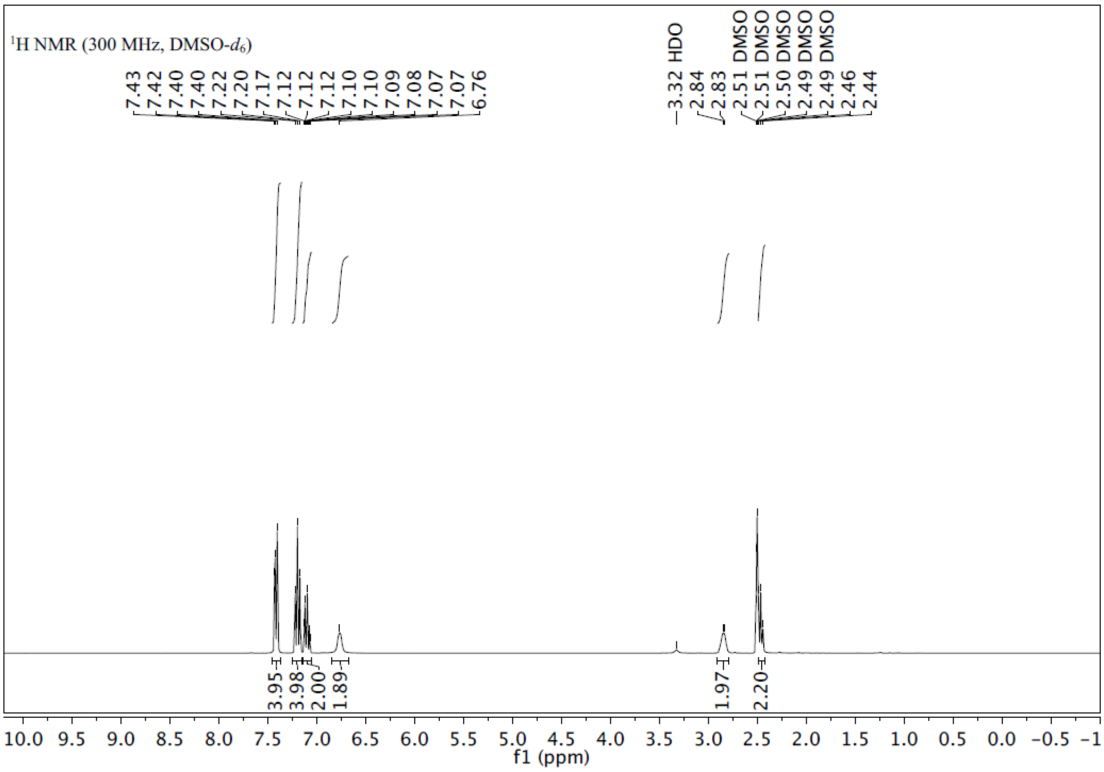
1H NMR (300 MHz, DMSO-d6)
7.43
7.42
7.40
7.40
7.22
7.20
7.17
7.12
7.12
7.12
7.10
7.10
7.09
7.08
7.07
7.07
6.76
3.32 HDO
2.84
2.83
2.51 DMSO
2.51 DMSO
2.50 DMSO
2.49 DMSO
2.49 DMSO
2.46
2.44
3.95
3.98
2.00
1.89
1.97
2.20
10.0 9.5 9.0 8.5 8.0 7.5 7.0 6.5 6.0 5.5 5.0 4.5 4.0 3.5 3.0 2.5 2.0 1.5 1.0 0.5 0.0 −0.5 −1
f1 (ppm)

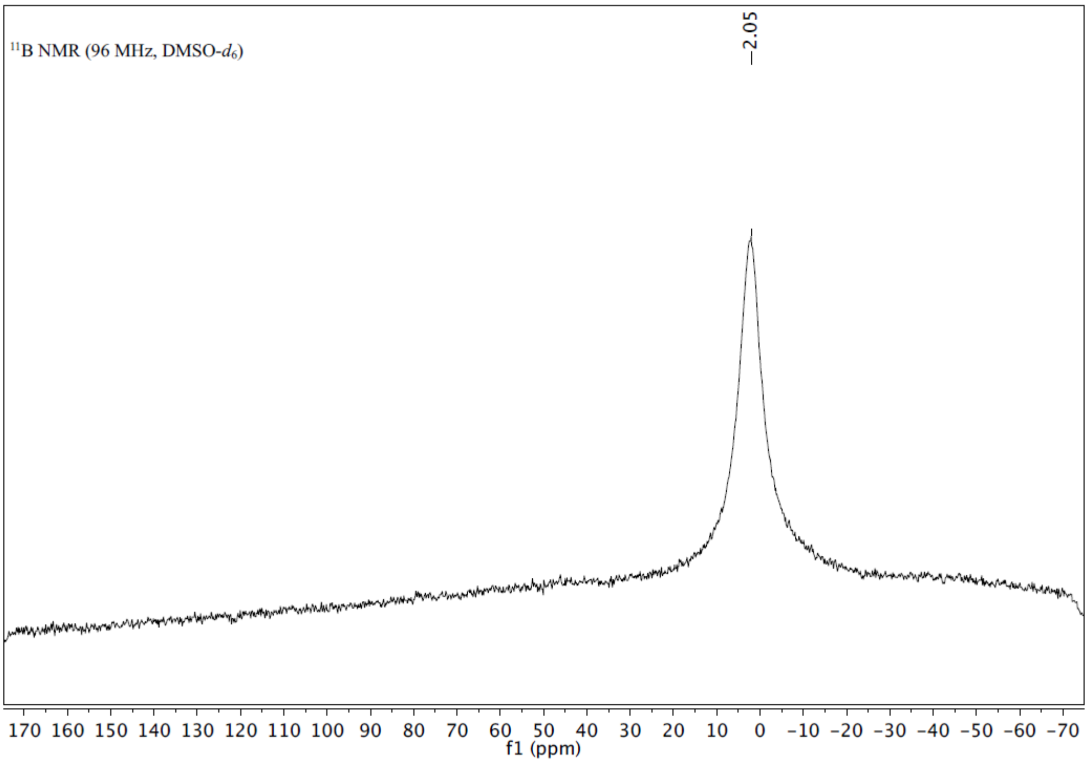
11B NMR (96 MHz, DMSO-d6)
2.05
170 160 150 140 130 120 110 100 90 80 70 60 50 40 30 20 10 0 −10 −20 −30 −40 −50 −60 −70
f1 (ppm)

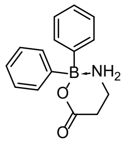

B
NH_2
O
O

β-Ala-2APB

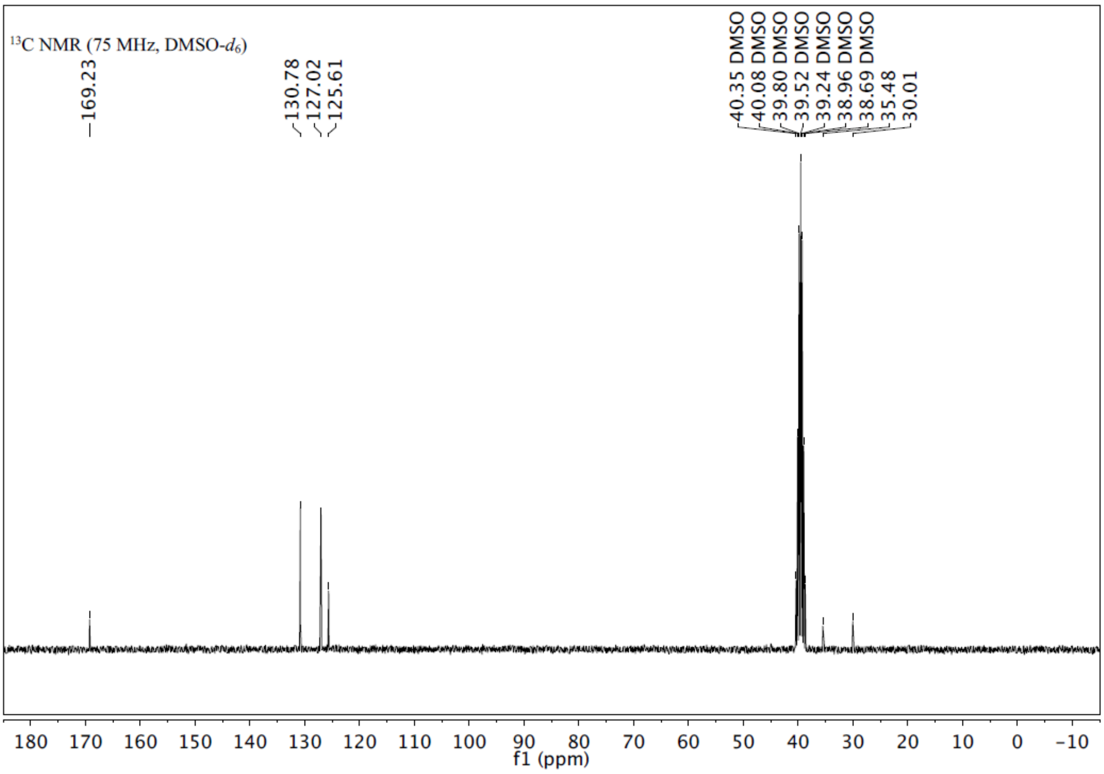

^{13}C NMR (75 MHz, DMSO-d_6)
169.23
130.78
127.02
125.61
40.35 DMSO
40.08 DMSO
39.80 DMSO
39.52 DMSO
39.24 DMSO
38.96 DMSO
38.69 DMSO
35.48
30.01
180 170 160 150 140 130 120 110 100 90 80 70 60 50 40 30 20 10 0 −10
f1 (ppm)

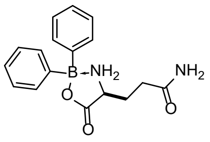

Gln-2APB

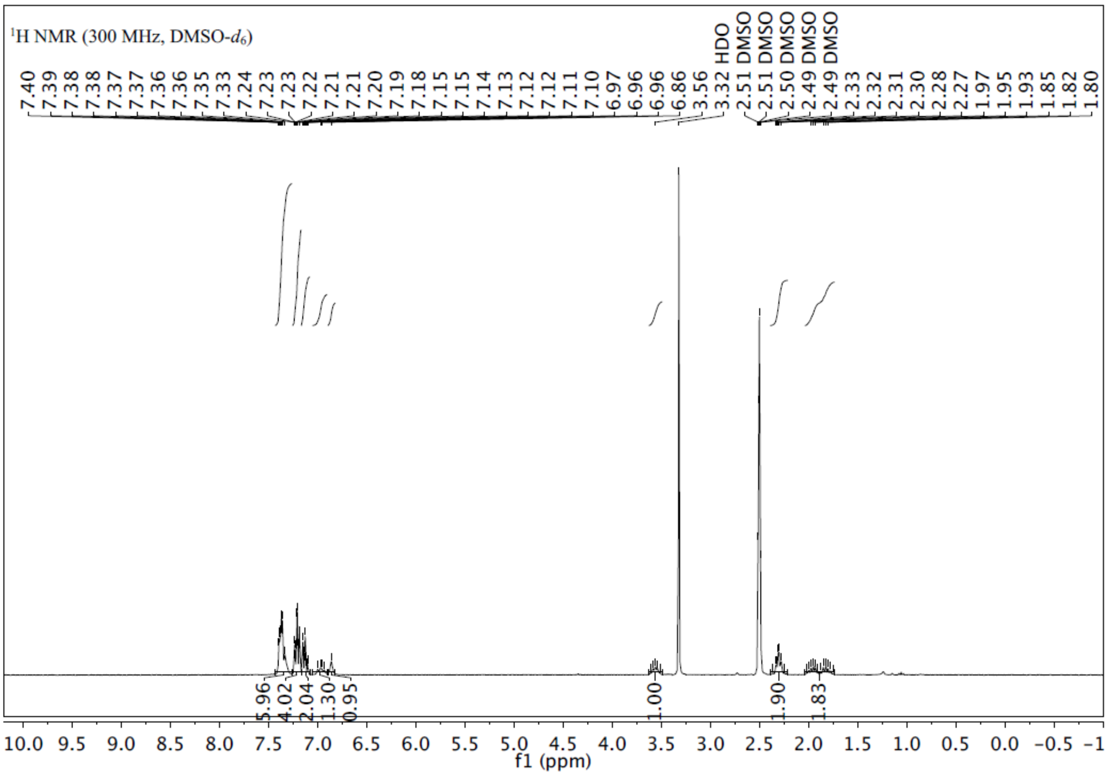

1H NMR (300 MHz, DMSO-d6)
7.40 7.39 7.38 7.38 7.37 7.37 7.36 7.36 7.35 7.33 7.24 7.23 7.23 7.22 7.21 7.21 7.20 7.19 7.18 7.15 7.15 7.14 7.13 7.12 7.12 7.11 7.10 6.97 6.96 6.86 3.56 3.32 HDO 2.51 DMSO 2.51 DMSO 2.50 DMSO 2.49 DMSO 2.49 DMSO 2.33 2.32 2.31 2.30 2.28 2.27 1.97 1.95 1.93 1.85 1.82 1.80
5.96 4.02 2.04 1.30 0.95 1.00 1.90 1.83
10.0 9.5 9.0 8.5 8.0 7.5 7.0 6.5 6.0 5.5 5.0 4.5 4.0 3.5 3.0 2.5 2.0 1.5 1.0 0.5 0.0 −0.5 −1
f1 (ppm)

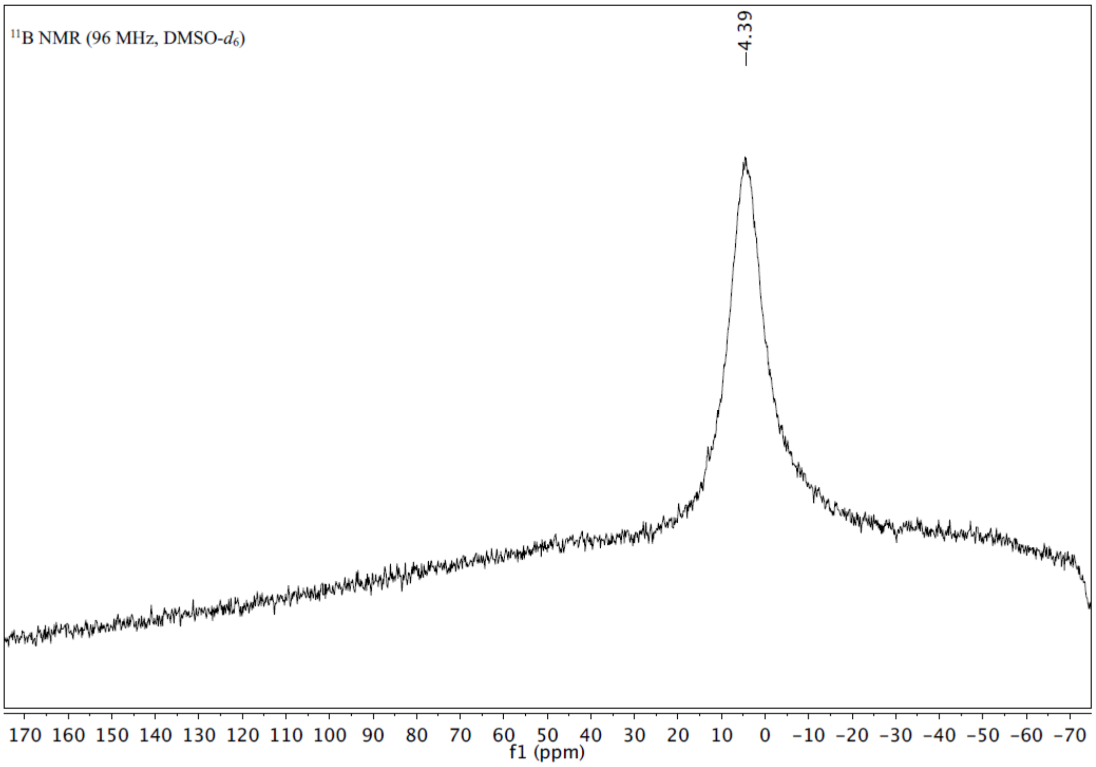

11B NMR (96 MHz, DMSO-d6)
4.39
170 160 150 140 130 120 110 100 90 80 70 60 50 40 30 20 10 0 −10 −20 −30 −40 −50 −60 −70
f1 (ppm)

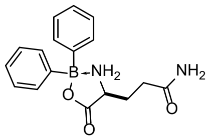

Gln-2APB

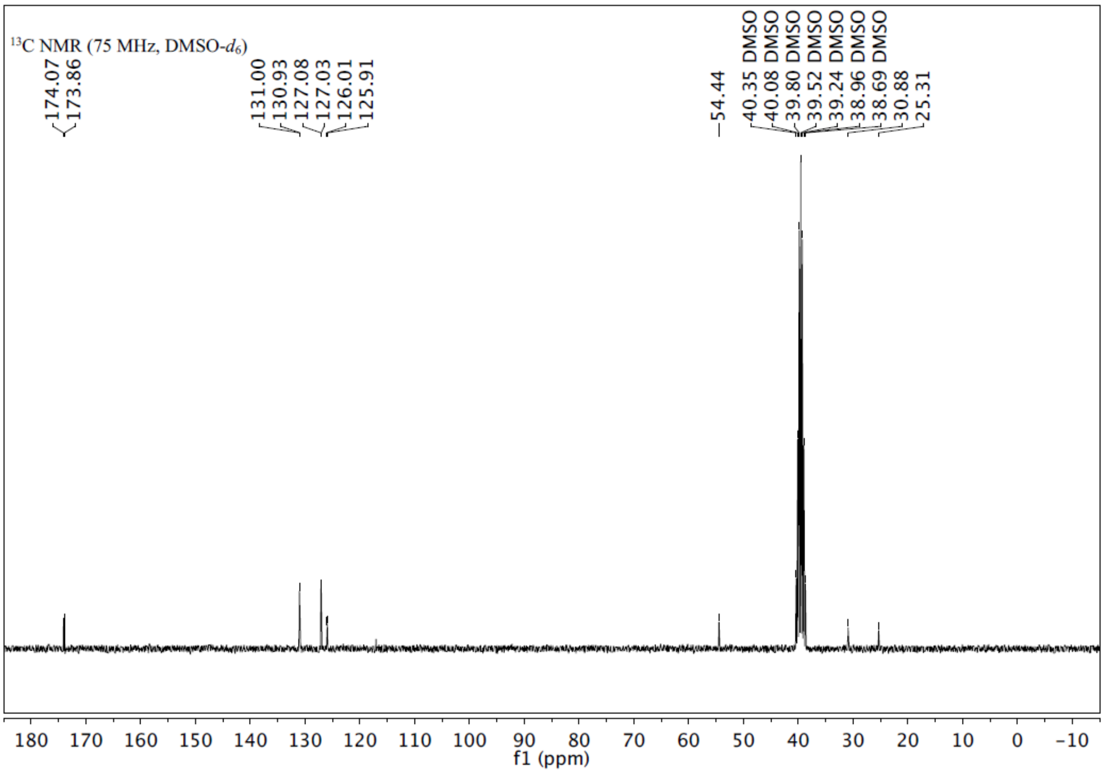

^{13}C NMR (75 MHz, DMSO-d_6)
174.07
173.86
131.00
130.93
127.08
127.03
126.01
125.91
54.44
40.35 DMSO
40.08 DMSO
39.80 DMSO
39.52 DMSO
39.24 DMSO
38.96 DMSO
38.69 DMSO
30.88
25.31
180 170 160 150 140 130 120 110 100 90 80 70 60 50 40 30 20 10 0 −10
f1 (ppm)

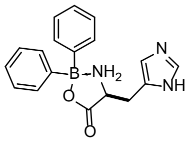

B
NH2
O
O
N
NH

His-2APB

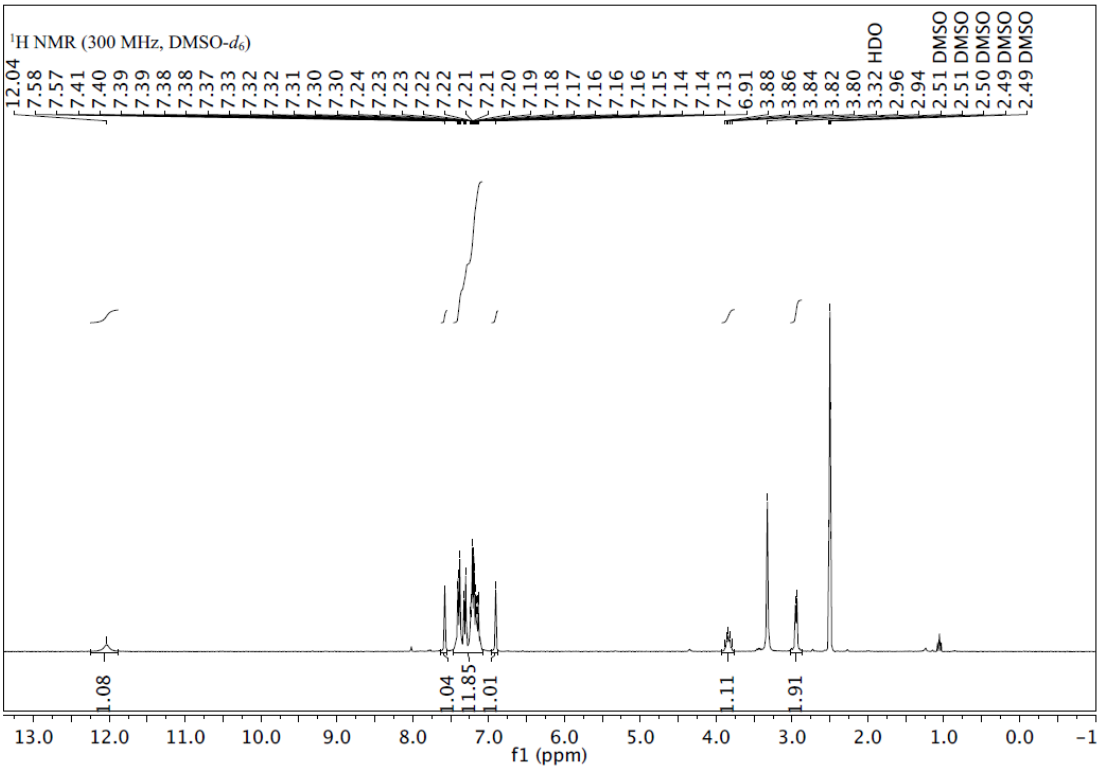

1H NMR (300 MHz, DMSO-d6)
12.04
7.58
7.57
7.41
7.40
7.39
7.39
7.38
7.38
7.37
7.33
7.32
7.32
7.31
7.30
7.30
7.24
7.23
7.23
7.22
7.22
7.21
7.21
7.20
7.19
7.18
7.17
7.16
7.16
7.15
7.14
7.14
7.13
6.91
3.88
3.86
3.84
3.82
3.80
3.32 HDO
2.96
2.94
2.51 DMSO
2.51 DMSO
2.50 DMSO
2.49 DMSO
2.49 DMSO
1.08
1.04
11.85
1.01
1.11
1.91
13.0 12.0 11.0 10.0 9.0 8.0 7.0 6.0 5.0 4.0 3.0 2.0 1.0 0.0 -1
f1 (ppm)

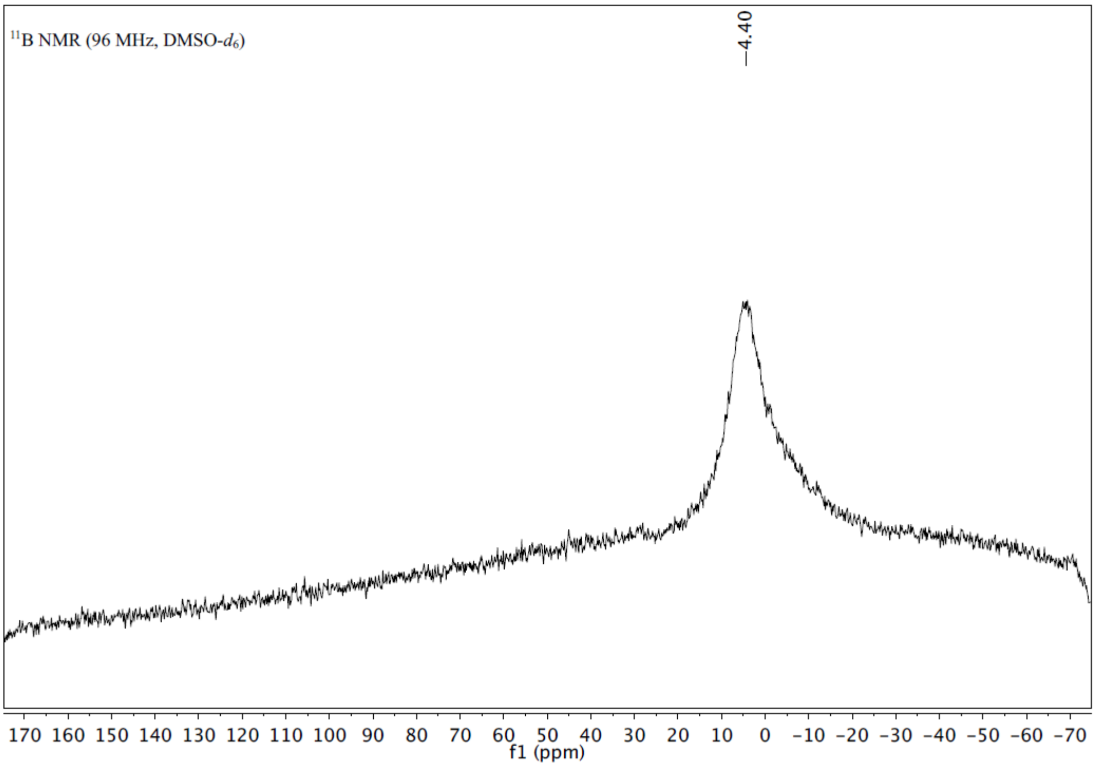

11B NMR (96 MHz, DMSO-d6)
4.40
170 160 150 140 130 120 110 100 90 80 70 60 50 40 30 20 10 0 -10 -20 -30 -40 -50 -60 -70
f1 (ppm)

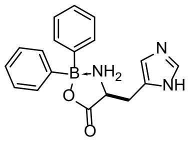
B
NH2
O
O
N
NH

His-2APB

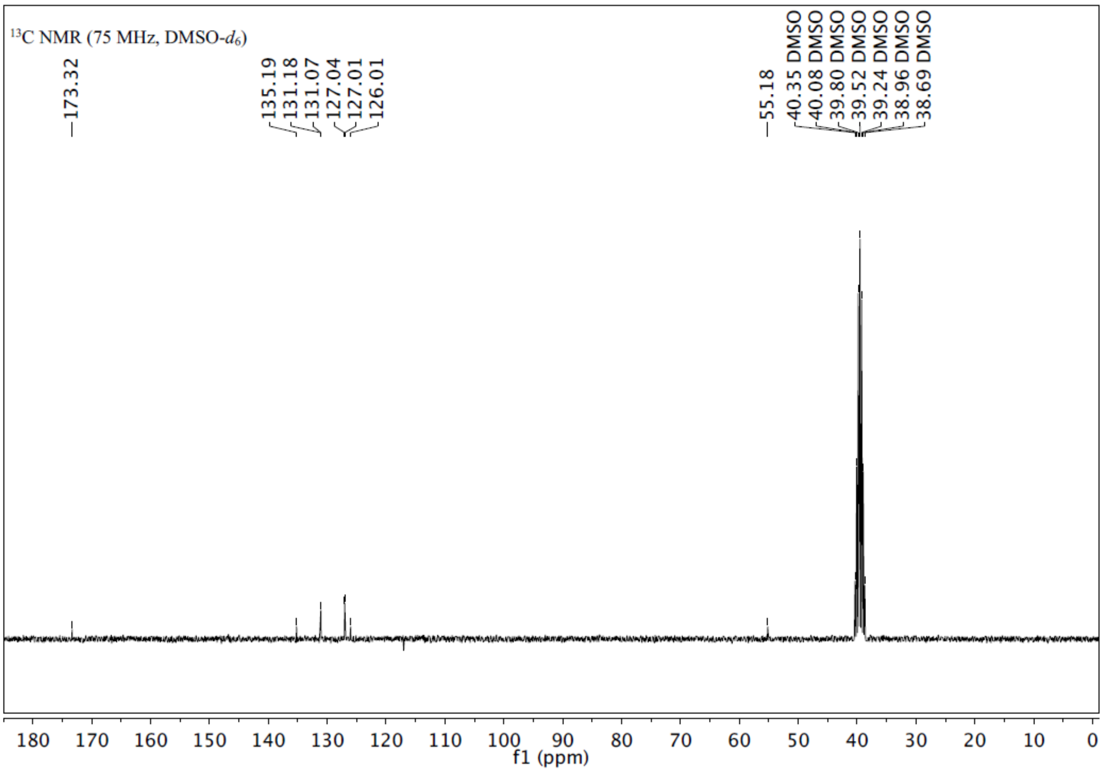
13C NMR (75 MHz, DMSO-d6)
173.32
135.19
131.18
131.07
127.04
127.01
126.01
55.18
40.35 DMSO
40.08 DMSO
39.80 DMSO
39.52 DMSO
39.24 DMSO
38.96 DMSO
38.69 DMSO
180 170 160 150 140 130 120 110 100 90 80 70 60 50 40 30 20 10 0
f1 (ppm)

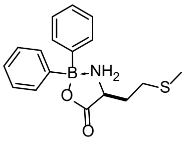
NH2
O
B
O
S

Met-2APB

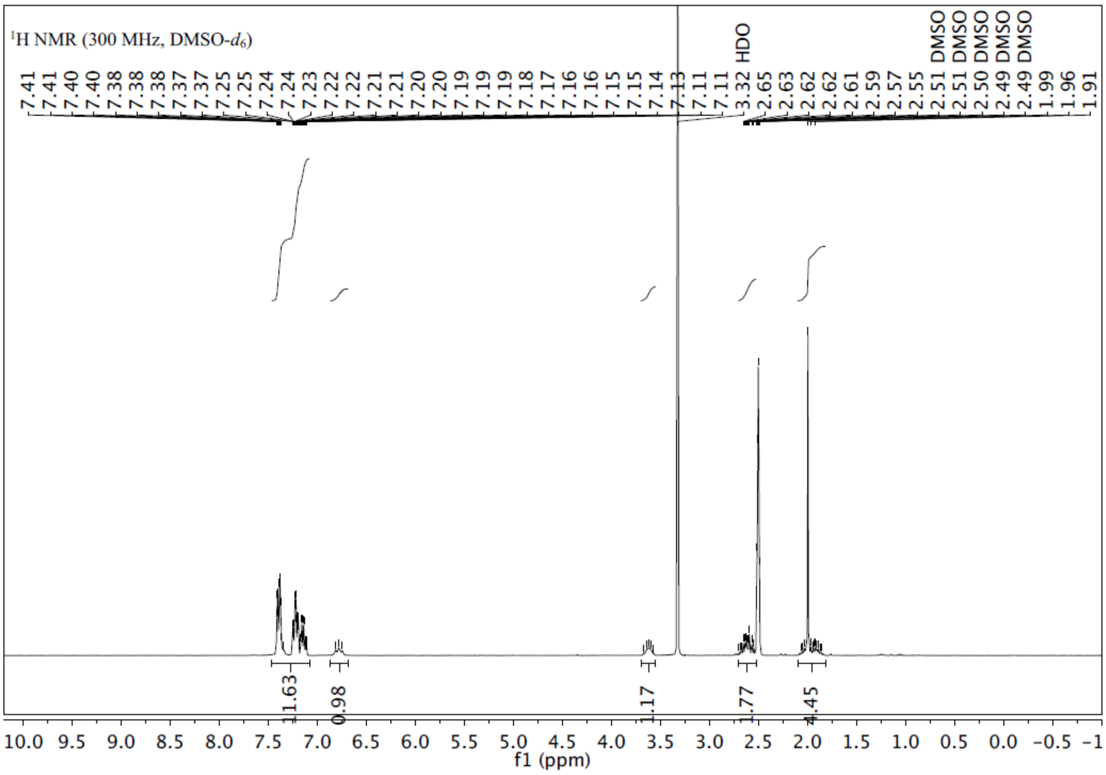
^{1}H NMR (300 MHz, DMSO-d_6)
7.41 7.41 7.40 7.40 7.38 7.38 7.38 7.37 7.37 7.25 7.25 7.24 7.24 7.23 7.22 7.22 7.21 7.21 7.20 7.20 7.19 7.19 7.19 7.18 7.17 7.16 7.16 7.15 7.15 7.14 7.13 7.11 7.11
3.32 HDO
2.65 2.63 2.62 2.62 2.61 2.59 2.57 2.55
2.51 DMSO 2.51 DMSO 2.50 DMSO 2.49 DMSO 2.49 DMSO
1.99 1.96 1.91
11.63
0.98
1.17
1.77
4.45
10.0 9.5 9.0 8.5 8.0 7.5 7.0 6.5 6.0 5.5 5.0 4.5 4.0 3.5 3.0 2.5 2.0 1.5 1.0 0.5 0.0 −0.5 −1
f1 (ppm)

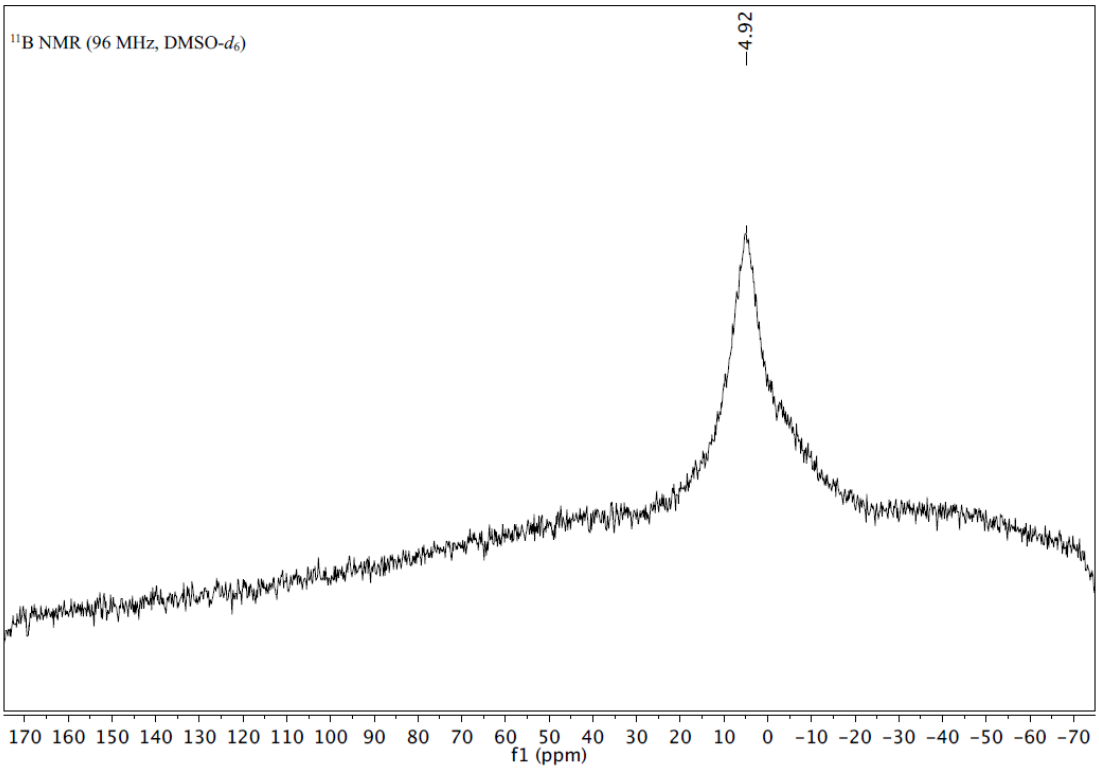
^{11}B NMR (96 MHz, DMSO-d_6)
4.92
170 160 150 140 130 120 110 100 90 80 70 60 50 40 30 20 10 0 −10 −20 −30 −40 −50 −60 −70
f1 (ppm)

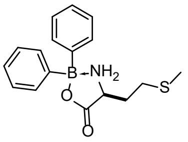

Met-2APB

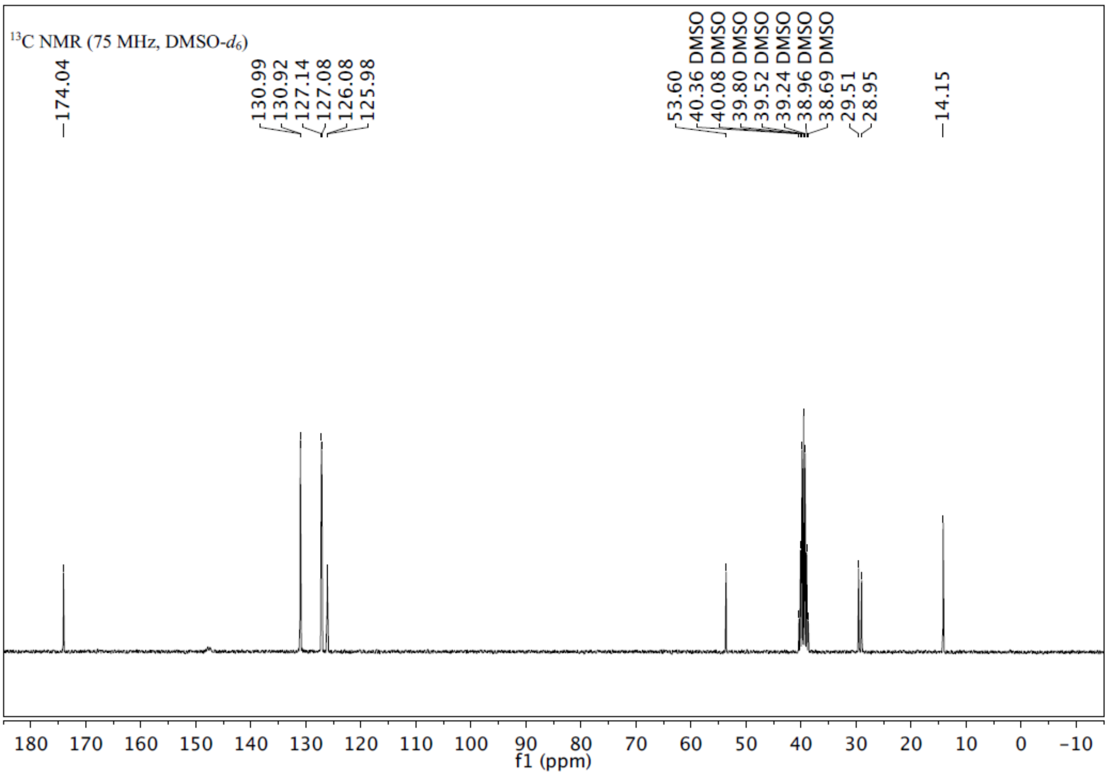

^{13}C NMR (75 MHz, DMSO-d_6)
174.04
130.99
130.92
127.14
127.08
126.08
125.98
53.60
40.36 DMSO
40.08 DMSO
39.80 DMSO
39.52 DMSO
39.24 DMSO
38.96 DMSO
38.69 DMSO
29.51
28.95
14.15
180 170 160 150 140 130 120 110 100 90 80 70 60 50 40 30 20 10 0 −10
f1 (ppm)

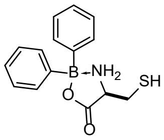
B
NH_2
SH
O
O

Cys-2APB

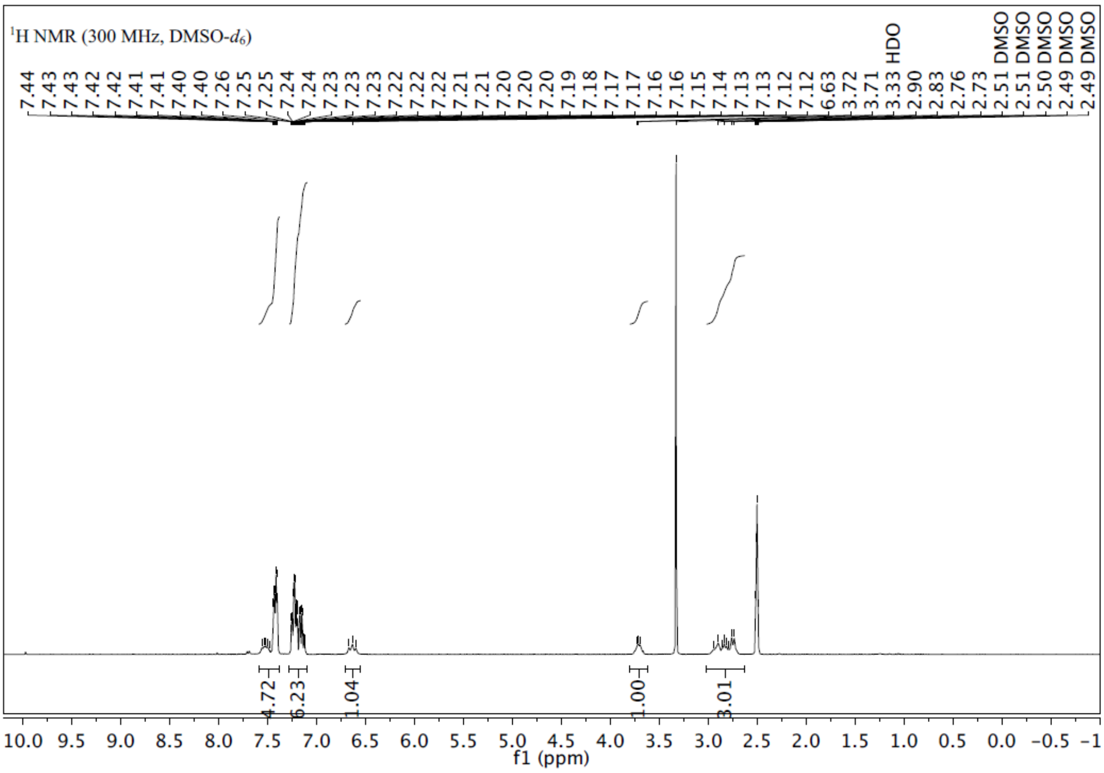
^{1}H NMR (300 MHz, DMSO-d_6)
7.44
7.43
7.43
7.42
7.42
7.41
7.41
7.40
7.40
7.26
7.25
7.25
7.24
7.24
7.23
7.23
7.23
7.22
7.22
7.22
7.21
7.21
7.20
7.20
7.20
7.19
7.18
7.17
7.17
7.16
7.16
7.15
7.14
7.13
7.13
7.12
7.12
6.63
3.72
3.71
3.33 HDO
2.90
2.83
2.76
2.73
2.51 DMSO
2.51 DMSO
2.50 DMSO
2.49 DMSO
2.49 DMSO
4.72
6.23
1.04
1.00
3.01
10.0 9.5 9.0 8.5 8.0 7.5 7.0 6.5 6.0 5.5 5.0 4.5 4.0 3.5 3.0 2.5 2.0 1.5 1.0 0.5 0.0 −0.5 −1
f1 (ppm)

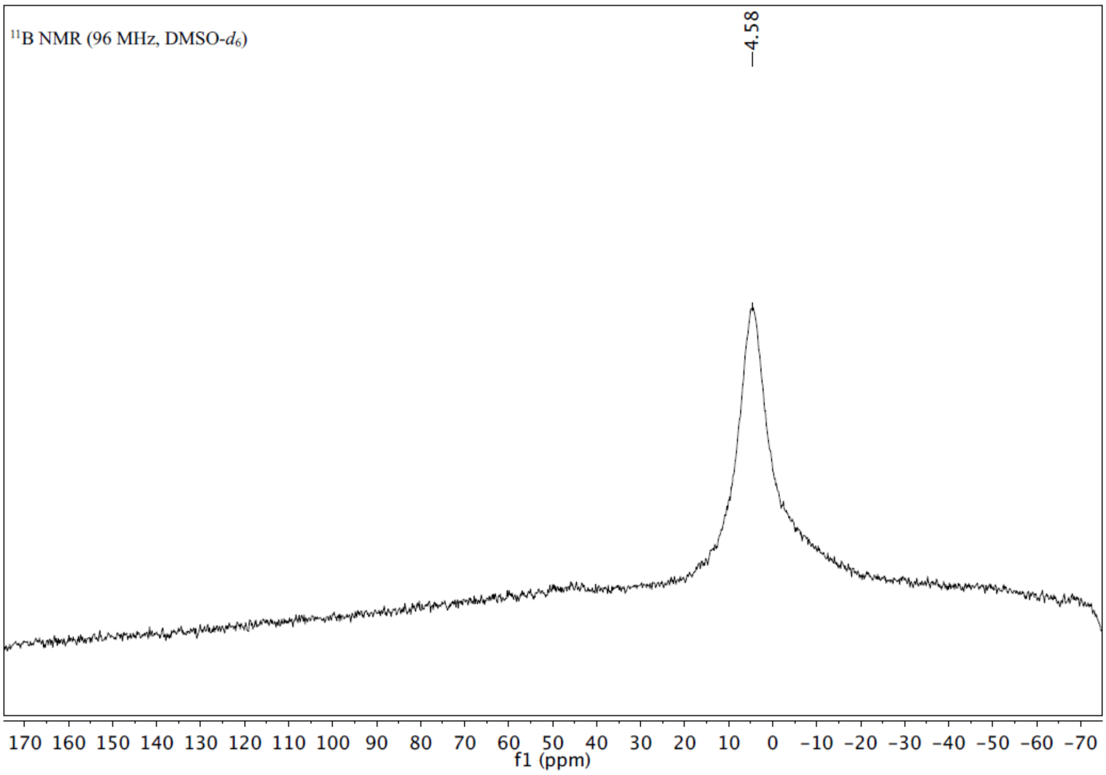
^{11}B NMR (96 MHz, DMSO-d_6)
4.58
170 160 150 140 130 120 110 100 90 80 70 60 50 40 30 20 10 0 −10 −20 −30 −40 −50 −60 −70
f1 (ppm)

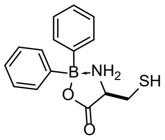

Cys-2APB

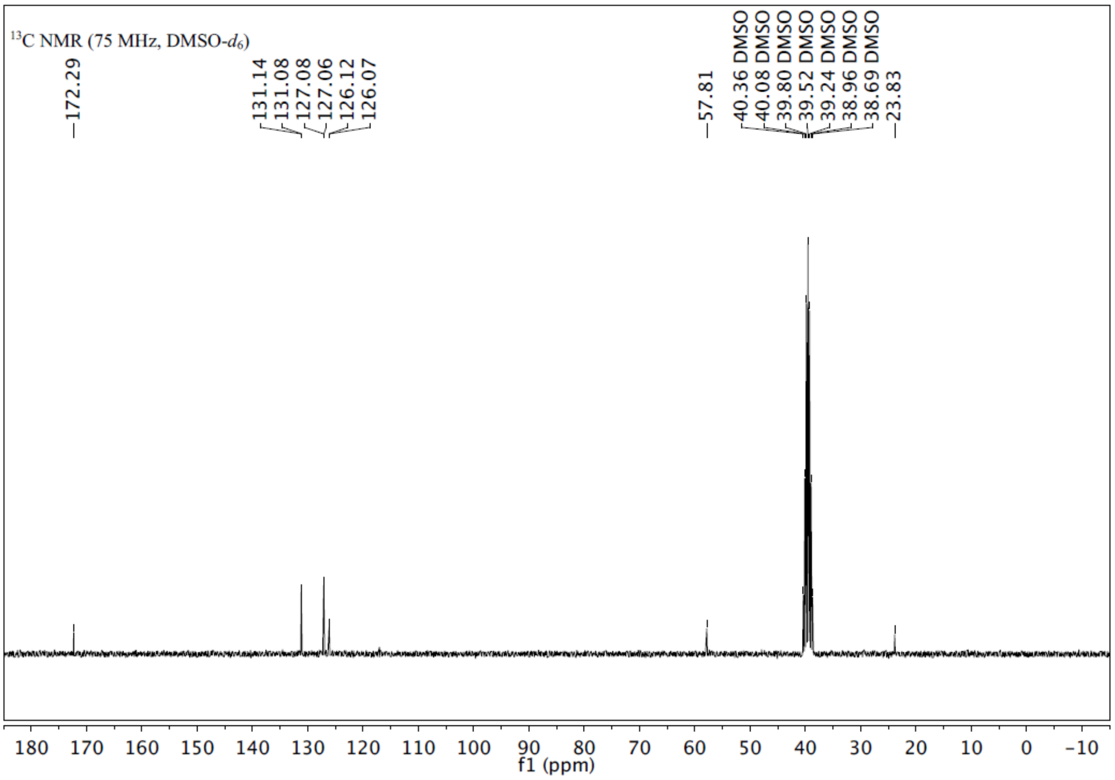

^{13}C NMR (75 MHz, DMSO-d_6)
172.29
131.14
131.08
127.08
127.06
126.12
126.07
57.81
40.36 DMSO
40.08 DMSO
39.80 DMSO
39.52 DMSO
39.24 DMSO
38.96 DMSO
38.69 DMSO
23.83
180 170 160 150 140 130 120 110 100 90 80 70 60 50 40 30 20 10 0 -10
f1 (ppm)

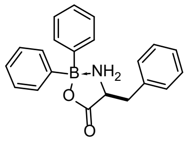
NH2
B
O
O

Phe-2APB

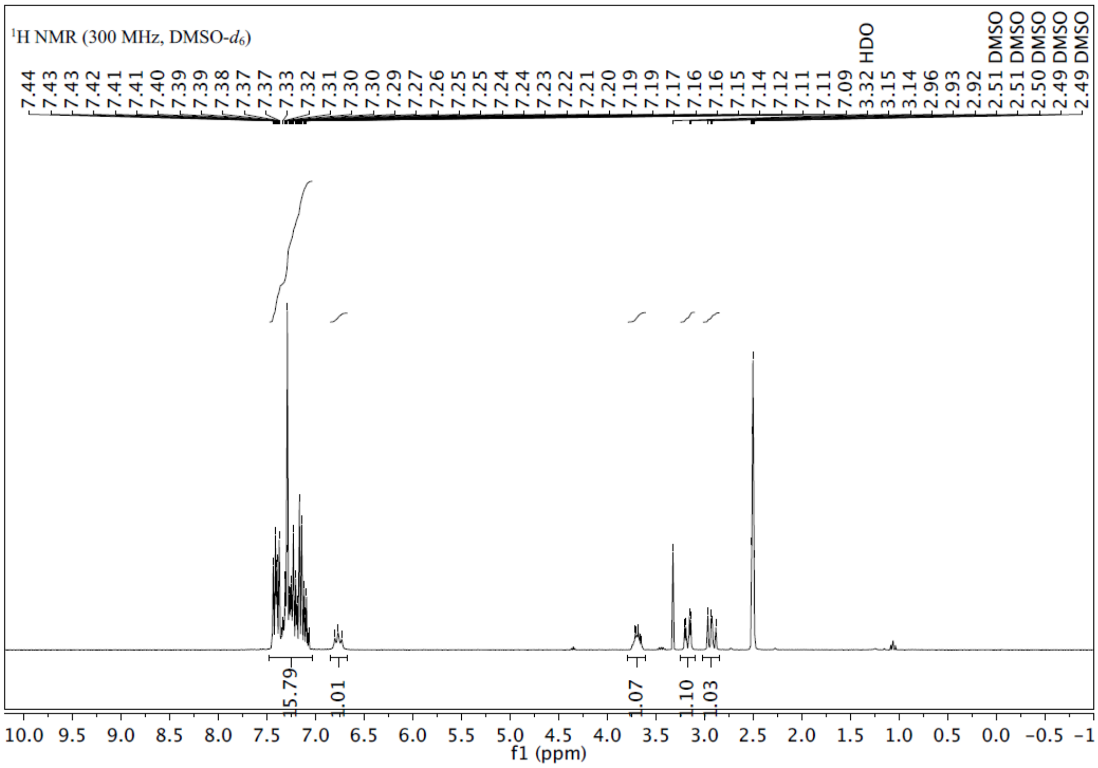
^{1}H NMR (300 MHz, DMSO-d_6)
7.44 7.43 7.43 7.42 7.41 7.41 7.40 7.39 7.39 7.38 7.37 7.37 7.33 7.32 7.31 7.30 7.30 7.29 7.27 7.26 7.25 7.25 7.24 7.24 7.23 7.22 7.21 7.20 7.19 7.19 7.17 7.16 7.16 7.15 7.14 7.12 7.11 7.11 7.09
3.32 HDO
3.15 3.14 2.96 2.93 2.92
2.51 DMSO 2.51 DMSO 2.50 DMSO 2.49 DMSO 2.49 DMSO
15.79
1.01
1.07
1.10
1.03
10.0 9.5 9.0 8.5 8.0 7.5 7.0 6.5 6.0 5.5 5.0 4.5 4.0 3.5 3.0 2.5 2.0 1.5 1.0 0.5 0.0 −0.5 −1
f1 (ppm)

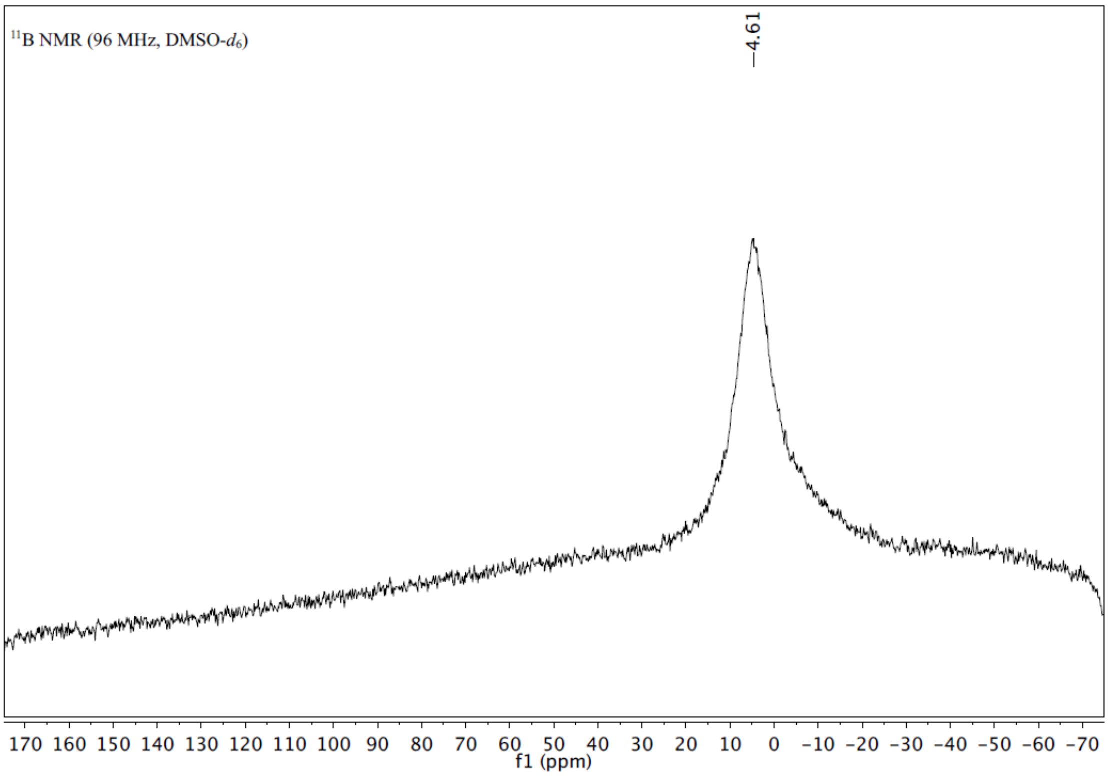
^{11}B NMR (96 MHz, DMSO-d_6)
4.61
170 160 150 140 130 120 110 100 90 80 70 60 50 40 30 20 10 0 −10 −20 −30 −40 −50 −60 −70
f1 (ppm)

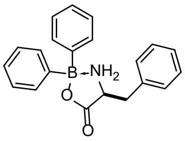

Phe-2APB

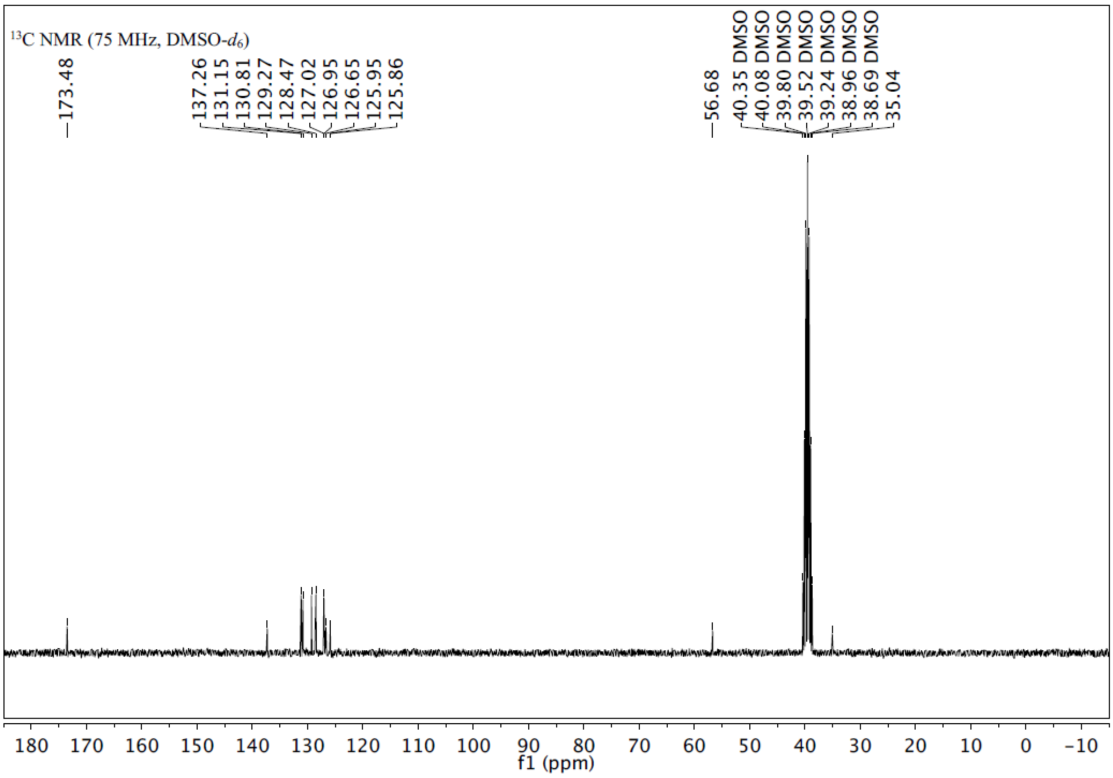

13C NMR (75 MHz, DMSO-d6)
173.48
137.26
131.15
130.81
129.27
128.47
127.02
126.95
126.65
125.95
125.86
56.68
40.35 DMSO
40.08 DMSO
39.80 DMSO
39.52 DMSO
39.24 DMSO
38.96 DMSO
38.69 DMSO
35.04
180 170 160 150 140 130 120 110 100 90 80 70 60 50 40 30 20 10 0 -10
f1 (ppm)

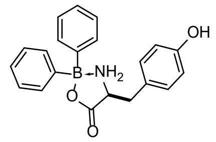

Tyr-2APB

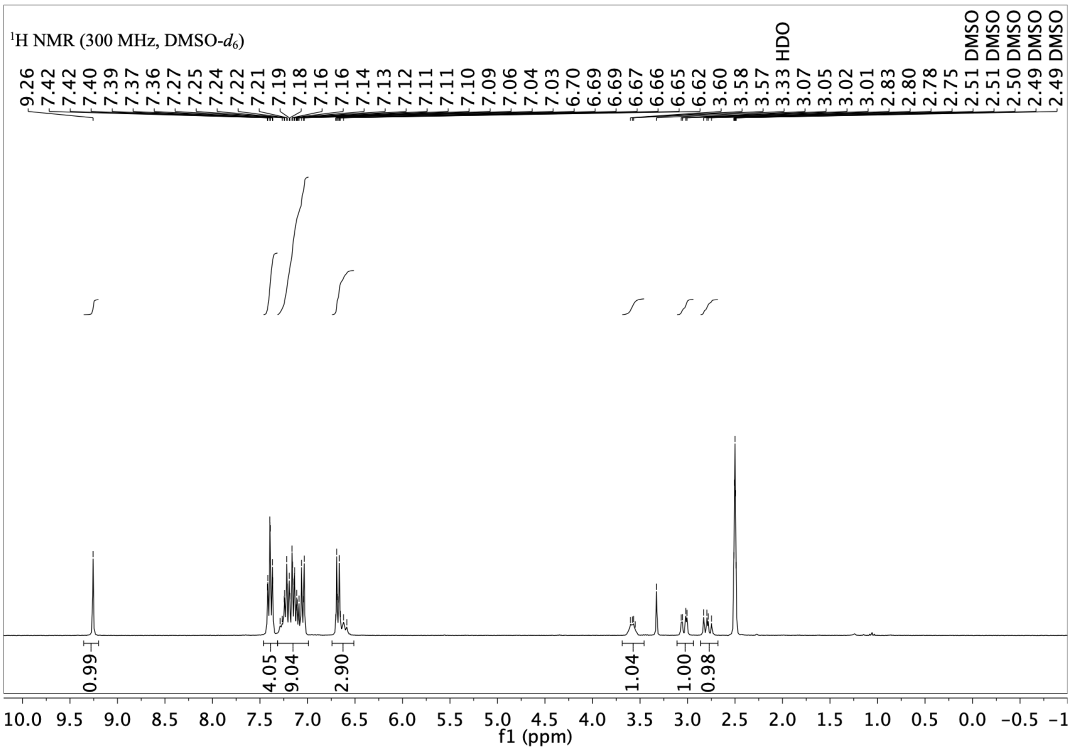

1H NMR (300 MHz, DMSO-d6)
9.26
7.42
7.42
7.40
7.39
7.37
7.36
7.27
7.25
7.24
7.22
7.21
7.19
7.18
7.16
7.14
7.13
7.12
7.11
7.11
7.10
7.09
7.06
7.04
7.03
6.70
6.69
6.69
6.67
6.66
6.65
6.62
3.60
3.58
3.57
3.33 HDO
3.07
3.05
3.02
3.01
2.83
2.80
2.78
2.75
2.51 DMSO
2.51 DMSO
2.50 DMSO
2.49 DMSO
2.49 DMSO
0.99
4.05
9.04
2.90
1.04
1.00
0.98
10.0 9.5 9.0 8.5 8.0 7.5 7.0 6.5 6.0 5.5 5.0 4.5 4.0 3.5 3.0 2.5 2.0 1.5 1.0 0.5 0.0 −0.5 −1
f1 (ppm)

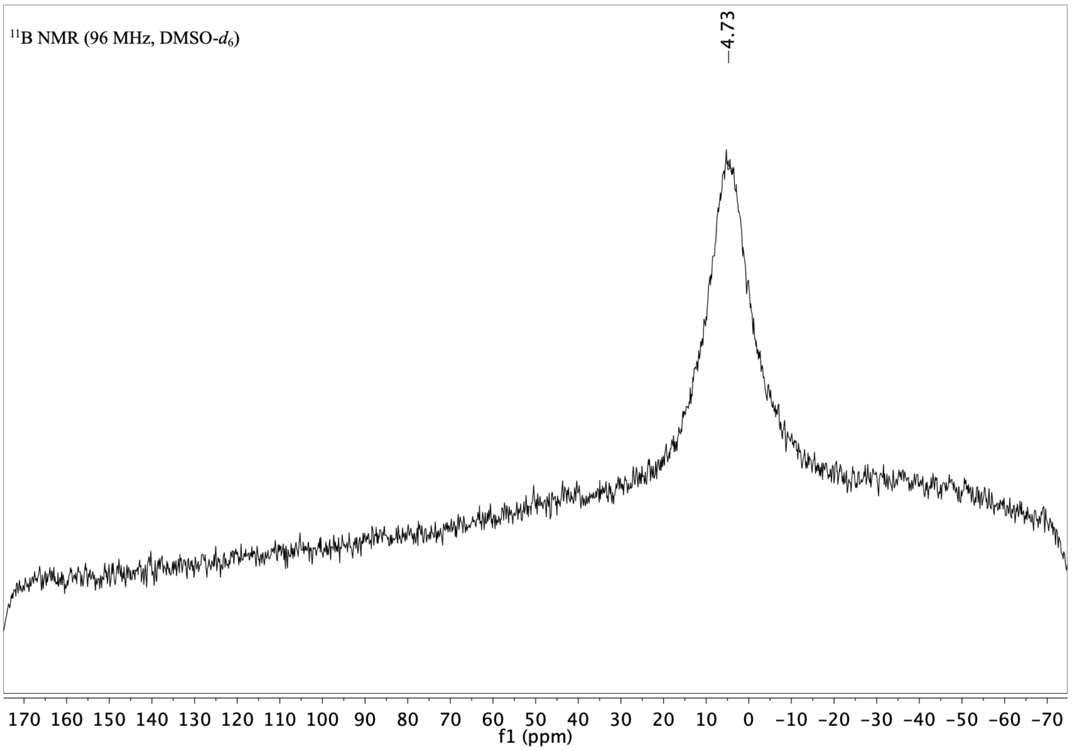

11B NMR (96 MHz, DMSO-d6)
4.73
170 160 150 140 130 120 110 100 90 80 70 60 50 40 30 20 10 0 −10 −20 −30 −40 −50 −60 −70
f1 (ppm)

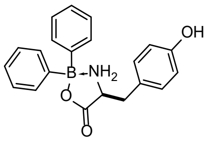
OH
B
NH2
O
O

Tyr-2APB

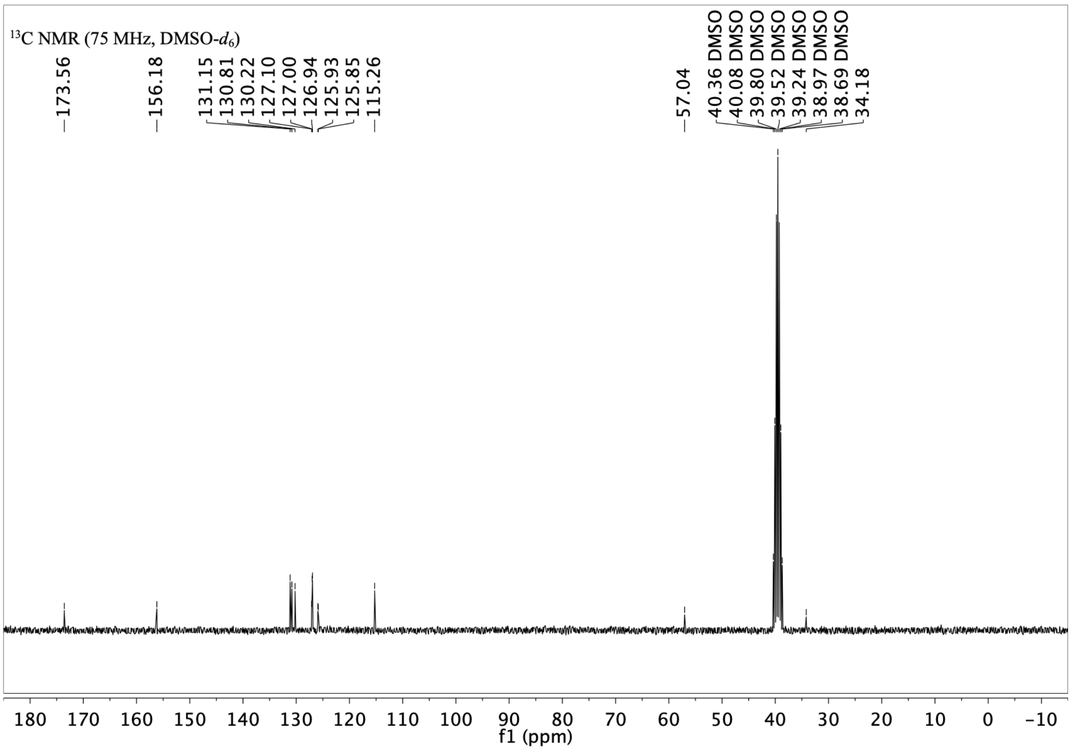
13C NMR (75 MHz, DMSO-d6)
173.56
156.18
131.15
130.81
130.22
127.10
127.00
126.94
125.93
125.85
115.26
57.04
40.36 DMSO
40.08 DMSO
39.80 DMSO
39.52 DMSO
39.24 DMSO
38.97 DMSO
38.69 DMSO
34.18
180 170 160 150 140 130 120 110 100 90 80 70 60 50 40 30 20 10 0 −10
f1 (ppm)

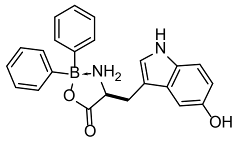
NH2
O
OH
H
N
B

5-HO-Trp-2APB

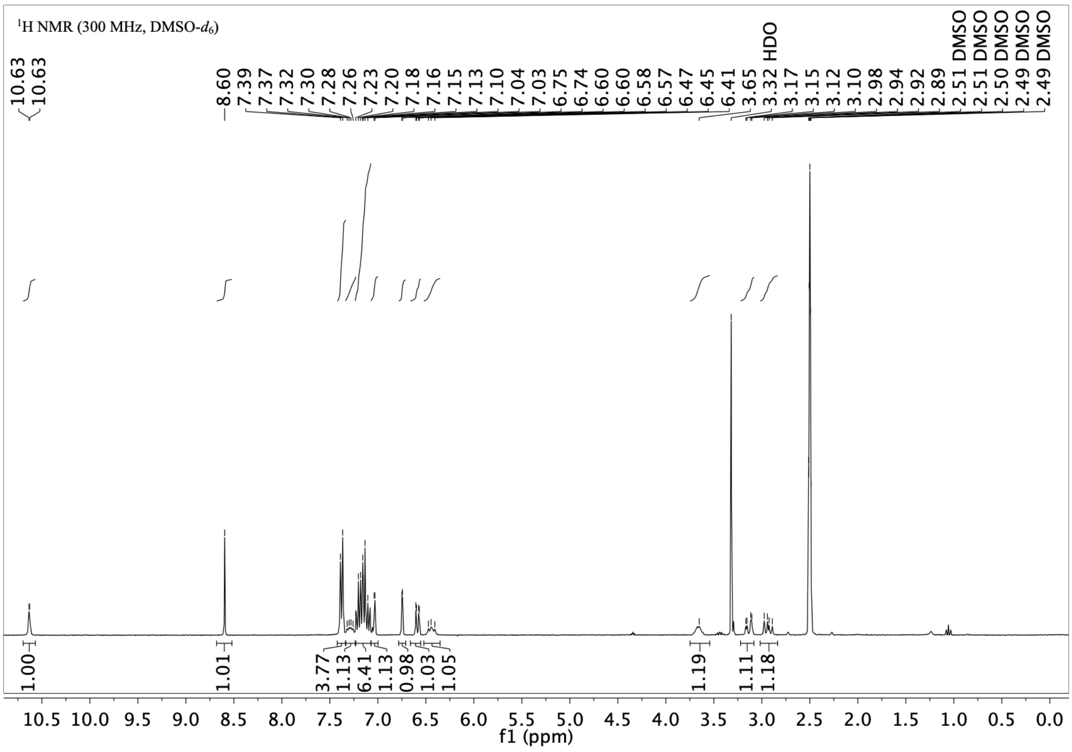
1H NMR (300 MHz, DMSO-d6)
f1 (ppm)

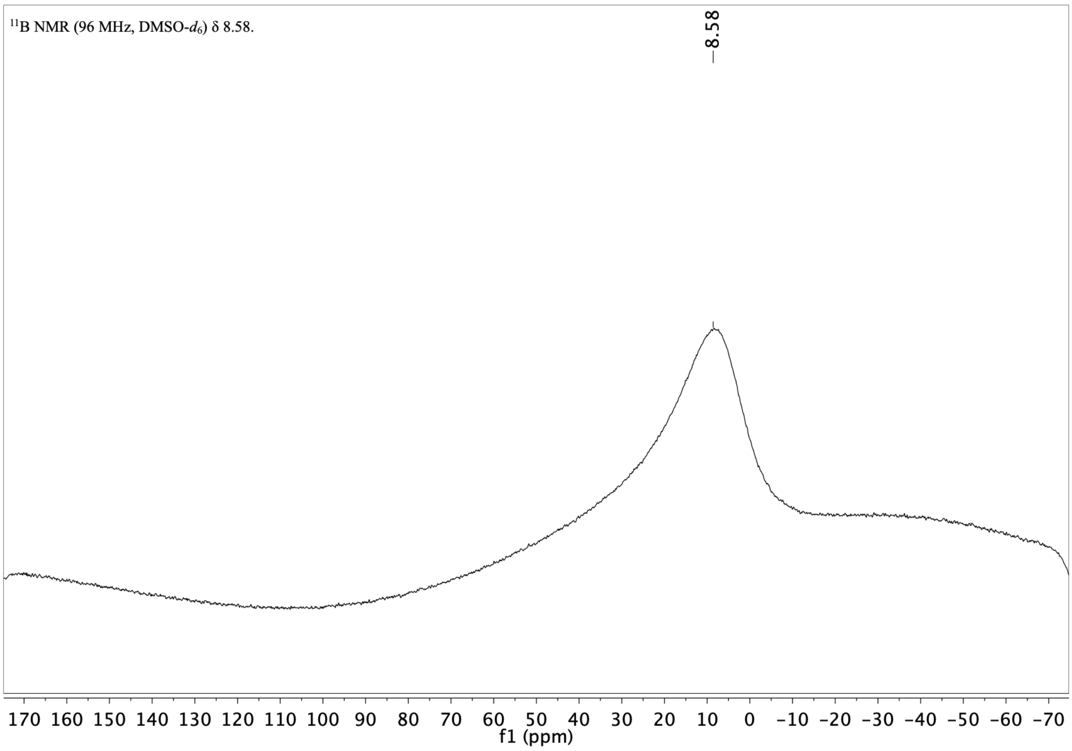
11B NMR (96 MHz, DMSO-d6) δ 8.58.
8.58
f1 (ppm)

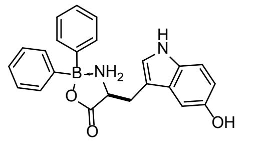

5-HO-Trp-2APB

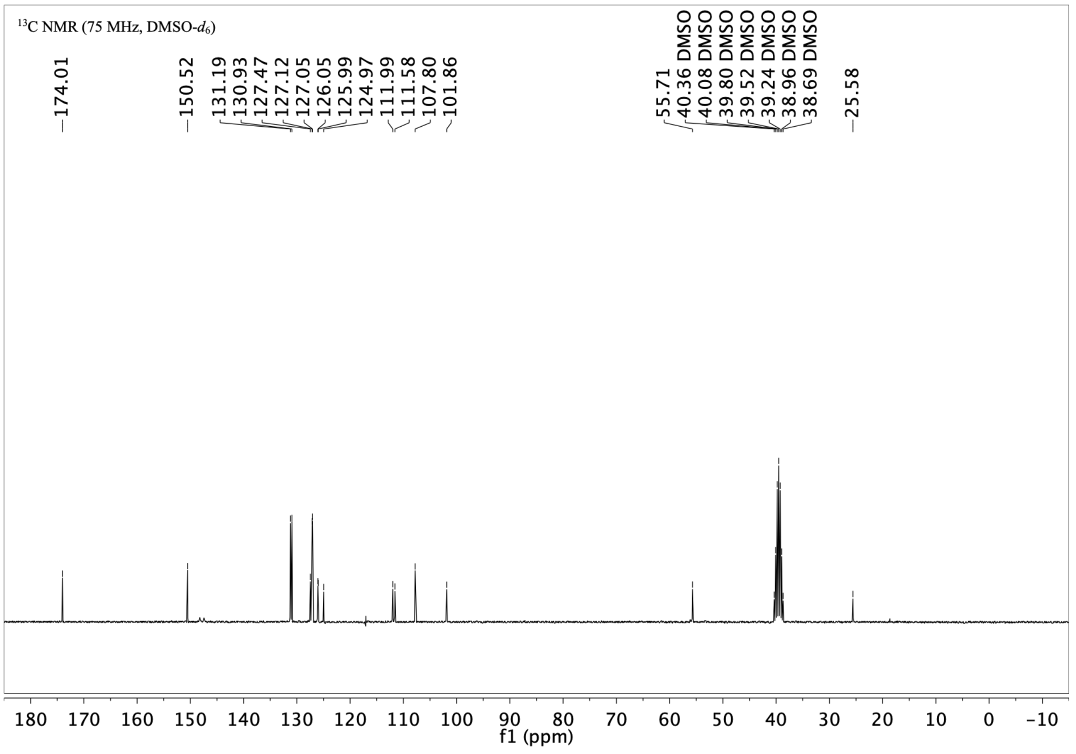

13C NMR (75 MHz, DMSO-d6)
174.01
150.52
131.19
130.93
127.47
127.12
127.05
126.05
125.99
124.97
111.99
111.58
107.80
101.86
55.71
40.36 DMSO
40.08 DMSO
39.80 DMSO
39.52 DMSO
39.24 DMSO
38.96 DMSO
38.69 DMSO
25.58
180 170 160 150 140 130 120 110 100 90 80 70 60 50 40 30 20 10 0 −10
f1 (ppm)

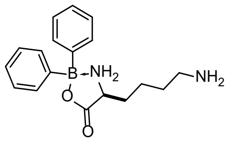
B
NH2
O
O
NH2

Lys-2APB

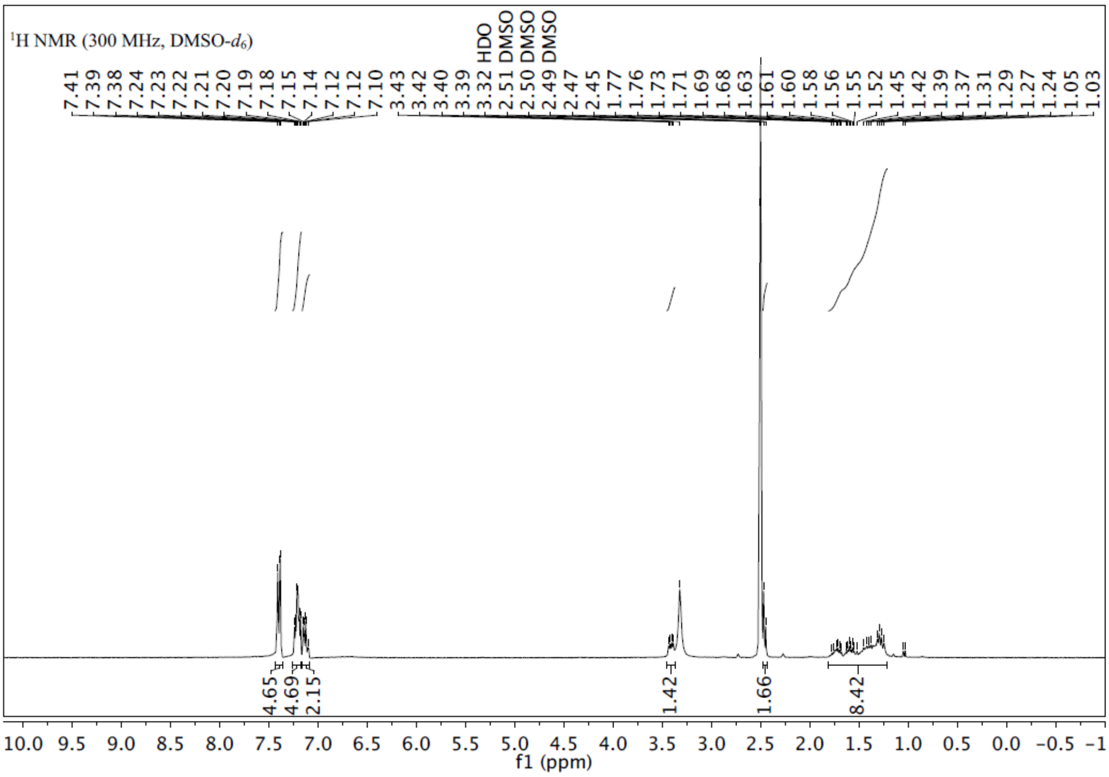
1H NMR (300 MHz, DMSO-d6)
7.41
7.39
7.38
7.24
7.23
7.22
7.21
7.20
7.19
7.18
7.15
7.14
7.12
7.10
3.43
3.42
3.40
3.39
3.32 HDO
2.51 DMSO
2.50 DMSO
2.49 DMSO
2.47
2.45
1.77
1.76
1.73
1.71
1.69
1.68
1.63
1.61
1.60
1.58
1.56
1.55
1.52
1.45
1.42
1.39
1.37
1.31
1.29
1.27
1.24
1.05
1.03
4.65
4.69
2.15
1.42
1.66
8.42
10.0 9.5 9.0 8.5 8.0 7.5 7.0 6.5 6.0 5.5 5.0 4.5 4.0 3.5 3.0 2.5 2.0 1.5 1.0 0.5 0.0 −0.5 −1
f1 (ppm)

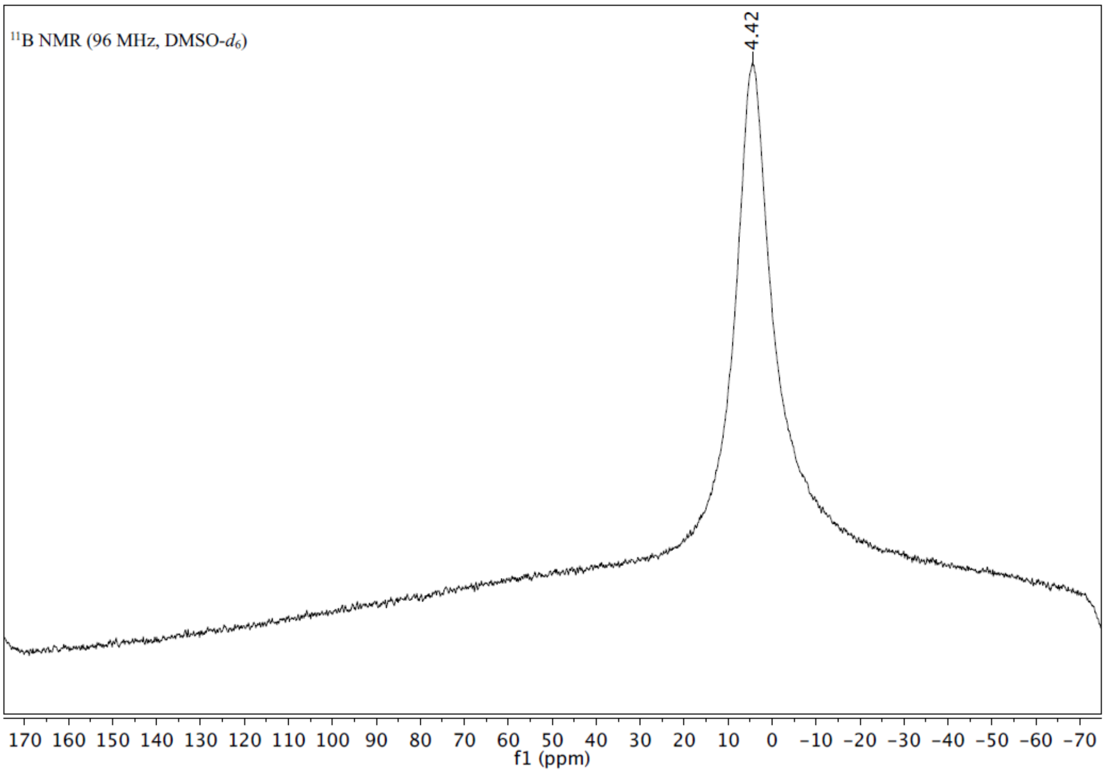
11B NMR (96 MHz, DMSO-d6)
4.42
170 160 150 140 130 120 110 100 90 80 70 60 50 40 30 20 10 0 −10 −20 −30 −40 −50 −60 −70
f1 (ppm)

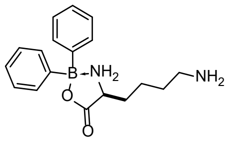

Lys-2APB

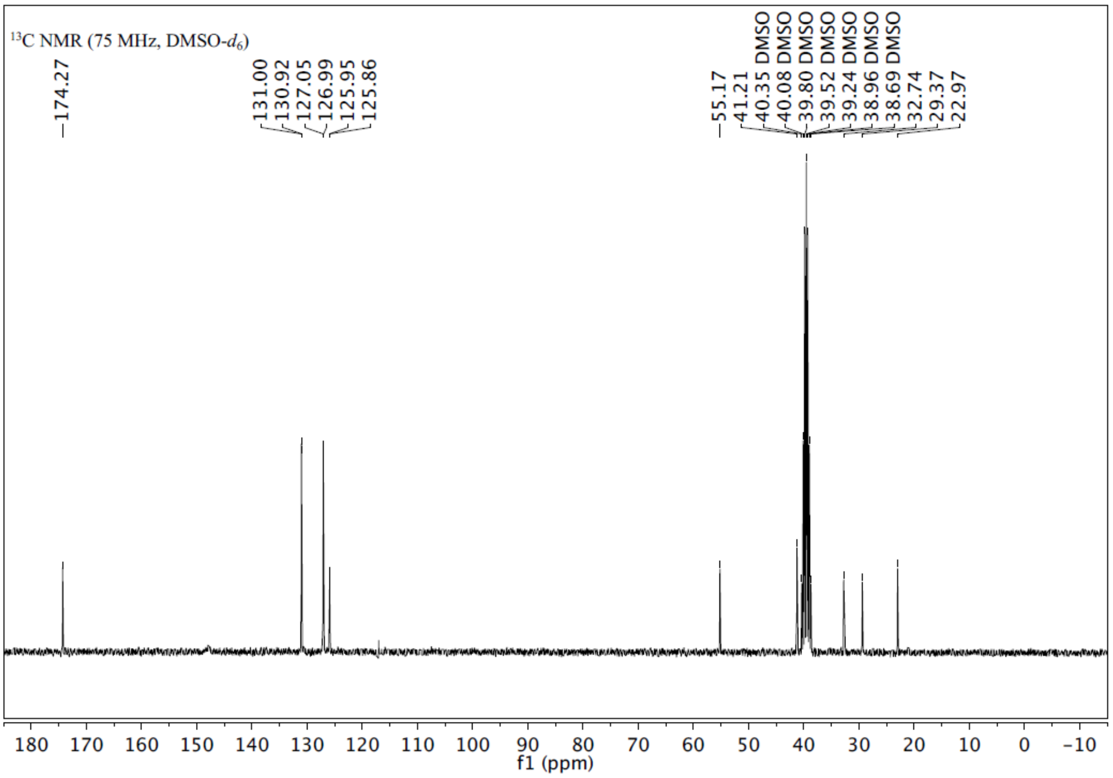

^{13}C NMR (75 MHz, DMSO-d_6)
174.27
131.00
130.92
127.05
126.99
125.95
125.86
55.17
41.21
40.35 DMSO
40.08 DMSO
39.80 DMSO
39.52 DMSO
39.24 DMSO
38.96 DMSO
38.69 DMSO
32.74
29.37
22.97
180 170 160 150 140 130 120 110 100 90 80 70 60 50 40 30 20 10 0 -10
f1 (ppm)
